# Aqueous Artemisia argyi extract mitigates acute lung injury *in association with* coordinated alterations in gut microbiota, metabolic homeostasis, and pulmonary inflammatory gene expression

**DOI:** 10.3389/fimmu.2026.1770675

**Published:** 2026-04-02

**Authors:** Huixiang Wang, Baoqin Long, Yaqi Cui, Yiliyaer Abulajiang, Beibei Chen, Xiaoxue Chen, Xue Hu, Chao Wang, Min Ren, Ruiping She, Xuna Ding, Haihong Jiao

**Affiliations:** 1 College of Animal Science and Technology, Tarim University, Alaer, Xinjiang, China; 2 Key Laboratory of Livestock and Forage Resources Utilization around Tarim, Ministry of Agriculture and Rural Areas, Alaer, Xinjiang, China; 3 School of Biological Sciences at Tarim University, Key Laboratory of Conservation and Utilization of Biological Resources in the Tarim Basin, Alaer, Xinjiang, China; 4 Laboratory of Animal Pathology and Public Health, Key Laboratory of Zoonosis of Ministry of Agriculture, College of Veterinary Medicine, China Agricultural University, Beijing, China

**Keywords:** acute lung injury, Artemisia argyi, immunometabolism, LPS, multi-omics

## Abstract

**Background:**

Artemisia argyi is a traditional medicinal herb with established anti-inflammatory and immunomodulatory properties. Its aqueous extract (AEAA), enriched in water-soluble bioactive constituents, exhibits favorable safety and bioavailability; however, its potential protective effects against acute lung injury (ALI) and its associations with systemic immunometabolic regulation remain incompletely understood.

**Methods:**

An LPS-induced ALI mouse model was established following 28 days of AEAA pretreatment. Lung histopathology, pulmonary edema, and inflammatory cytokines were evaluated. Integrated multi-omics analyses—including gut microbiota profiling, untargeted metabolomics of colonic contents and serum, and lung transcriptomics—were performed to characterize treatment-associated microbial, metabolic, and transcriptional alterations.

**Results:**

AEAA pretreatment dose-dependently alleviated lung injury, reduced pulmonary edema, and suppressed pro-inflammatory cytokines while restoring anti-inflammatory IL-10 levels. AEAA treatment was associated with partial reversal of LPS-induced gut dysbiosis, characterized by reduced abundance of inflammation-associated taxa and enrichment of beneficial genera, particularly Akkermansia and Lactobacillus. Metabolomic analyses revealed treatment-associated normalization of intestinal and systemic metabolic disturbances, including increased homeostasis-related metabolites and reduced inflammation-associated metabolites. Lung transcriptomic profiling suggested attenuation of LPS-associated transcriptional signatures related to NF-κB, MAPK, Toll-like receptor, and PI3K–AKT signaling pathways. Cross-omics integration further revealed coordinated associations among microbial shifts, metabolic remodeling, and pulmonary inflammatory gene expression.

**Conclusion:**

These findings suggest that aqueous Artemisia argyi extract is associated with mitigation of LPS-induced acute lung injury, accompanied by coordinated alterations in gut microbiota composition, host metabolic profiles, and pulmonary inflammatory gene expression. Although causal relationships were not established, this integrated multi-omics analysis provides a systems-level, hypothesis-generating framework supporting the potential of AEAA as a multi-target botanical candidate for ALI.

## 1 Introduction

Acute lung injury (ALI) and its more severe form, acute respiratory distress syndrome (ARDS), remain significant challenges in respiratory medicine due to their high global morbidity and mortality (1, 2). More than 3 million individuals develop ARDS each year, with hospital mortality rates ranging from 30% to 45% and rising to nearly 60% in severe cases requiring mechanical ventilation. ALI is marked by disruption of the alveolar–capillary barrier, pulmonary edema, and impaired gas exchange, ultimately reducing lung compliance (3). Growing evidence indicates that ALI/ARDS is not merely a lung-restricted condition but a systemic disorder driven by widespread immune dysregulation (4–6). A hallmark of ALI/ARDS is the aberrant activation of innate immunity, particularly excessive neutrophil infiltration and the release of pro-inflammatory cytokines such as TNF-α, IL-1β, and IL-6, which amplify systemic inflammation and contribute to sustained tissue injury (5).

Recent advances, particularly in gut–lung axis research, have shown that microbially derived metabolites are key regulators of systemic immunity and susceptibility to acute lung injury (7–10). Beneficial taxa such as *Akkermansia muciniphila* and *Lactobacillus* ferment plant polysaccharides into metabolites (e.g., arabinogalactose and short-chain fatty acids) that strengthen the epithelial barrier, enhance mucin turnover, and suppress proinflammatory signaling through NF-κB and excessive neutrophil recruitment (11–13). In contrast, dysbiosis—marked by the expansion of pathogenic or proinflammatory bacteria—leads to the buildup of metabolites such as N-acetylcadaverine, which drive epithelial stress and mucosal inflammation (14, 15). Systemic metabolites like itaconic acid, produced by activated macrophages, further reflect and amplify innate immune activation, whereas gut microbial modulation of host neurotransmitters (e.g., serotonin) shapes immune tone via the 5-HT and AhR pathways (16, 17). Collectively, these findings highlight the tight link between gut microbial and metabolic alterations and pulmonary inflammatory responses, although the causal direction remains not fully defined.

Modern pharmacological research indicates that A. argyi extracts can reduce inflammatory responses by inhibiting key mediators and signaling pathways in macrophages and other immune cells, suggesting potential relevance for respiratory inflammation and immune regulation (18, 19). Methanol extracts of A. argyi, for instance, have been shown to suppress inflammatory signaling in macrophages *in vitro*, lending scientific support to its traditional use as an anti-inflammatory herb (19). At the formulation level, traditional Chinese medicines such as Lianhua Qingwen—used extensively in China during the COVID-19 pandemic—have been examined in clinical studies and meta-analyses, where adjunctive use with standard therapy was associated with improved symptom relief in COVID-19 patients (20, 21). Collectively, these findings reflect growing scientific interest in herbal interventions for respiratory and viral infections, while also underscoring the need for more rigorous clinical trials to confirm their efficacy and safety.

Artemisia argyi (Chinese mugwort) has been used for centuries in Traditional Chinese Medicine in forms such as fumigation, decoctions, herbal baths, and moxa for moxibustion to dispel cold, alleviate pain, and support overall health (22–24). Its leaves are also incorporated into teas and commercial extracts for their bioactive constituents, including sesquiterpenoids, flavonoids, and volatile oils with notable anti-inflammatory and antibacterial activities (22, 25). Among these bioactive components, the aqueous extract of Artemisia argyi (AEAA) has attracted increasing attention due to its favorable safety profile, chemical stability, and bioavailability, particularly when compared with the volatile oil fraction, which is chemically unstable and has been reported to cause mucosal irritation and cytotoxicity at higher concentrations (22, 24, 25). AEAA is particularly rich in water-soluble polysaccharides and phenolic compounds that help suppress inflammation, mitigate oxidative stress, and regulate mucosal immunity with minimal side effects, making it a promising candidate for ALI intervention (18, 26).

Despite increasing evidence that Artemisia argyi–derived preparations protect against inflammatory lung injury, their mechanisms remain insufficiently defined. Most studies emphasize single endpoints—such as pulmonary histopathology or specific inflammatory mediators—providing limited insight into coordinated, system-level immunometabolic regulation (18, 19, 27, 28). Notably, whether AEAA simultaneously influences gut microbial composition, host metabolic patterns, and pulmonary inflammatory responses has not been systematically examined, and integrative multi-omics analyses linking these cross-compartment changes are still largely lacking.

Here, we used an integrated multi-omics approach to assess how AEAA pretreatment shapes gut microbial composition, host metabolic profiles, and pulmonary inflammatory gene expression in an LPS-induced acute lung injury model. By analyzing coordinated shifts across microbial, metabolic, and transcriptomic layers, this study offers a systems-level perspective on the immunomodulatory effects of AEAA across distinct biological compartments. Overall, our findings indicate that water-extractable Artemisia argyi fractions hold promise as multi-target immunomodulatory agents and reveal key immunometabolic features relevant to acute lung injury.

## 2 Materials and methods

### 2.1 Reagents and materials

*Artemisia argyi* was grown in Tarim Basin (Xinjiang, China) and harvested on June 10, 2024. Isoflurane (Cat. No. I8000) was purchased from Solarbio (Beijing, China). C57BL/6 mice were obtained from the Experimental Animal Center (Xinjiang Medical University, Urumqi, China). Lipopolysaccharide (LPS, Cat. No. CL6891) (29) was provided by Coolaber (Beijing, China). RNA extraction kit (Cat. No. R0026) was purchased from Beyotime Biotechnology Co., Ltd. (Shanghai, China). The RNA reverse transcription kit was obtained from ABM (Cat. #G592, Vancouver, Canada). Taq Pro Universal SYBR qPCR Master Mix (Q712-03) was purchased from Vazyme Biotech Co., Ltd. (Nanjing, China). Enzyme-linked immunosorbent assay (ELISA) kits for mouse IL-1β (Cat. #EK201B), IL-6 (Cat. #EK206), IL-10 (Cat. #EK210), TNF-α (Cat. #EK282), and myeloperoxidase (MPO) (Cat. #EK2133) were obtained from Multisciences (Lianke) Biotech Co., Ltd. (Hangzhou, China). All other reagents were of analytical grade and used without further purification.

### 2.2 Preparation of aqueous extract of *Artemisia argyi*

The dried leaves of *Artemisia argyi* were extracted by hot-water extraction under the following conditions: extraction temperature 100 °C, extraction time 2.27 h, water-to-material ratio 21.5 mL/g, and then freeze-dried. The experimental AEAA was diluted in saline and filtered through a 0.22 μm membrane filter (18, 25).

#### 2.2.1 NMR spectroscopy

The aqueous extract of *Artemisia argyi* was freeze-dried, and an appropriate amount of the dried sample was dissolved in deuterated dimethyl sulfoxide (DMSO-d_6_) and thoroughly mixed prior to analysis. Nuclear magnetic resonance (NMR) experiments, including one-dimensional ^1^H-NMR and ^13^C-NMR as well as two-dimensional HSQC-TOCSY spectra, were recorded using a Bruker 500 MHz NMR spectrometer (Bruker, Germany) to characterize the major chemical constituents of the aqueous extract.

### 2.3 Animals and treatments

A Seven-week-old male C57BL/6 mice were purchased from Xinjiang Medical University (Xinjiang, China). After a 7-day acclimatization period, mice were housed under specific pathogen-free conditions in top-filtered cages with a 12 h light/dark cycle and given ad libitum access to an AIN-76A diet and filtered drinking water. To minimize cage effects on gut microbiota composition, mice from each group were distributed across multiple cages with comparable numbers per cage (30, 31).

An aqueous extract of Artemisia argyi (AEAA) was used as a preventive intervention. AEAA was prepared from dried A. argyi leaves by hot-water extraction and administered via drinking water. Following acclimatization, mice were randomly assigned to five groups (n = 20 per group) using a random number table: control group, LPS model group, low-dose AEAA group (0.5 mg/mL AEAA + 5 mg/kg LPS), mid-dose AEAA group (1 mg/mL AEAA + 5 mg/kg LPS), and high-dose AEAA group (1.5 mg/mL AEAA + 5 mg/kg LPS). Mice received the corresponding treatments with free access to food and water for 28 consecutive days. On day 29, acute lung injury (ALI) was induced by intratracheal instillation of lipopolysaccharide (LPS, 5 mg/kg) in mice from the LPS model and AEAA-treated groups, while control mice received an equivalent volume of sterile saline (29, 32). All intratracheal procedures were performed by the same trained investigator using a standardized protocol.

This animal experiment was approved by the Ethics Committee of Tarim University (Approval No. 2024094) and conducted in accordance with relevant institutional guidelines and regulations, the ARRIVE 2.0 guidelines, and the recommendations of the American Veterinary Medical Association (AVMA) Guidelines for the Euthanasia of Animals (2020 Edition) (33).

Eight hours after LPS administration, mice were euthanized by an overdose of sodium pentobarbital administered via intraperitoneal injection at a dose of 150 mg/kg. Loss of the righting reflex, absence of pedal withdrawal reflex, and cessation of respiration and heartbeat were confirmed prior to tissue collection. Cervical dislocation was performed as a secondary physical method to ensure death.

### 2.4 Histological examination

After 8 h of LPS administration, mice were euthanized, and the left lung lobes were collected and fixed in 4% paraformaldehyde for 48 h. The fixed tissues were dehydrated through a graded ethanol series, cleared in xylene, and embedded in paraffin. Sections of 4 μm thickness were cut and mounted on glass slides. The sections were deparaffinized, rehydrated, and stained with hematoxylin and eosin (H&E) according to standard procedures to evaluate histopathological changes. Pathological images were observed and acquired under an optical microscope (Leica, Germany) at 200× magnification. Histopathological scoring and evaluation were performed in a blinded manner by investigators who were unaware of the group allocation (32).

### 2.5 Measurement of BALF

Bronchoalveolar lavage fluid (BALF) samples were centrifuged at 1000 rpm for 10 min at 4 °C, and the supernatants were collected for analysis. The concentrations of myeloperoxidase (MPO), tumor necrosis factor-α (TNF-α), interleukin-6 (IL-6), interleukin-1β (IL-1β), and interleukin-10 (IL-10) were determined using an enzyme-linked immunosorbent assay.

### 2.6 Serum analysis

The mouse plasma was centrifuged at 3000 rpm for 15 min to collect serum. The concentrations of myeloperoxidase (MPO), tumor necrosis factor-α (TNF-α), interleukin-6 (IL-6), interleukin-1β (IL-1β), and interleukin-10 (IL-10) were separately measured using ELISA kits (Multisciences, Hangzhou, China).

### 2.7 Quantitative QPCR

Total RNA was extracted from lung tissue using Total RNA extraction kit (Beyotime, Shanghai, China). Genes and primers used are shown in **Table 1**. Complementary DNA (cDNA) was extracted from total RNA by reverse transcription, and the resulting cDNA was diluted to 450ng/μL as the template for quantitative PCR. Amplification was performed using Taq Pro Universal SYBR qPCR Master Mix (Vazyme, Nanjing, China). The relative expression levels of target genes were normalized to β-actin and calculated using 2^-ΔΔCt method. Primer design was derived from the primer bank, as shown in **Table 1**.

**Table 1:** Sequences of primers used in the gene expression analysis.

Gene	Primer sequence (5′ to 3′)
IL1β	F:GAAATGCCACCTTTTGACAGTG R:TGGATGCTCTCATCAGGACAG
IL6	F:CTGCAAGAGACTTCCATCCAG R:AGTGGTATAGACAGGTCTGTTGG
IL10	F:CTTACTGACTGGCATGAGGATCA R:GCAGCTCTAGGAGCATGTGG
TNFα	F:CAGGCGGTGCCTATGTCTC R:CGATCACCCCGAAGTTCAGTAG
Cxcl2	F: CCAACCACCAGGCTACAGG R: GCGTCACACTCAAGCTCTG
TLR4	F:AAATGCACTGAGCTTTAGTGGT R:TGGCACTCATAATGATGGCAC
NFKBIA	F:TGAAGGACGAGGAGTACGAGC R:TGCAGGAACGAGTCTCCGT
MYD88	F: TCATGTTCTCCATACCCTTGGT R:AAACTGCGAGTGGGGTCAG
TNFAIP3	F:TGTGGGGTGTTCAGGATACTG R:GTTCCGAGTGTCTGTCTCCTTA
FOS	F:CGGGTTTCAACGCCGACTA R:TGGCACTAGAGACGGACAGAT
JUN	F:TTCCTCCAGTCCGAGAGCG R:TGAGAAGGTCCGAGTTCTTGG
MAPK1	F: GGTTGTTCCCAAATGCTGACT R:CAACTTCAATCCTCTTGTGAGGG
MAPK3	F:TCCGCCATGAGAATGTTATAGGC R: GGTGGTGTTGATAAGCAGATTGG
MAPK10	F:CAAGAGGGCTTACCGGGAG R:AGGTTGGCGTCCATCAGTTC
MAPK14	F:TGACCCTTATGACCAGTCCTTT R:GTCAGGCTCTTCCACTCATCTAT
CCND1	F:GCGTACCCTGACACCAATCTC R:ACTTGAAGTAAGATACGGAGGGC
AKT1	F:ATGAACGACGTAGCCATTGTG R:TTGTAGCCAATAAAGGTGCCAT
PIK3CA	F:CCACGACCATCTTCGGGTG R:GGGGAGTAAACATTCCACTAGGA
β-actin	F:GTGACGTTGACATCCGTAAAGA R:GCCGGACTCATCGTACTCC

### 2.8 Microbiome

Colonic contents were collected aseptically immediately after euthanasia and snap-frozen in liquid nitrogen, then stored at −80 °C until analysis. For gut microbiota analysis, representative samples were selected from each group (n = 5 per group). Microbial genomic DNA was extracted using a commercial fecal DNA extraction kit (QIAamp Fast DNA Stool Mini Kit, Qiagen, Germany) according to the manufacturer’s instructions. The V3–V4 hypervariable regions of the bacterial 16S rRNA gene were amplified by PCR using universal primers (forward 341F and reverse 806R). PCR products were purified, quantified, and sequenced on an Illumina MiSeq platform (Illumina, San Diego, CA, USA).

Raw sequencing data were processed using QIIME2 software (version 2023.2) to perform quality filtering, denoising, and chimera removal. Amplicon sequence variants (ASVs) were generated and taxonomically assigned against the SILVA reference database (Release 138). Alpha diversity indices (Chao1, Shannon, and Simpson) were calculated to assess microbial richness and evenness, while beta diversity was evaluated using principal coordinate analysis (PCoA) based on Bray–Curtis distances. Differences in microbial community composition among groups were assessed by analysis of similarities (ANOSIM). Differentially abundant taxa were identified using linear discriminant analysis effect size (LEfSe), with an LDA score threshold of >2.0. All microbiome analyses were conducted as exploratory assessments to characterize treatment-associated shifts in gut microbial composition (34–37).

### 2.9 Metabolome

Untargeted metabolomics analyses were performed on colonic contents and serum samples to investigate metabolic alterations associated with AEAA pretreatment in LPS-induced acute lung injury. Representative samples from each experimental group were selected for metabolomic profiling (n = 5 per group). Metabolite extraction, detection, and data acquisition were conducted using liquid chromatography–mass spectrometry (LC–MS) following standard procedures.

Raw mass spectrometry data were processed to normalize sample response intensities, and metabolites with a relative standard deviation (RSD) greater than 30% in quality control (QC) samples were excluded to ensure data reliability. The resulting dataset was log10-transformed prior to statistical analysis. Multivariate statistical analyses, including principal component analysis (PCA) and orthogonal partial least squares discriminant analysis (OPLS-DA), were applied to assess global metabolic differences among groups and model stability. Differential metabolites were identified based on variable importance in projection (VIP) scores greater than 1.0 and a significance threshold of *p < 0.05.* Metabolic pathway enrichment analysis was subsequently performed using the Kyoto Encyclopedia of Genes and Genomes (KEGG) database. All metabolomic analyses were conducted as exploratory investigations to characterize treatment-associated metabolic shifts rather than targeted quantitative validation (38–41).

### 2.10 Transcriptome

Total RNA was extracted from lung tissues using TRIzol reagent according to the manufacturer’s instructions. RNA integrity and quality were assessed using an Agilent 5300 Fragment Analyzer, and RNA concentration was measured with a NanoDrop ND-2000 spectrophotometer. For transcriptomic analysis, representative lung samples were selected from each experimental group (n = 5 per group). RNA sequencing libraries were constructed following standard protocols and sequenced on an Illumina platform.

Transcript abundance was quantified as transcripts per million (TPM). Differential gene expression analysis was performed using the DESeq2 package. Genes with an absolute log2 fold change (|log2FC|) ≥ 1 and a false discovery rate (FDR) ≤ 0.05 were considered significantly differentially expressed. Functional enrichment analyses of differentially expressed genes (DEGs) were conducted using Gene Ontology (GO) and Kyoto Encyclopedia of Genes and Genomes (KEGG) pathway databases to identify biological processes and signaling pathways associated with treatment-related transcriptional changes. Transcriptomic analyses were conducted as exploratory assessments to characterize alterations in pulmonary inflammatory gene expression at the mRNA level (41–43).

### 2.11 Data analysis

Statistical analyses were performed using GraphPad Prism (version 9.0) and R software (version 4.2.0). Data are presented as mean ± standard error of the mean (SEM). Normality of data distribution was assessed prior to statistical testing. For comparisons among multiple groups, one-way analysis of variance (ANOVA) followed by appropriate *post hoc* tests was applied. For non-parametric data, the Kruskal–Wallis test was used as indicated. A value of p < 0.05 was considered statistically significant.

For microbiome, metabolomics, and transcriptomics analyses, statistical testing and multivariate analyses were conducted using the respective bioinformatics pipelines described above. Multiple-testing correction was applied where appropriate, and false discovery rate (FDR)–adjusted p values were used for omics-based differential analyses. All omics analyses were conducted in an exploratory manner to identify treatment-associated patterns and associations (44).

## 3 Results

### 3.1 Structural characterization of major constituents by NMR analysis

**Figure 1:**
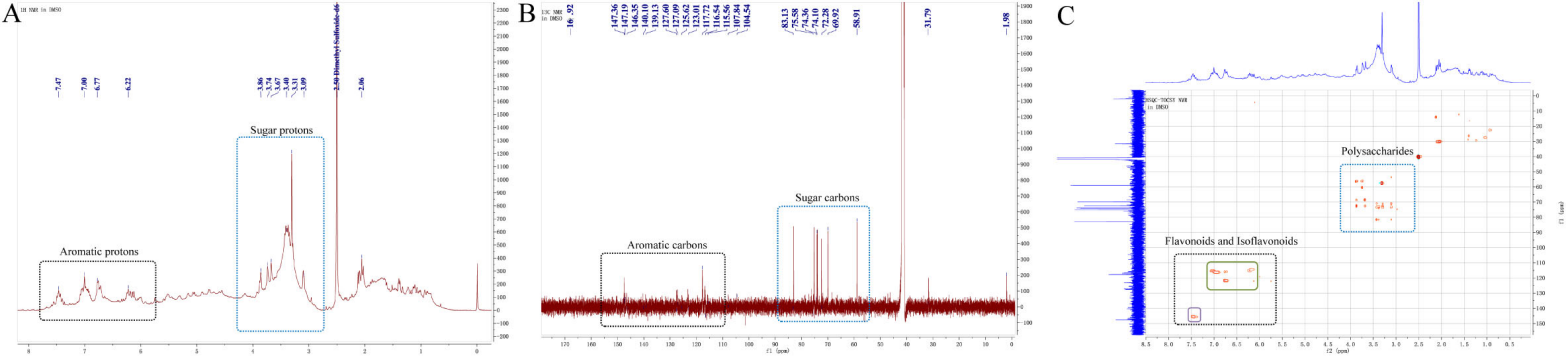
NMR spectra of the aqueous extract of *Artemisia argyi*. **(A)** ^1^H-NMR spectrum of the aqueous extract of *Artemisia argyi*. **(B)** ^13^C-NMR spectrum of the aqueous extract of *Artemisia argyi*. **(C)** HSQC-TOCSY spectrum of the aqueous extract of *Artemisia argyi*.

The 1H-NMR spectrum of the aqueous extract of Artemisia argyi (**Figure 1A**) showed that proton signals were mainly distributed in three chemical shift regions: δ 6.5–7.5, δ 3.0–4.0, and δ 1.0–2.5. Signals in the high-field region (δ 1.0–2.5) were attributed to protons from aliphatic chains, whereas resonances in the middle-field region (δ 3.0–4.0) were characteristic of carbohydrate-associated protons. The low-field signals observed at δ 6.5–7.5 corresponded to unsaturated or aromatic protons, indicating the presence of aromatic compounds. Overall, the ^1^H-NMR data suggested that carbohydrates and aromatic constituents were the dominant components in the aqueous extract, with only minor contributions from aliphatic compounds.

The ^13^C-NMR spectrum (**Figure 1B**) further supported these observations. Typical aliphatic carbon signals were largely absent, indicating a very low abundance of aliphatic compounds. Carbon resonances in the δ 110–145 region were therefore assigned to aromatic carbons rather than olefinic carbons of hydrocarbons, while signals in the δ 60–90 region were consistent with characteristic carbohydrate carbon atoms. These results were in good agreement with the ^1^H-NMR analysis.

In the HSQC-TOCSY spectrum (**Figure 1C**), clear correlations were observed between carbon signals in the δ 110–145 region and proton signals in the δ 6.5–7.5 region, confirming the presence of aromatic structural units. Notably, the relative signal distribution between the δ 110–125 and δ 130–145 regions was approximately 3:1, which is characteristic of flavonoid-type natural products. This observation suggests that flavonoids and isoflavonoids are present in the aqueous extract. In addition, distinct polysaccharide-related signal regions were observed in the HSQC-TOCSY spectrum, further corroborating the presence of carbohydrate components. Based on the combined ^1^H-NMR, ^13^C-NMR, and HSQC-TOCSY analyses, the major classes of natural products in the aqueous extract of Artemisia argyi was suggested to be enriched in polysaccharides and flavonoid-related constituents, including isoflavonoid-like structures.

### 3.2 AEAA pretreatment alleviates LPS-induced lung injury in mice

**Figure 2:**
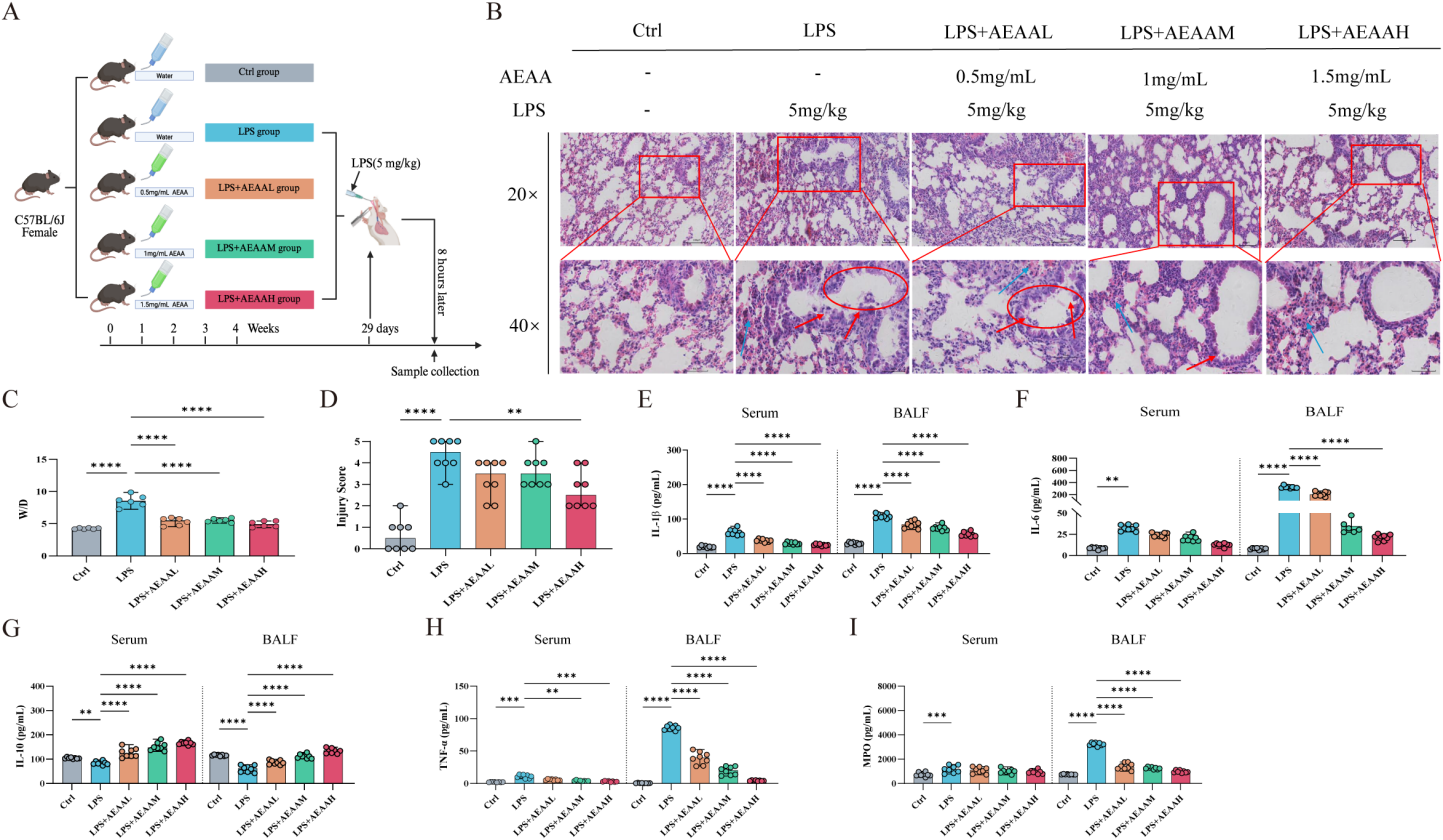
AEAA pretreatment is associated with reduced pathological features of LPS-induced acute lung injury in mice. **(A)** Experimental design of the LPS-induced acute lung injury (ALI) model. **(B)** Histopathological evaluation of lung tissues. Representative H&E-stained sections from different treatment groups at 20× and 40× magnification show alveolar wall thickening, inflammatory cell infiltration, hemorrhage, and edema in LPS-treated mice. AEAA treatment dose-dependently ameliorated these pathological alterations (red boxed areas). **(C)** Wet-to-dry (W/D) lung weight ratio. **(D)** Lung injury score. **(E–G)** Pro-inflammatory cytokines in serum and BALF.IL-1β **(E)**, IL-6 **(F)**, and IL-10 **(G)** levels in serum and BALF. **(H)** TNF-α levels in serum and BALF. **(I)** Myeloperoxidase (MPO) activity. Data are presented as mean ± SEM. n = 5 per group. Statistical analysis was performed using one-way ANOVA followed by *post hoc* tests. *P < 0.05, **P < 0.01, ***P < 0.001, ****P < 0.0001.

To evaluate the protective effects of the aqueous extract of Artemisia argyi (AEAA) against LPS-induced acute lung injury (ALI), mice were assigned to a control group, an LPS model group, and three AEAA pretreatment groups (low, medium, and high dose). Histopathological examination showed that lungs in the control group exhibited intact alveolar architecture, thin septa, and minimal inflammatory infiltration. In contrast, LPS exposure induced pronounced lung injury characterized by marked septal thickening, extensive inflammatory infiltration, and partial alveolar collapse, confirming successful model establishment (**Figure 2B**).

In contrast, AEAA pretreatment was associated with a marked attenuation of LPS-induced histological injury. The high-dose group exhibited the greatest preservation of alveolar architecture and overall lung morphology, with tissue features closely resembling those of the control group (**Figure 2B**).

Consistent with the histological findings, the LPS group showed marked increases in the lung wet-to-dry (W/D) ratio—a key indicator of pulmonary edema—and in the lung injury score, reflecting pronounced fluid accumulation and tissue damage (**Figures 2C, D**). AEAA pretreatment significantly alleviated both measures, with the most notable improvement observed in the high-dose group (**Figures 2C, D**).

LPS exposure elicited a strong inflammatory response, marked by increased levels of IL-1β, IL-6, TNF-α, and myeloperoxidase (MPO) in both serum and bronchoalveolar lavage fluid (BALF) (**Figures 2E–M**). AEAA pretreatment reduced these pro-inflammatory mediators, with the most notable effects observed in the high-dose group. In addition, the LPS-induced decrease in IL-10 was reversed by high-dose AEAA. Overall, AEAA pretreatment was associated with reduced lung tissue injury, pulmonary edema, and inflammatory marker levels, with the strongest effects observed in the high-dose group.

### 3.3 AEAA pretreatment reshapes gut microbial diversity and community composition in LPS-challenged mice

**Figure 3:**
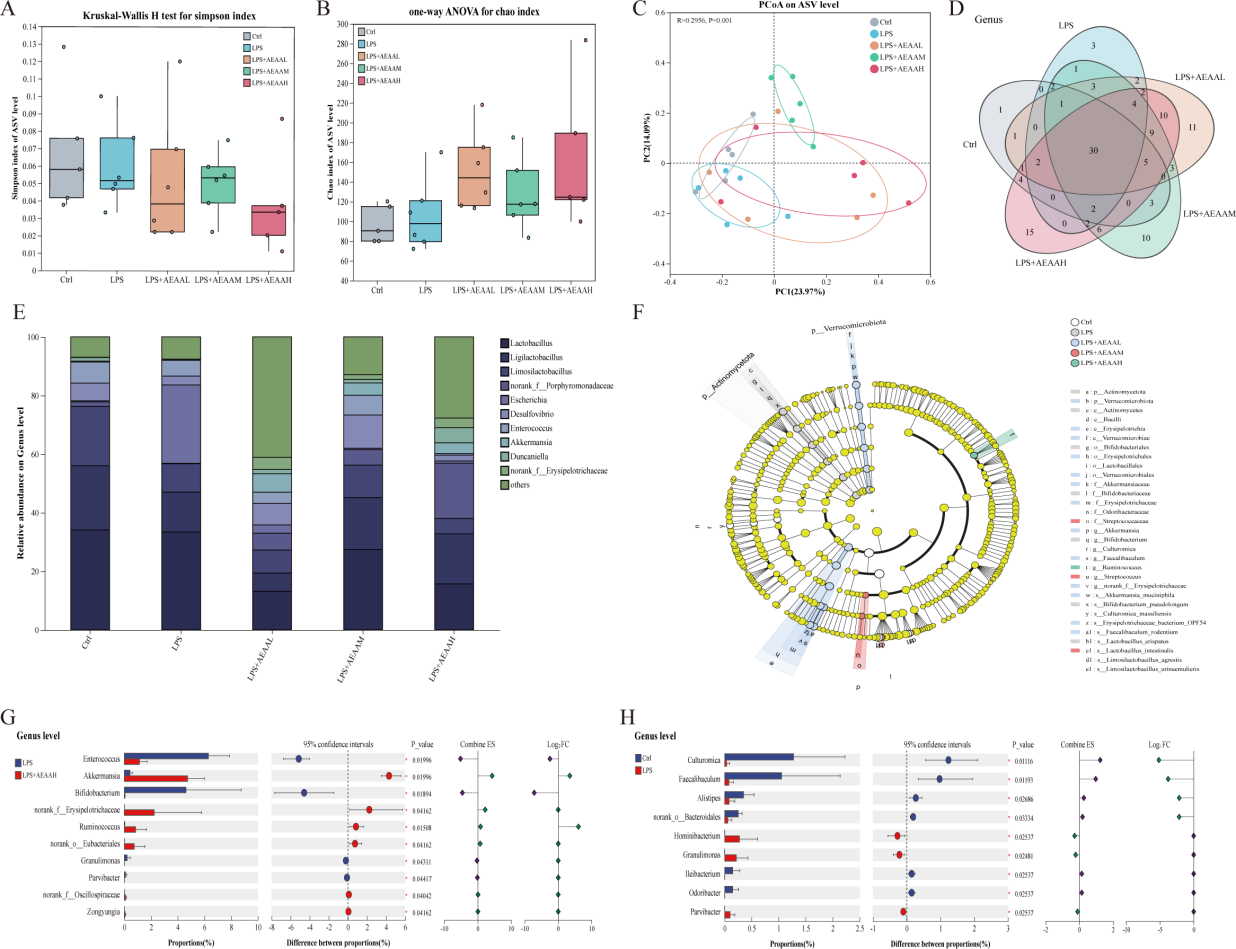
Gut microbiota diversity, community composition, and differential taxa analysis across experimental groups. **(A)** Simpson diversity index compared among groups using the Kruskal–Wallis test. **(B)** Chao1 richness index analyzed using one-way ANOVA. **(C)** Principal coordinate analysis (PCoA) based on ASV-level Bray–Curtis distances. **(D)** Venn diagram of shared and unique genera. **(E)** Relative abundance of dominant genera. LPS induces notable increases or decreases in genera such as Lactobacillus, Bifidobacterium, *Ruminococcus*, *Akkermansia*, and members of *Erysipelotrichaceae*, whereas AEAL/AEAM/AEAAH differentially modulate these taxa. **(F)** LEfSe cladogram. Linear discriminant analysis effect size (LEfSe) identifies key discriminative taxa enriched in each group, displayed in a cladogram from phylum to genus level. Highlighted nodes represent taxa with significant LDA scores, indicating group-specific microbial biomarkers. **(G, H)** Differential genus-level taxa analysis. Forest plots show effect size, log fold change (logFC), 95% confidence intervals, and P values for taxa significantly altered between: **(G)** LPS vs. LPS+AEAAH groups — highlighting genera such as *Enterococcus*, *Akkermansia, norank*, *f_Erysipelotrichaceae*, *Ruminococcus*, and *Parabacteroides*. **(H)** LPS vs. control groups — identifying shifts in *Clostridium*, *Faecalibaculum*, *Alloprevotella*, and others. Each panel includes proportional abundance comparisons and statistical estimation of differential taxa. Data presentation: box plots display medians and interquartile ranges; stacked bars represent relative abundance (%) at the genus level; LEfSe significance threshold LDA > 2.0.(n ≥ 5 mice per group for gut microbiota analysis).

To evaluate whether AEAA modulates LPS-induced gut dysbiosis, colonic microbiota composition was analyzed by 16S rRNA sequencing across all groups (**Figures 3A–H**). The control group exhibited stable α-diversity (Simpson and Chao1 indices), indicating preserved richness and evenness. LPS markedly reduced α-diversity, reflecting diminished microbial complexity and ecological stability (**Figures 3A, B**). AEAA pretreatment increased α-diversity in a dose-dependent manner, with the high-dose group showing the most pronounced improvement (**Figures 3A, B**).

β-diversity analysis revealed a clear separation between the control and LPS groups, indicating that LPS markedly altered the microbial community structure. AEAA pretreatment shifted the microbial profiles toward those of the control group in a dose-dependent manner, with the high-dose AEAA group clustering closest to controls in the PCoA plot (ANOSIM p = 0.001; **Figure 3C**). Venn analysis showed that although core taxa were shared across groups, AEAA-treated mice exhibited additional unique genera, and the number of these unique genera increased with the AEAA dose (**Figure 3D**).

Taxonomic profiling showed that LPS increased potentially pathogenic genera, including Escherichia and Enterococcus. In contrast, AEAA elevated beneficial genera, particularly Akkermansia, in a dose-dependent manner (**Figure 3E**). LEfSe and differential abundance analyses further revealed that inflammation-associated taxa such as Enterococcus, Ruminococcus, and Erysipelotrichaceae were enriched in the LPS group, whereas AEAA reduced these taxa and increased beneficial bacteria, notably Akkermansia and Bifidobacterium, with the strongest effects observed in the high-dose group (**Figures 3F, G**). Collectively, these findings indicate that AEAA pretreatment was associated with partial normalization of LPS-induced alterations in gut microbial diversity and community composition, with higher doses producing profiles more similar to those of the control group.

### 3.4 AEAA pretreatment alters LPS-induced intestinal metabolic profiles

**Figure 4:**
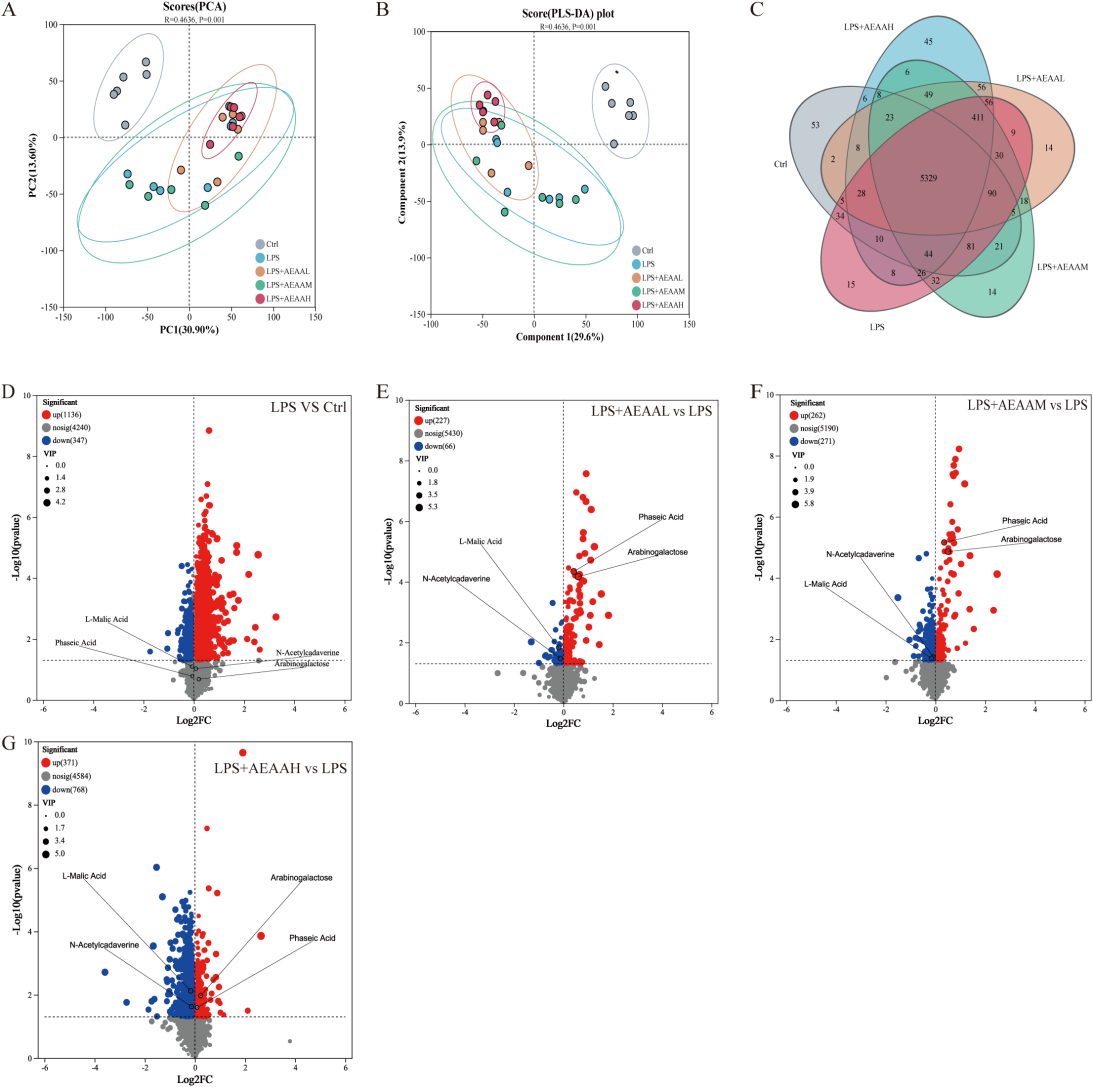
Multivariate analysis of colonic metabolic profiles across experimental groups. **(A)** Principal component analysis (PCA). **(B)** Partial least-squares discriminant analysis (PLS-DA).Score plot further demonstrating group discrimination with robust model performance (R² and Q² values shown), suggesting reliable metabolic differences. **(C)** Venn diagram illustrating the overlapping and unique differential metabolites identified across the four comparisons. Shared metabolites indicate common metabolic disturbances, while group-specific metabolites reflect treatment-dependent metabolic shifts. **(D-G)** Volcano plot comparing different groups. **(D)** LPS vs. Ctrl; **(E)** LPS + AEAAL vs. LPS; **(F)** LPS + AEAAM vs. LPS; **(G)** LPS + AEAAH vs. LPS. Highlighted metabolites indicate significant upregulation **(red)** and downregulation (blue). Following AEAA supplementation, alterations occurred in key metabolites, including decreased levels of L-malic acid and N-acetylcysteamine, alongside increased levels of arabinogalactose and phasic acid. Differential metabolites were identified using *VIP > 1.5* and *P < 0.05*. Red dots represent significantly upregulated metabolites, blue dots represent significantly downregulated metabolites, and gray dots indicate non-significant changes.(n ≥ 5 mice per group for colonic metabolomics analysis).

Untargeted metabolomic analysis of colonic contents was conducted to determine whether AEAA modulates intestinal metabolic disturbances during LPS-induced ALI. PCA and OPLS-DA revealed distinct metabolic profiles among the control, LPS, and AEAA-treated groups, with AEAA dose-dependently shifting the metabolomic landscape away from the LPS group (**Figures 4A, B**). Venn analysis highlighted both shared and unique metabolites across groups (**Figure 4C**), and hierarchical clustering confirmed broad metabolic reprogramming following AEAA pretreatment.

VIP-based feature selection identified key metabolites distinguishing the groups, including arabinogalactose, phaseic acid, L-malic acid, and N-acetylcadaverine. LPS notably altered the levels of these metabolites compared with controls (**Figure 4D**). AEAA pretreatment modulated several LPS-affected metabolites: the medium dose increased arabinogalactose and phaseic acid while reducing L-malic acid and N-acetylcadaverine (**Figure 4E**), and the high dose produced similar but more pronounced effects relative to LPS alone (**Figure 4F**). Overall, AEAA pretreatment was associated with shifts in multiple LPS-altered intestinal metabolites toward levels closer to those observed in the control group.

### 3.5 AEAA pretreatment reshapes systemic metabolic profiles in response to LPS challenge

**Figure 5:**
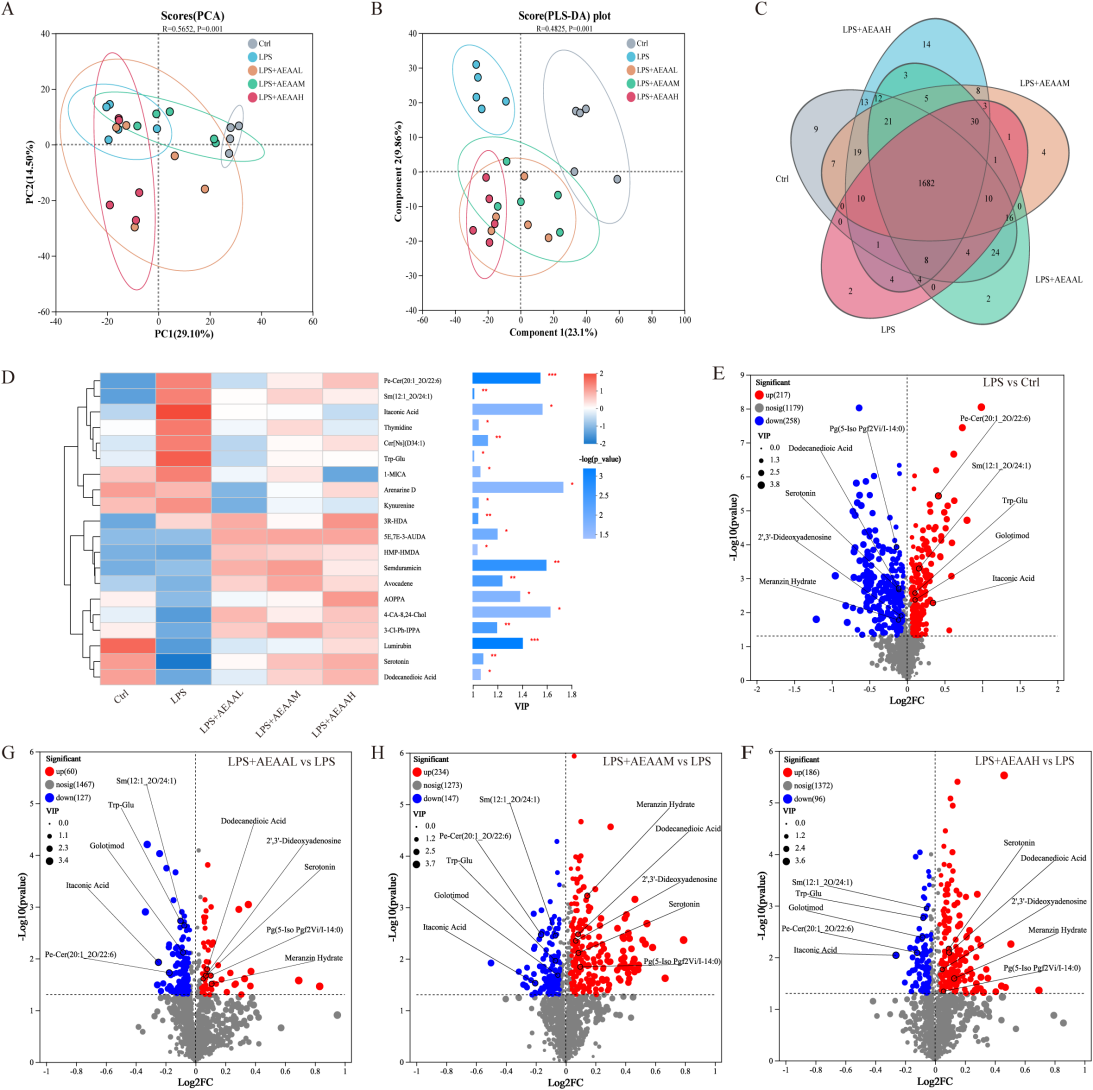
Serum metabolic profile alterations across experimental groups. **(A)** PCA score plot. Distinct clustering patterns emerged across different groups, revealing overall metabolic differences. Ellipses denote 95% confidence intervals. Ellipses represent 95% confidence regions. **(B)** PLS-DA score plot. Partial least squares discriminant analysis (PLS-DA) demonstrating clear group separation, confirming significant metabolic alterations following LPS induction and treatments with AEAAM or AEAAH. **(C)** Venn diagram of detected metabolites. Overlap of annotated serum metabolites across four groups, illustrating shared and unique metabolites in different samples. **(D)** Heatmap and VIP scores of differential metabolites. Left: Hierarchical clustering heatmap presenting the relative abundance of significantly altered metabolites. Right: Variable importance in projection (VIP) scores identifying key metabolites contributing to group discrimination, including P-Cresol, Serotonin, Tryptophan, Deoxycholic acid, Inosine, and others. **(E-H)** Volcano plot (LPS vs Ctrl).Differential metabolites between the LPS and Ctrl groups. Red dots indicate significantly upregulated metabolites; blue dots represent significantly downregulated metabolites (criteria: VIP > 1.5, *P < 0.05*). (n ≥ 5 mice per group for serum metabolomics analysis). *P < 0.05, **P < 0.01, ***P < 0.001.

Untargeted serum metabolomics was performed to assess systemic metabolic disturbances. PCA and PLS-DA revealed clear distinctions among the control, LPS, and AEAA-treated groups, confirming that LPS induced extensive metabolic disruption, while AEAA pretreatment shifted metabolic profiles toward those of the controls (**Figures 5A, B**). Medium- and high-dose AEAA groups clustered more closely with controls than the low-dose group, with the high-dose group showing the strongest overlap (**Figure 5B**). Venn analysis identified both shared and unique metabolites across groups (**Figure 5C**), and VIP-based clustering highlighted AEAA-responsive metabolites, including dodecanedioic acid, 2′,3′-dideoxyadenosine, serotonin, Meranzin hydrate, and itaconic acid (**Figure 5D**).

Volcano plot analysis revealed substantial LPS-induced metabolic alterations compared with controls (**Figure 5E**). Relative to the LPS group, AEAA pretreatment elevated metabolites such as dodecanedioic acid, 2′,3′-dideoxyadenosine, and serotonin, while reducing itaconic acid, Golotimod, and Pe-Cer(20:1_2O/22:6), with the most pronounced effects observed at the high dose (**Figures 5F–H**). These findings suggest a dose-dependent association between AEAA pretreatment and the attenuation of LPS-induced systemic metabolic disturbances.

### 3.6 AEAA pretreatment reshapes inflammatory gene expression and signaling profiles in the lung

**Figure 6:**
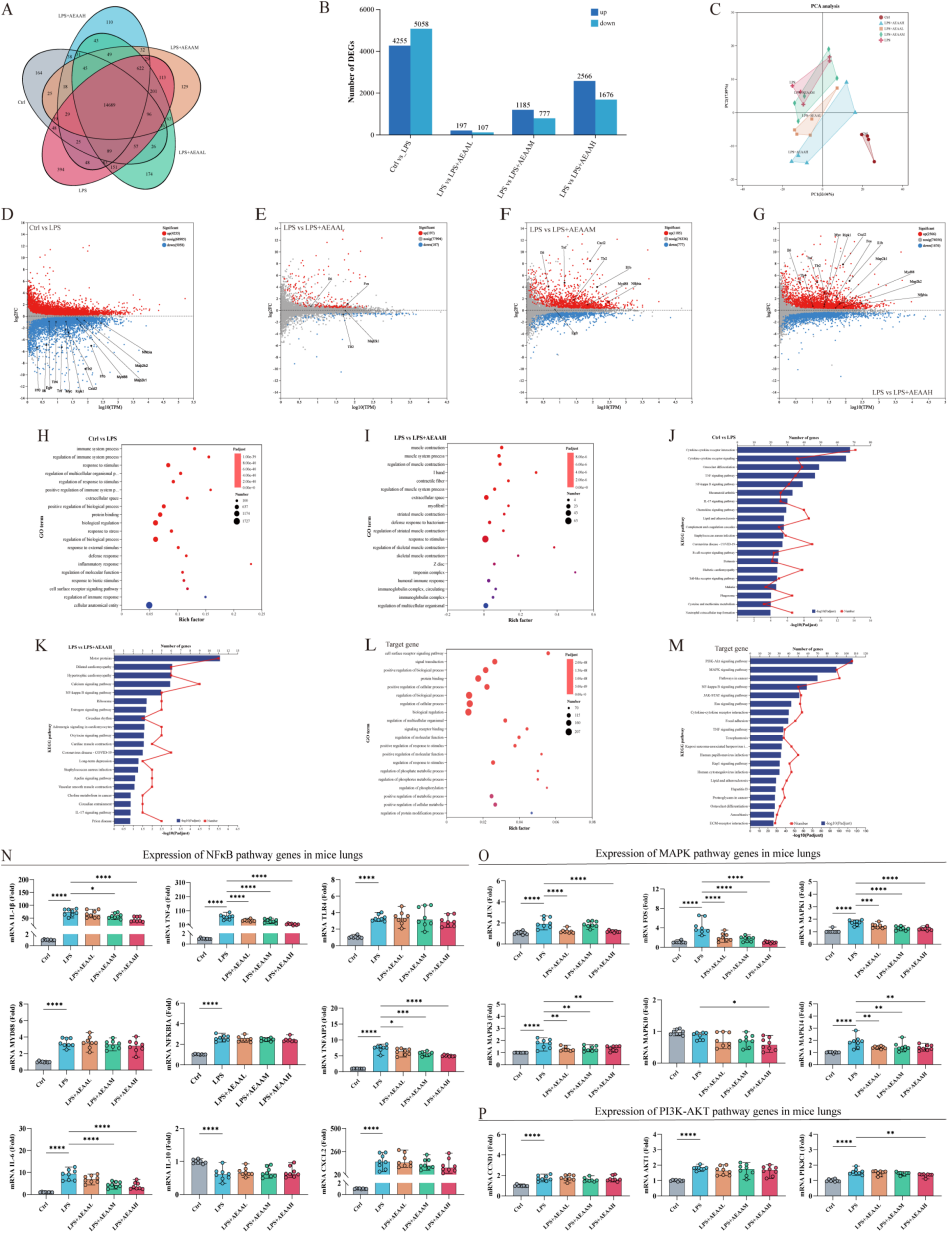
Transcriptomic profiling and pathway-associated gene expression changes in ALI mouse lungs. **(A)** Venn diagram showing the overlap of differentially expressed genes (DEGs). **(B)** Numbers of upregulated and downregulated DEGs identified in each comparison. **(C)** Principal component analysis (PCA) illustrating distinct clustering of transcriptomic profiles across treatment groups. **(D–G)** Volcano plots depicting significantly upregulated (red) and downregulated (blue). DEGs **(D)** LPS vs Ctrl, **(E)** LPS vs LPS + AEAAL, **(F)** LPS vs LPS + AEAAM; **(G)** LPS vs LPS + AEAAH. Representative DEGs are labeled. **(H, I, L)** GO biological process enrichment analysis of DEGs. **(H)** Ctrl vs LPS; **(I)** LPS vs LPS + AEAAH. **(L)** Target genes. **(J, K, M)** KEGG pathway enrichment analysis. **(J)** Ctrl vs LPS; **(K)** LPS vs LPS + AEAAH; **(M)** Target genes. Bubble size represents gene count; color denotes significance. **(N)** Expression of key NF-κB pathway genes (e.g., *Il6*, *Il1β*, *Tnf*, *Cxcl2*, *Nfkbia*, *Tlr4*, *Myd88*.) in mouse lung tissues. **(O)** Expression of MAPK pathway genes (e.g., *Mapk1, Mapk3, Mapk10, Mapk14, Fos, Jun*). **(P)** Expression of PI3K–AKT pathway genes (e.g., *CCND1*, *Akt1*, *PI3KCA*). **(A)** Data are presented as mean ± SEM. Statistical significance: *P < 0.05, **P < 0.01, ***P < 0.001, ****P < 0.0001. (n ≥ 5 mice per group for lung Transcriptomic analysis and n = 8 mice per group for qPCR).

To characterize the transcriptional changes induced by LPS and the protective effects of AEAA, we performed lung RNA-seq across experimental groups (**Figure 6A**). LPS triggered extensive transcriptomic reprogramming, evidenced by numerous differentially expressed genes (DEGs) and clear separation from controls in global analyses (**Figure 6B**). AEAA pretreatment reduced both the number and amplitude of DEGs and shifted the transcriptomic profile toward that of the control group in PCA, indicating partial restoration of transcriptional homeostasis.

Pairwise volcano plots revealed robust induction of pro-inflammatory cytokines and chemokines following LPS stimulation (**Figure 6D**), whereas AEAA pretreatment markedly attenuated the expression of these LPS-driven inflammatory genes (**Figures 6E–G**). Functional enrichment analysis showed that LPS-induced DEGs were primarily associated with cytokine signaling, chemokine-mediated cell recruitment, and innate immune activation pathways (**Figure 6H**), and these enrichments were diminished after AEAA pretreatment (**Figure 6I**). Canonical pathway analysis revealed enrichment of gene sets related to NF-κB, MAPK, Toll-like receptor, and PI3K–AKT–associated signaling pathways following LPS challenge, with comparatively reduced enrichment observed in AEAA-pretreated groups. (**Figures 6J–M**).

qPCR validation showed that key inflammatory mediators and pathway markers—including NF-κB–related cytokines (Il1β, Tnfα, Il6), MAPK-associated genes (Fos, Jun, Mapk1), and PI3K–AKT components (Ccnd1, Akt1, PIK3C1)—were elevated after LPS stimulation but markedly decreased following AEAA treatment (**Figures 6N–P**). Overall, AEAA pretreatment was associated with reduced LPS-induced inflammatory gene expression and attenuated transcriptional signatures associated with multiple inflammatory signaling pathways in the lung.

### 3.7 Integrated multi-omics analyses reveal coordinated interactions among microbial, metabolic, and pulmonary inflammatory features

**Figure 7:**
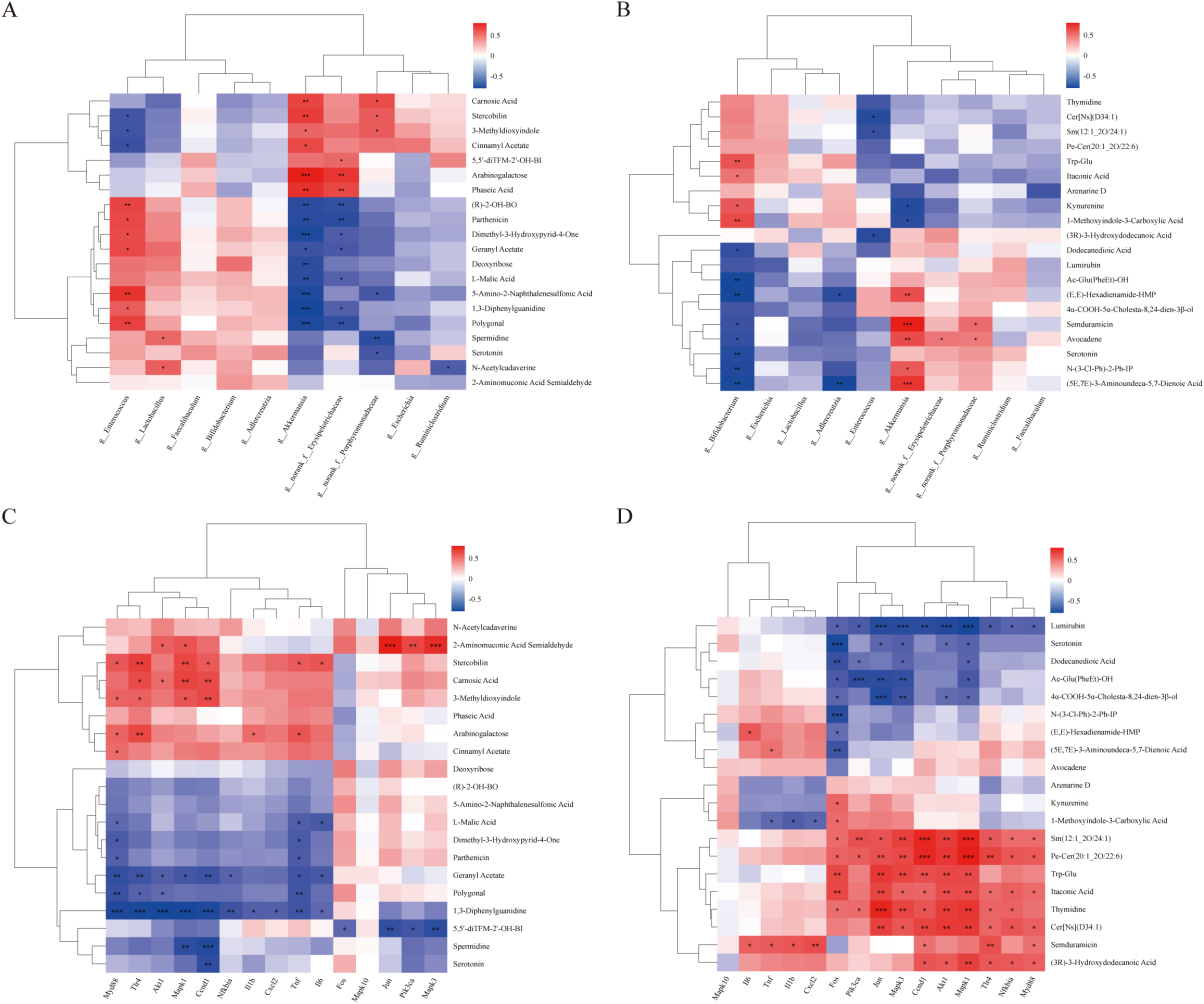
Integrated multi-omics analyses reveal coordinated associations among gut microbiota, metabolic features, and pulmonary inflammatory gene expression. **(A)** Spearman correlation analysis between differentially abundant gut microbial genera and colonic metabolites. **(B)** Spearman correlation analysis between gut microbial genera and serum metabolites. **(C)** Correlation analysis between colonic metabolites and lung transcriptomic features. **(D)** Correlation analysis between serum metabolites and lung transcriptomic features. Correlation matrices display representative differential features from each omics layer, with color intensity indicating the strength and direction of correlations. Only selected top correlations are shown to enhance clarity, while complete correlation matrices are provided in the **Supplementary Material**. These integrated analyses were conducted in an exploratory manner to characterize treatment-associated cross-omics associations rather than to infer direct causal relationships. n = 5 mice per group for microbiome, metabolomics and transcriptomics analyses. *P < 0.05, **P < 0.01, ***P < 0.001.

To examine coordinated interactions among gut microbiota, metabolic shifts, and pulmonary transcriptional responses, we conducted an integrated multi-omics correlation analysis to explore coordinated associations among gut microbiota, metabolic features, and pulmonary inflammatory gene expression (**Figure 7**). Spearman correlation matrices with hierarchical clustering revealed distinct interaction patterns among microbial taxa, metabolites, and lung inflammatory gene expression, underscoring gut–lung axis–related associations shaped by AEAA pretreatment.

Correlation analysis (**Figure 7A**) revealed two distinct clusters. AEAA-enriched beneficial genera, including Akkermansia and *Lactobacillus*, were linked to polysaccharide- and homeostasis-related metabolites such as arabinogalactose, phaseic acid, serotonin, and spermidine. In contrast, LPS-associated genera such as Enterococcus, Escherichia, and Ruminococcus were associated with stress- and inflammation-related metabolites, including N-acetylcadaverine and L-malic acid. These patterns suggest that AEAA-associated microbial shifts co-occurred with a more normalized intestinal metabolic profile.

Consistent trends emerged in the associations between microbes and serum metabolites (**Figure 7B**). AEAA-enriched taxa showed positive correlations with systemic metabolites involved in metabolic homeostasis and immune regulation, such as serotonin and dodecanedioic acid, and negative correlations with inflammatory metabolites including itaconic acid and kynurenine. In contrast, inflammation-associated microbes demonstrated the opposite pattern, underscoring the coordinated associations between AEAA-associated shifts in gut microbiota and systemic metabolic features.

Integration of colonic metabolomics with lung transcriptomics revealed coordinated metabolite–gene patterns. Anti-inflammatory metabolites (arabinogalactose, phaseic acid, serotonin) were inversely correlated with key pulmonary inflammatory genes (Il1β, Il6, Tnfα, Fos, Jun), whereas stress-associated metabolites (N-acetylcadaverine, L-malic acid) showed positive correlations. Clustering analyses grouped metabolites and genes into modules that reflected either inflammatory activation or metabolic homeostasis.

Serum metabolite–RNA-seq correlation analysis (**Figure 7D**) revealed that pro-inflammatory metabolites, including itaconic acid, ceramides, and sphingomyelins, were positively associated with lung genes involved in the NF-κB, MAPK, and PI3K–AKT pathways. In contrast, AEAA-elevated metabolites such as serotonin and dodecanedioic acid showed inverse correlations with these inflammatory gene sets. These results highlight coordinated associations among gut microbes, metabolites, and pulmonary inflammatory gene expression.

## 4 Discussion

Acute lung injury (ALI) remains a major clinical challenge characterized by high mortality, limited targeted treatments, and significant global healthcare burden (2, 3, 45). In this study, we show that pretreatment with an aqueous extract of Artemisia argyi (AEAA) is associated with marked attenuation of LPS-induced ALI. Our results show that AEAA pretreatment was associated with marked attenuation of lung injury, as reflected by improved histopathology, reduced inflammatory markers, and coordinated alterations in gut microbiota composition, metabolic profiles, and pulmonary inflammatory gene expression. Together, these findings suggest that AEAA treatment is accompanied by multi-level biological changes that are consistent with emerging concepts of gut–lung axis involvement in systemic inflammation (46, 47).

Importantly, although coordinated alterations were observed across the gut microbiota, metabolic profiles, and pulmonary inflammatory signaling, the present study does not establish a causal gut–lung axis mechanism. Rather, the findings provide an integrated, hypothesis-generating framework that aligns with—but does not confirm—gut–lung axis–related regulation. Establishing causality will require targeted microbiota manipulation and mechanistic validation in future studies.

At the microbial level, AEAA pretreatment was associated with partial normalization of LPS-induced dysbiosis and a shift in microbial community structure toward profiles more similar to those of control animals. Inflammation-associated taxa enriched following LPS challenge, such as *Enterococcus* and *Escherichia*, have been linked to impaired barrier function and heightened systemic inflammation. In contrast, AEAA-treated mice exhibited increased relative abundance of beneficial genera, particularly *Akkermansia muciniphila* and *Lactobacillus* (48–51), which are known to support epithelial integrity, maintain mucus-layer homeostasis, and contribute to immune tolerance through metabolite production (52–55). While these observations are consistent with the notion that a healthier microbial ecosystem may favor improved inflammatory outcomes, the present data do not demonstrate that microbial alterations are required for AEAA-mediated lung protection. Nonetheless, these findings support growing interest in microbiota-oriented strategies for modulating inflammatory lung diseases and suggest that AEAA may represent a botanical approach that avoids the collateral effects associated with broad-spectrum antimicrobial interventions.

Metabolomic profiling further revealed that AEAA pretreatment was associated with substantial modulation of intestinal metabolic disturbances induced by LPS. In colonic contents, AEAA increased levels of arabinogalactose and phaseic acid, metabolites linked to polysaccharide metabolism and reported anti-inflammatory properties (56, 57). These changes are consistent with prior evidence showing that fermentation of complex carbohydrates and fiber-derived metabolites can enhance barrier integrity and support balanced immune responses (58). Conversely, AEAA reduced metabolites such as N-acetylcadaverine and L-malate, which have been associated with proteolytic fermentation and stress-related metabolic states (59). Collectively, these findings suggest that AEAA pretreatment is associated with an intestinal metabolic milieu that may be less permissive to mucosal stress and inflammatory amplification.

Beyond the gut, AEAA pretreatment was also associated with systemic metabolic reprogramming. Serum metabolomics demonstrated reduced levels of itaconate, a metabolite linked to inflammatory macrophage activation and innate immune metabolic adaptation (60–62). AEAA further partially normalized lipid metabolites, including ceramides and sphingomyelins, which have been implicated in macrophage activation, endothelial dysfunction, and pro-inflammatory signaling in ALI (63, 64). In addition, AEAA increased circulating serotonin levels, consistent with established links among gut microbiota, serotonin biosynthesis, and immune modulation (65, 66). These systemic metabolic associations provide a plausible context for the attenuated inflammatory responses observed but should be interpreted as correlative rather than mechanistic.

At the pulmonary level, transcriptomic and qPCR analyses indicated that AEAA pretreatment was associated with reduced expression of LPS-induced pro-inflammatory genes and diminished enrichment of gene sets related to NF-κB, ERK/MAPK, and PI3K–AKT associated signaling pathways (67, 68). The increased expression of negative regulators such as *Tnfaip3* and *Nfkbia* in AEAA-treated lungs suggests a potential reinforcement of endogenous feedback mechanisms that promote inflammation resolution (69, 70).

A key strength of this study is the implementation of an integrated multi-omics framework, which enabled systems-level characterization of coordinated alterations across microbial, metabolic, and pulmonary inflammatory dimensions. Correlation-based integration (e.g., SparCC/MINT (71)) revealed significant associations linking specific microbial taxa (e.g., *Lactobacillus*, *Akkermansia*) with host-derived metabolites and pulmonary inflammatory gene signatures. While inherently correlative, these multi-layered analyses establish a mechanistically informed scaffold for deciphering gut–lung axis crosstalk (72, 73) and generate prioritized, testable hypotheses regarding microbiota-metabolite-immune interactions in inflammatory lung pathologies. However, because inflammatory pathway activation is predominantly regulated at the post-translational level, these transcriptional changes should be interpreted cautiously as associative rather than indicative of altered pathway activity. Protein-level validation (e.g., via immunoblotting or activity assays) and functional studies are therefore necessary to determine whether AEAA directly or indirectly modulates these signaling cascades, and to elucidate whether the observed effects stem from systemic AEAA exposure, microbiota-mediated metabolic shifts, or a combination of both.

Several limitations warrant acknowledgment. First, the prophylactic administration paradigm demonstrates AEAA’s preventive efficacy against LPS-induced acute lung injury (ALI) but does not fully recapitulate clinical scenarios involving established disease. Second, while multi-omics integration revealed coordinated alterations across gut microbiota, metabolome, and pulmonary inflammation, causal inference requires validation through targeted approaches such as microbiota depletion, fecal microbiota transplantation, or metabolite supplementation. Third, AEAA constitutes a complex botanical formulation; identification of bioactive constituents and rigorous extract standardization are essential for reproducibility and translational advancement. Addressing these constraints will be pivotal for refining the mechanistic understanding of AEAA’s actions.

In conclusion, this study demonstrates that prophylactic administration of an aqueous extract of *Artemisia argyi* (AEAA) significantly attenuates LPS-induced ALI, accompanied by concordant modulation of pulmonary histopathology, inflammatory cytokine profiles, gut microbial ecology, systemic metabolome, and lung transcriptome. Although direct causal evidence for a gut–lung axis mechanism remains to be established, the multi-layered dataset provides a mechanistically informed, hypothesis-generating framework consistent with emerging gut–lung biology. Collectively, these findings position AEAA as a promising multi-target botanical candidate and underscore the power of integrated multi-omics strategies to dissect cross-organ communication in complex inflammatory pathologies—offering a roadmap for future mechanistic validation and therapeutic development.

## Data Availability

The data presented in the study are deposited in the MetaboLights repository, accession number MTBLS14155 and MTBLS14160, and the GEO, Accession number: PRJNA1442991.
