# Supplementary figures and images for: Aqueous Artemisia argyi extract mitigates acute lung injury in association with coordinated alterations in gut microbiota, metabolic homeostasis, and pulmonary inflammatory gene expression
Source: Front Immunol. 2026 Apr 2;17:1770675. doi: 10.3389/fimmu.2026.1770675 (PMC13082973; doi:10.3389/fimmu.2026.1770675)

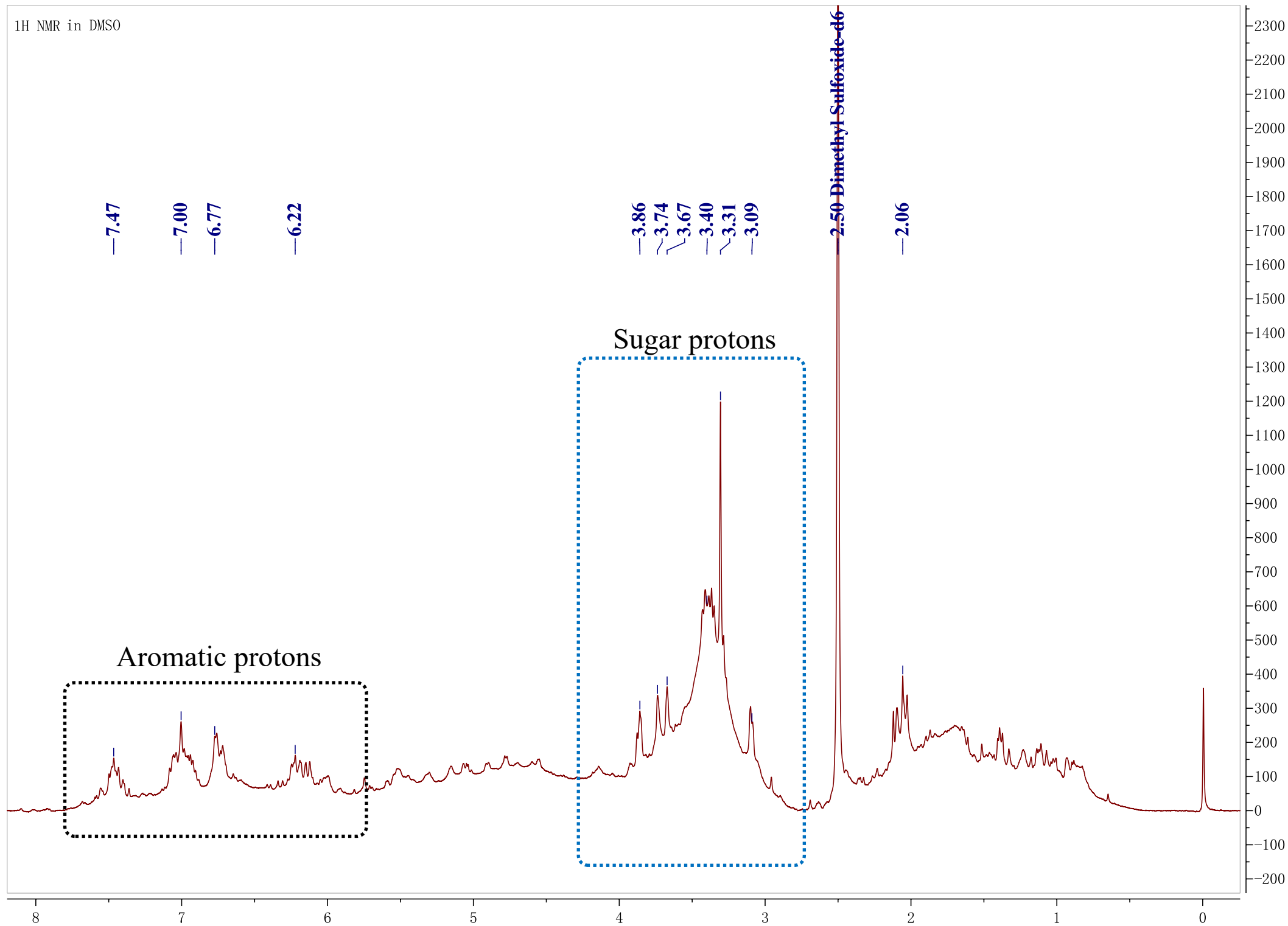

Supplement: Supplementary file 1 [file DataSheet1.zip › Data/Figure/fig1/A.pdf]

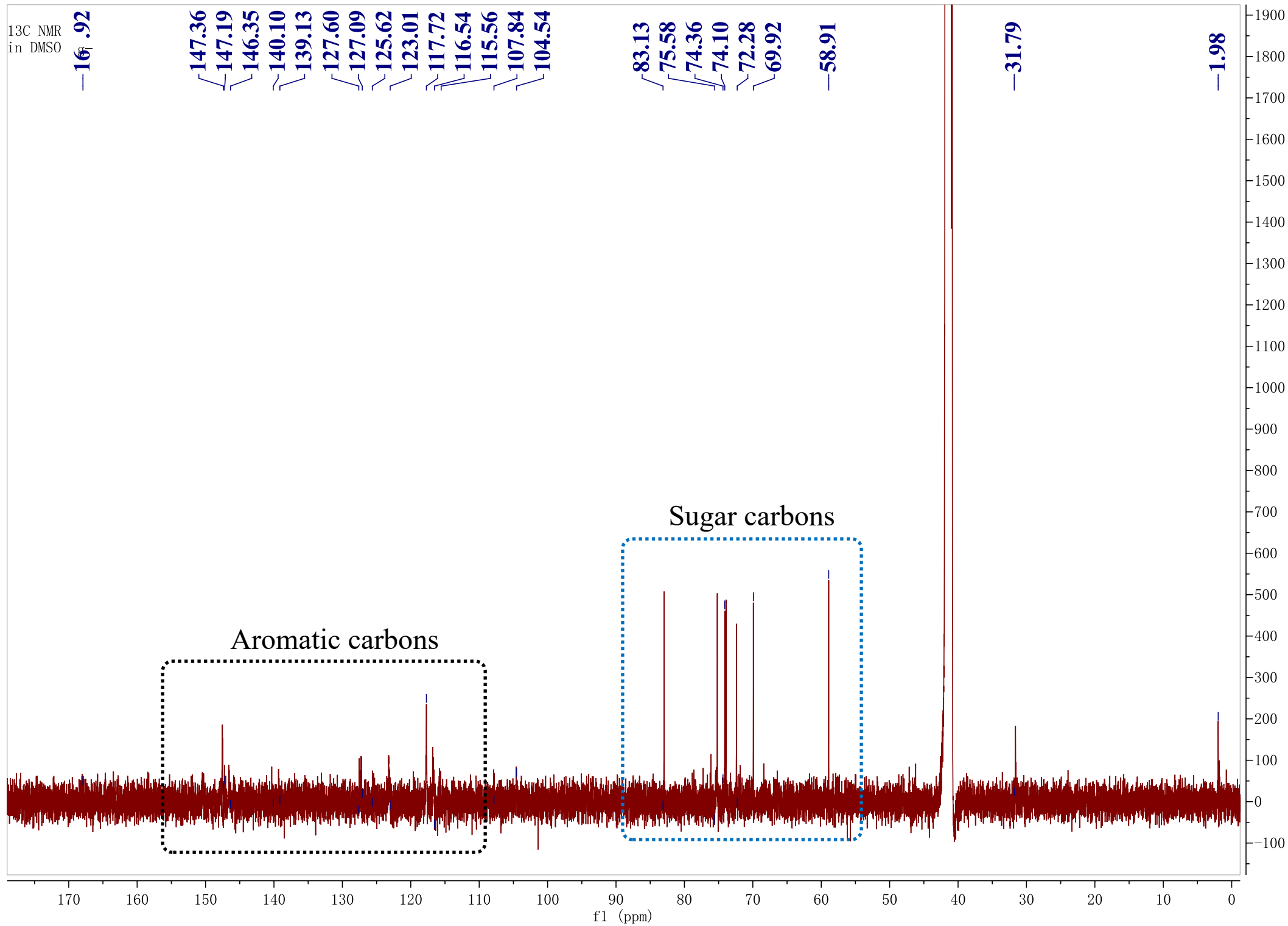

Supplement: Supplementary file 1 [file DataSheet1.zip › Data/Figure/fig1/B.pdf]

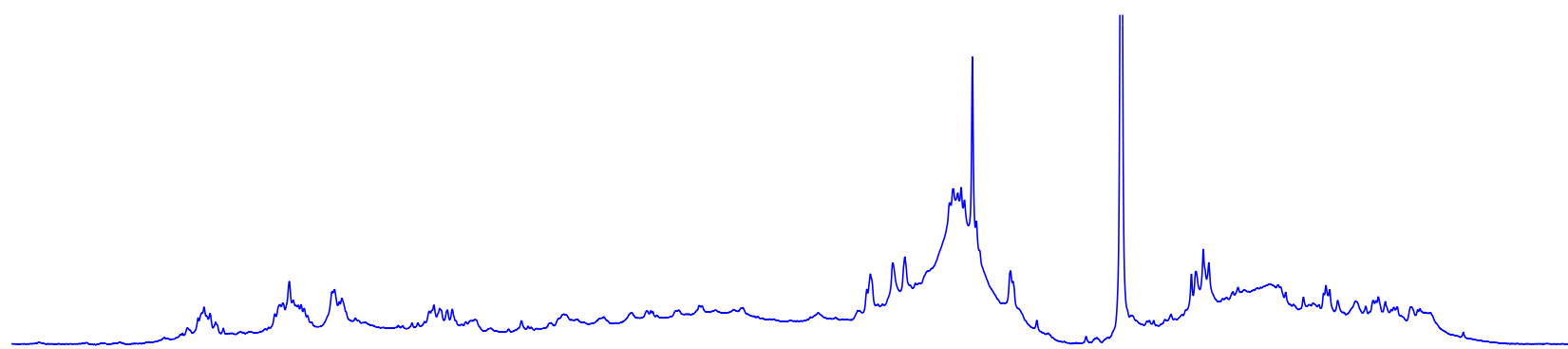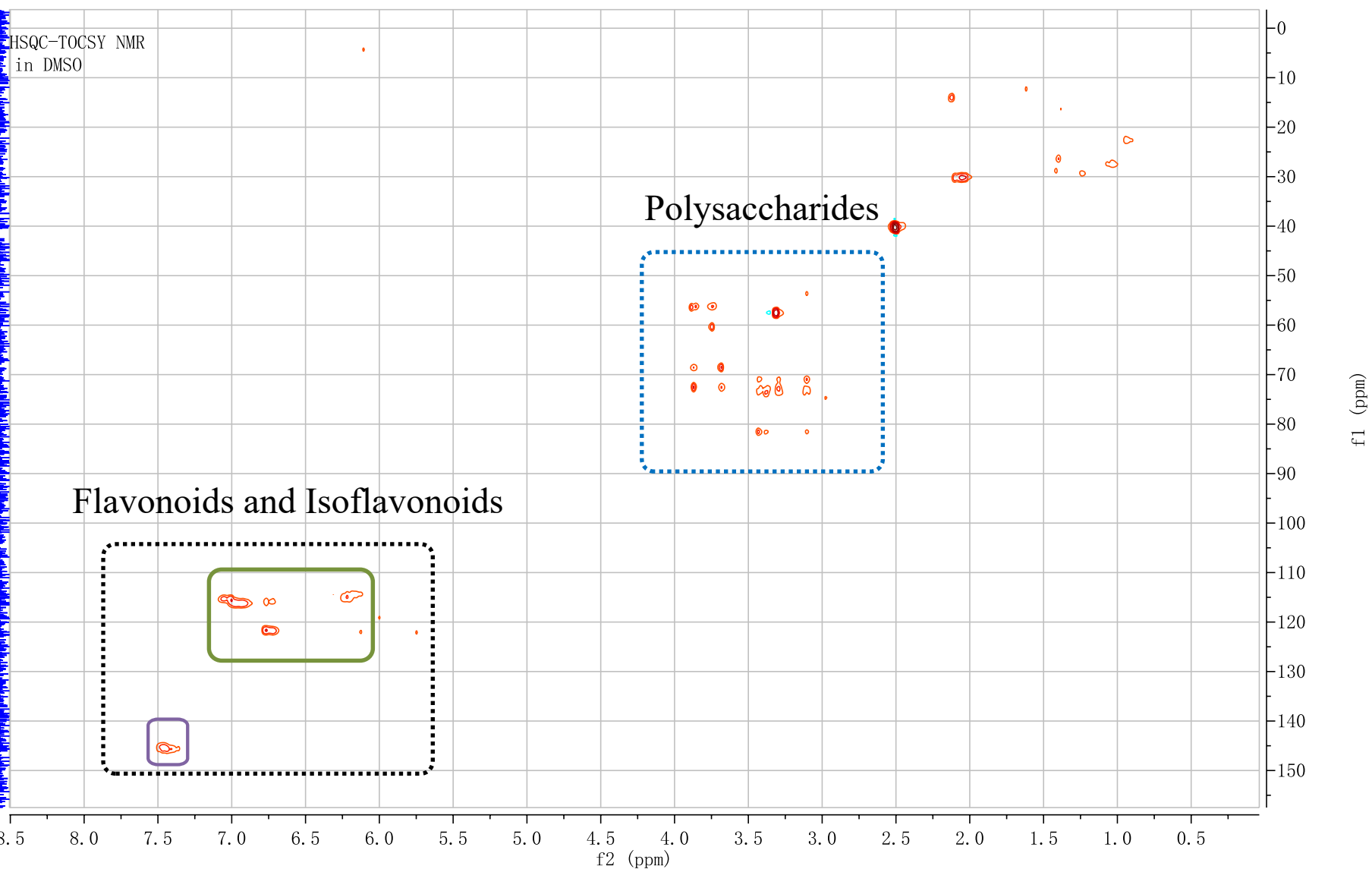

Supplement: Supplementary file 1 [file DataSheet1.zip › Data/Figure/fig1/C.pdf]

|      | Ctrl                                                                              | LPS                                                                                | LPS+AEAAL                                                                           | LPS+AEAAM                                                                           | LPS+AEAAH                                                                           |
|------|-----------------------------------------------------------------------------------|------------------------------------------------------------------------------------|-------------------------------------------------------------------------------------|-------------------------------------------------------------------------------------|-------------------------------------------------------------------------------------|
| AEAA | -                                                                                 | -                                                                                  | 0.5mg/mL                                                                            | 1mg/mL                                                                              | 1.5mg/mL                                                                            |
| LPS  | -                                                                                 | 5mg/kg                                                                             | 5mg/kg                                                                              | 5mg/kg                                                                              | 5mg/kg                                                                              |
| 20×  | 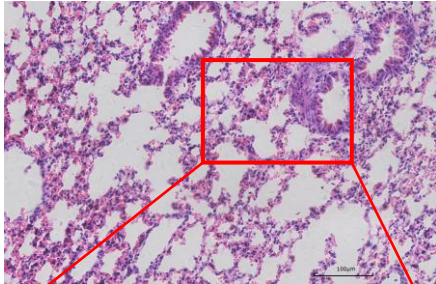 | 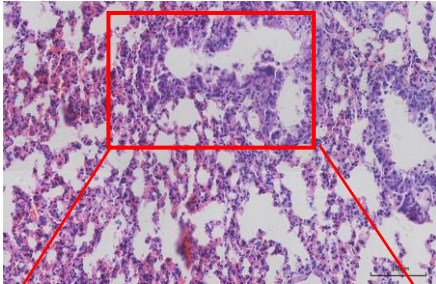 | 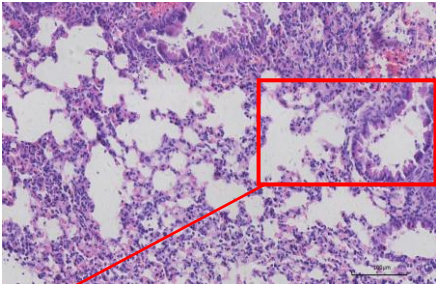 | 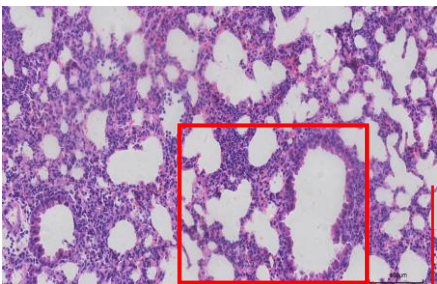 | 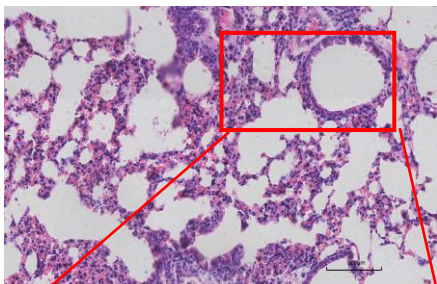 |
| 40×  | 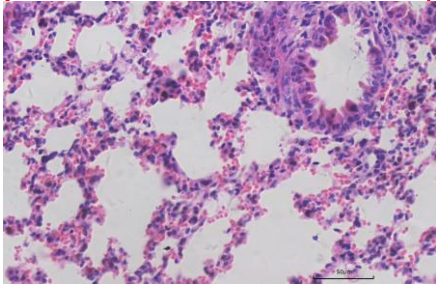 | 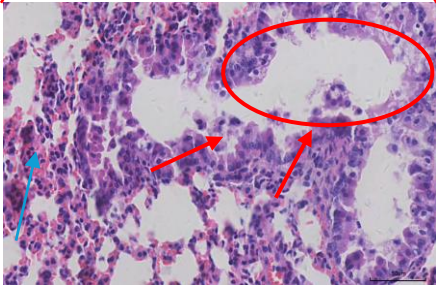 | 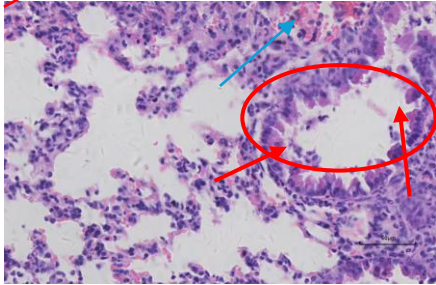 | 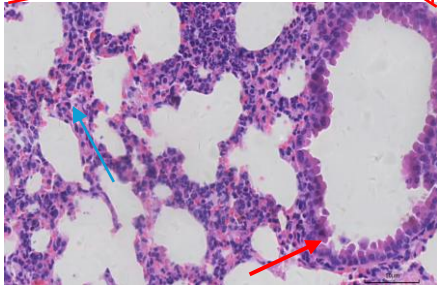 | 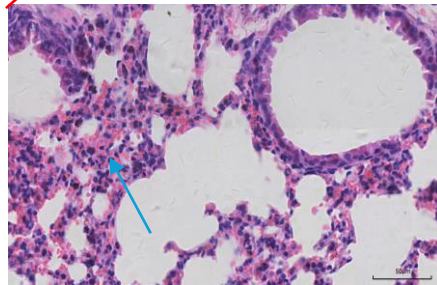 |

Supplement: Supplementary file 1 [file DataSheet1.zip › Data/Figure/fig2/HE.pdf]

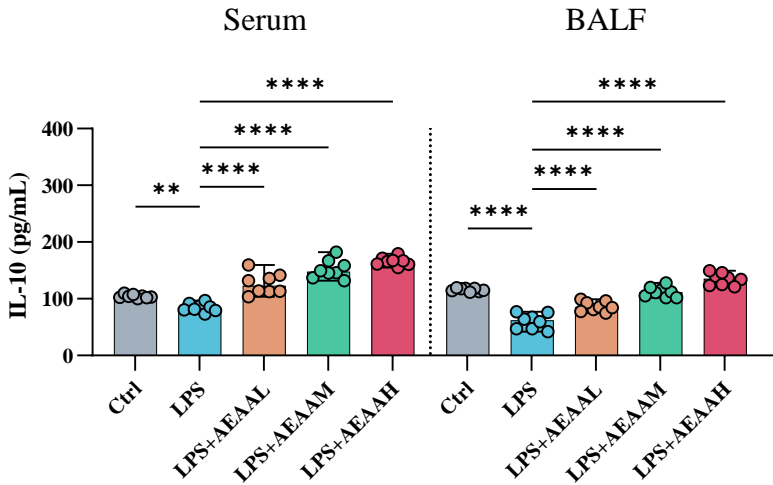

Supplement: Supplementary file 1 [file DataSheet1.zip › Data/Figure/fig2/IL-10.pdf]

Serum

BALF

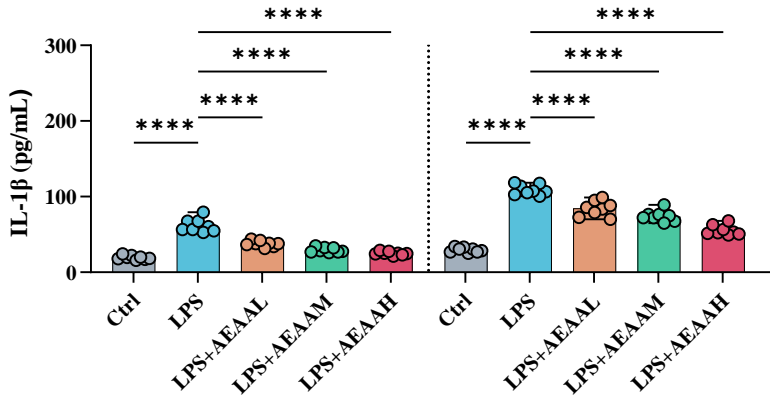

Supplement: Supplementary file 1 [file DataSheet1.zip › Data/Figure/fig2/IL-1β.pdf]

Serum

BALF

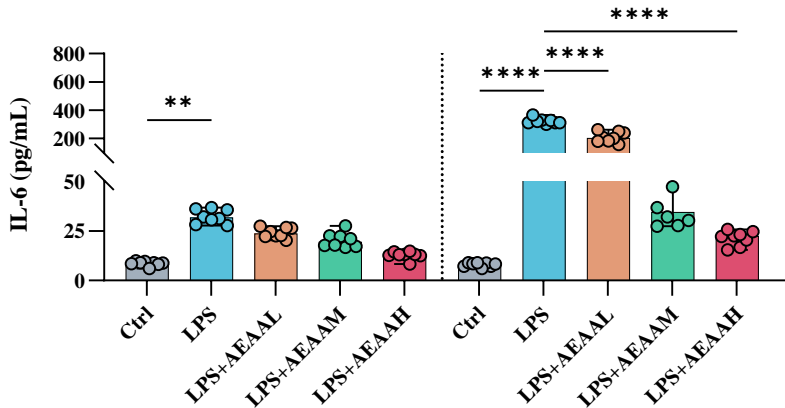

Supplement: Supplementary file 1 [file DataSheet1.zip › Data/Figure/fig2/IL-6.pdf]

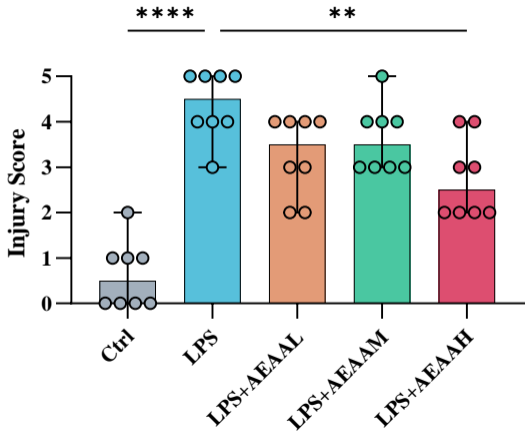

Supplement: Supplementary file 1 [file DataSheet1.zip › Data/Figure/fig2/Injury score.pdf]

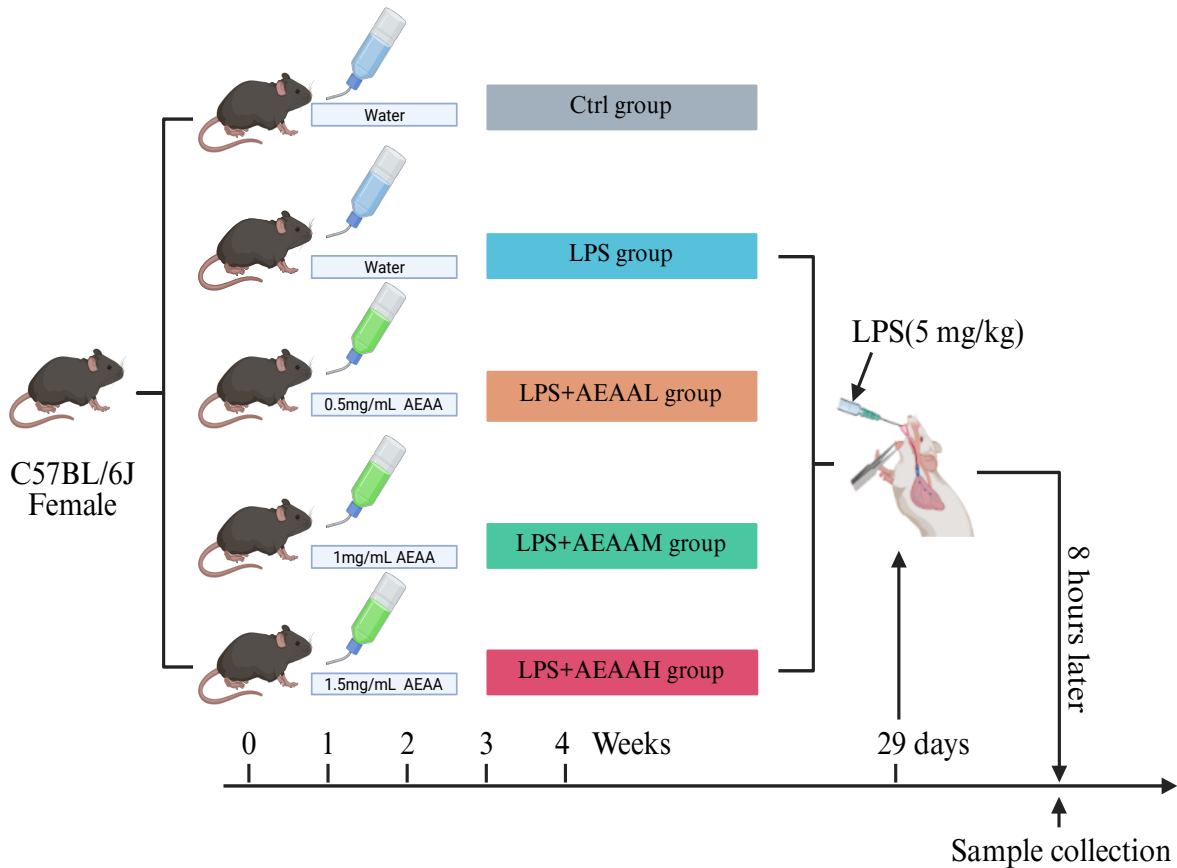

Supplement: Supplementary file 1 [file DataSheet1.zip › Data/Figure/fig2/Protocol.pdf]

Serum

BALF

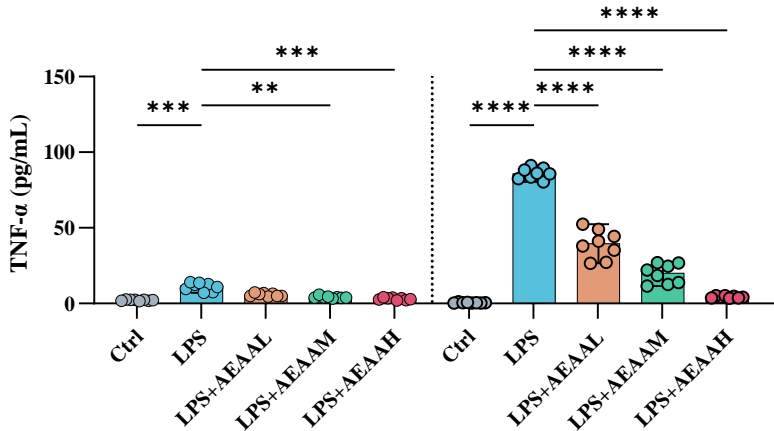

Supplement: Supplementary file 1 [file DataSheet1.zip › Data/Figure/fig2/TNFα.pdf]

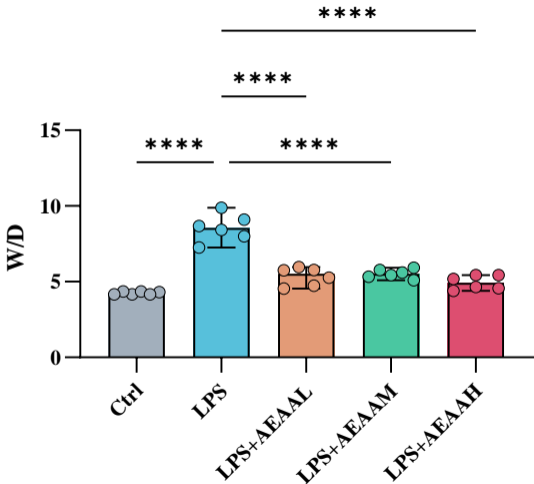

Supplement: Supplementary file 1 [file DataSheet1.zip › Data/Figure/fig2/W_D.pdf]

## Kruskal-Wallis H test for simpson index

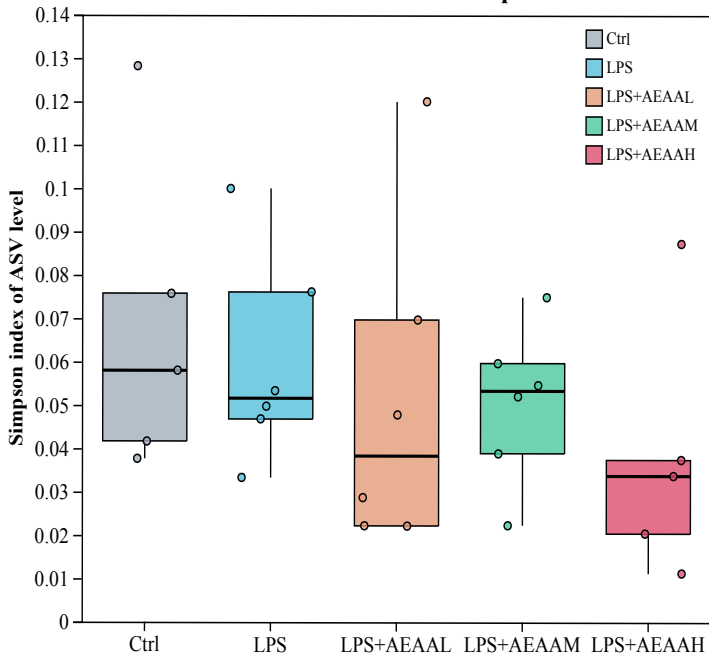

Supplement: Supplementary file 1 [file DataSheet1.zip › Data/Figure/fig3/A.pdf]

# one-way ANOVA for chao index

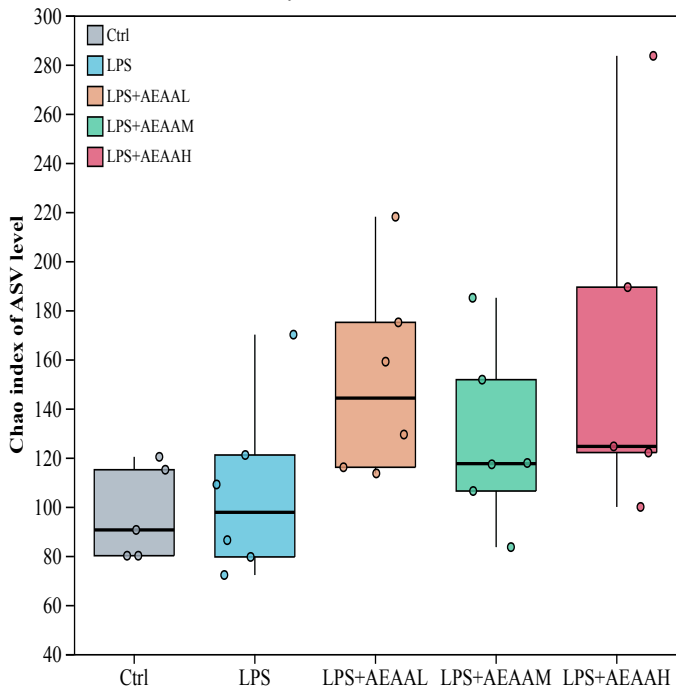

Supplement: Supplementary file 1 [file DataSheet1.zip › Data/Figure/fig3/B.pdf]

# PCoA on ASV level

$R = -0.2956$ ,  $P = 0.001$

PC2(14.09%)

- Ctrl
- LPS
- LPS+AEAAL
- LPS+AEAAM
- LPS+AEAAH

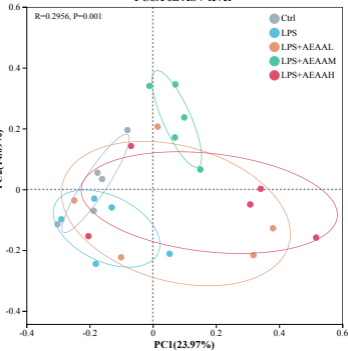

Supplement: Supplementary file 1 [file DataSheet1.zip › Data/Figure/fig3/C.pdf]

Genus

LPS

LPS+AEAAL

LPS+AEAAM

LPS+AEAAH

Ctrl

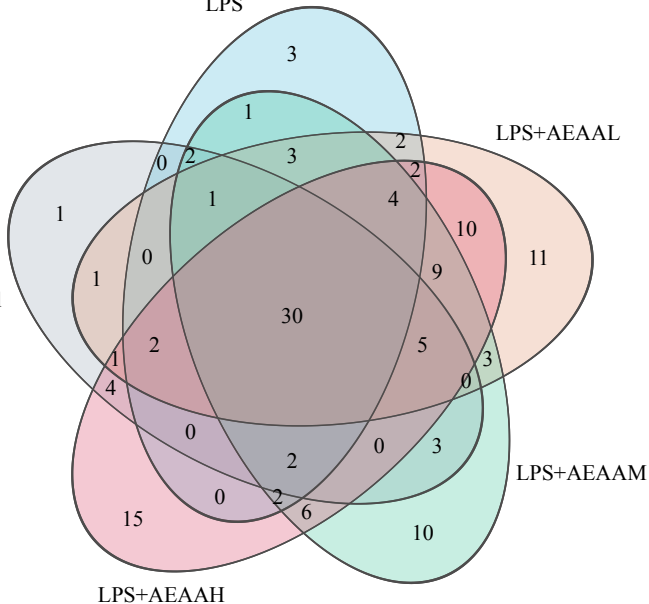

Supplement: Supplementary file 1 [file DataSheet1.zip › Data/Figure/fig3/D.pdf]

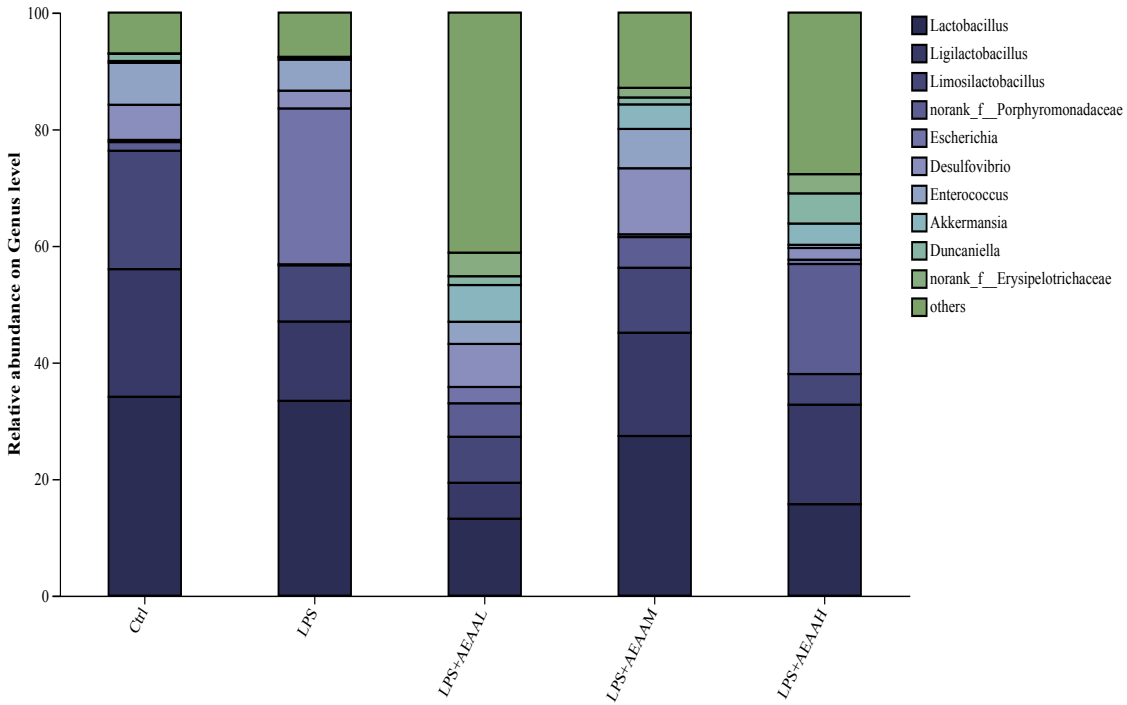

Supplement: Supplementary file 1 [file DataSheet1.zip › Data/Figure/fig3/E.pdf]

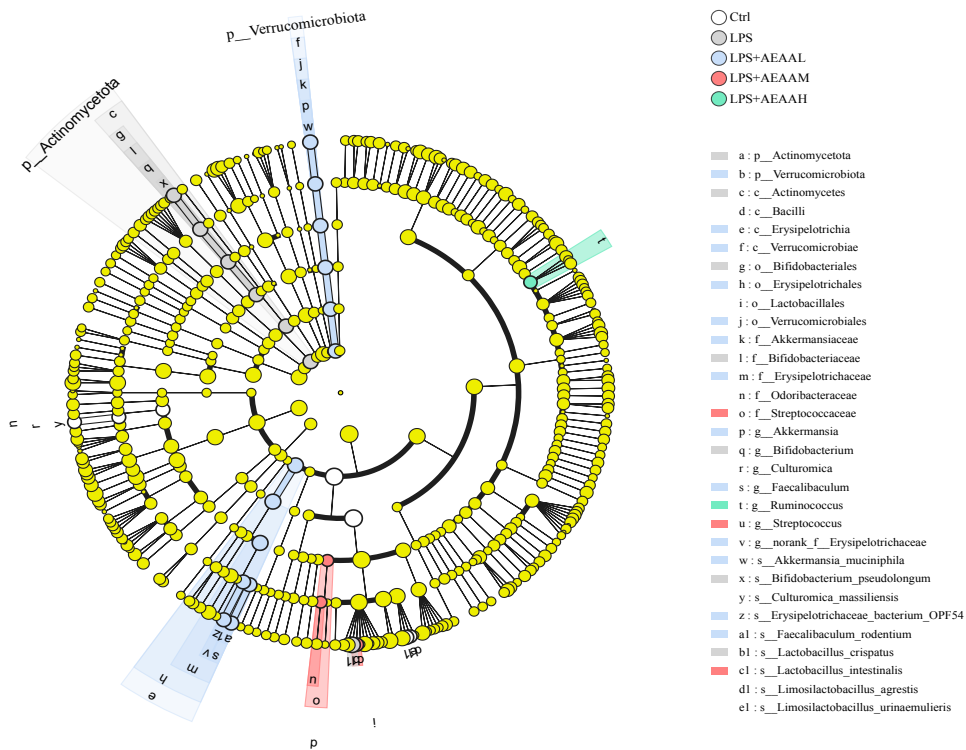

Supplement: Supplementary file 1 [file DataSheet1.zip › Data/Figure/fig3/F.pdf]

## Genus level

■ Ctrl  
■ LPS

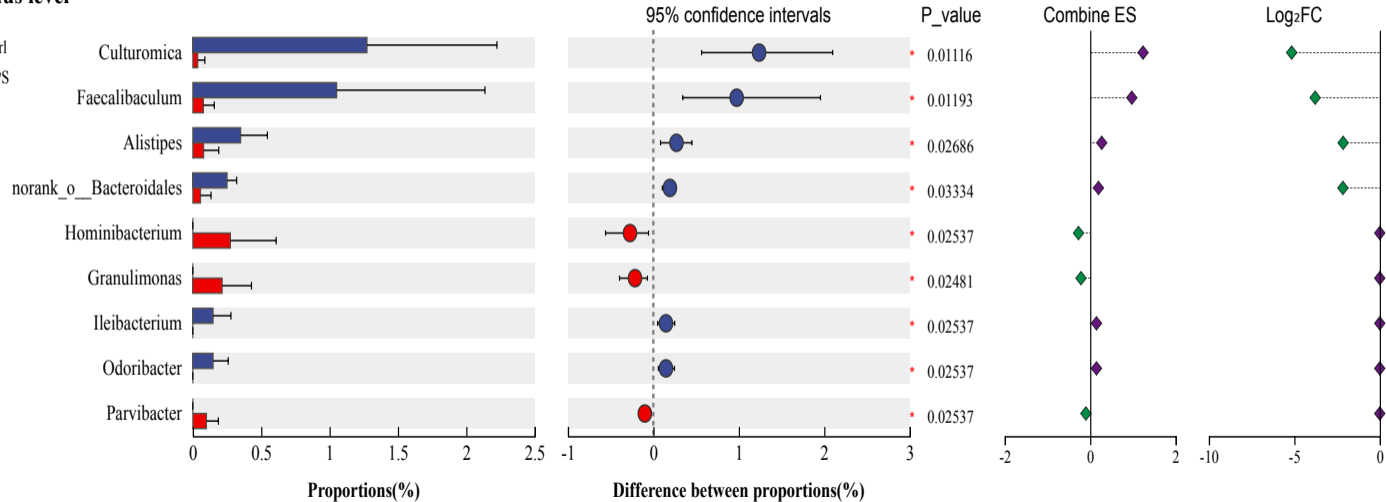

Supplement: Supplementary file 1 [file DataSheet1.zip › Data/Figure/fig3/G.pdf]

## Genus level

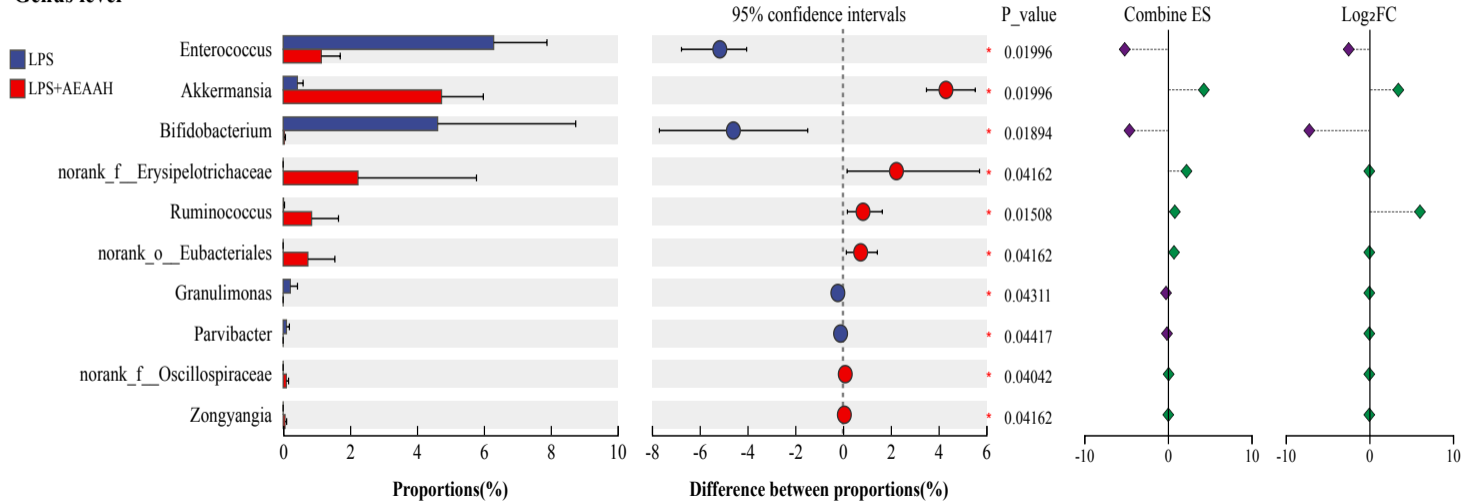

Supplement: Supplementary file 1 [file DataSheet1.zip › Data/Figure/fig3/H.pdf]

# Scores(PCA)

R=0.4636, P=0.001

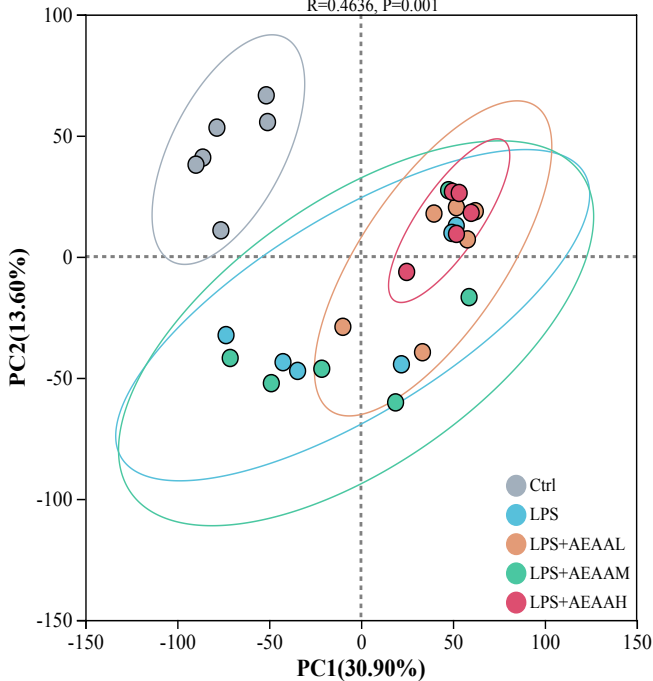

Supplement: Supplementary file 1 [file DataSheet1.zip › Data/Figure/fig4/A.pdf]

# Score(PLS-DA) plot

R=0.4636, P=0.001

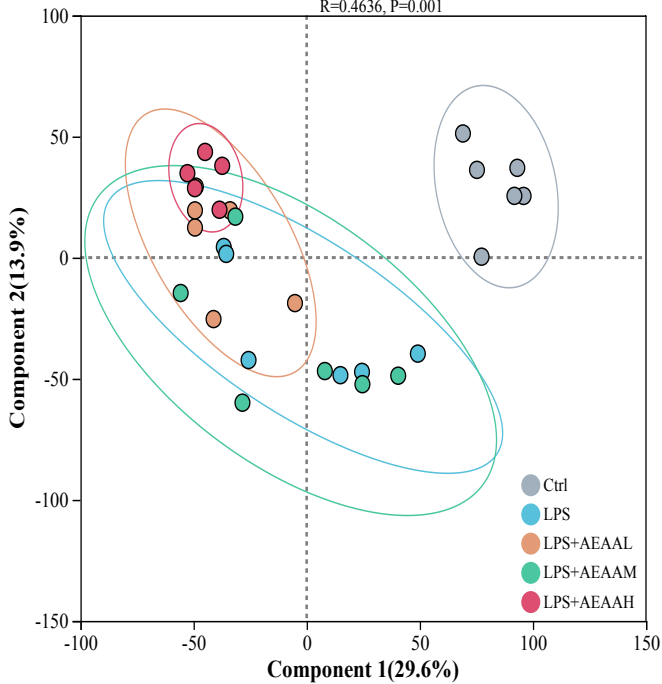

Supplement: Supplementary file 1 [file DataSheet1.zip › Data/Figure/fig4/B.pdf]

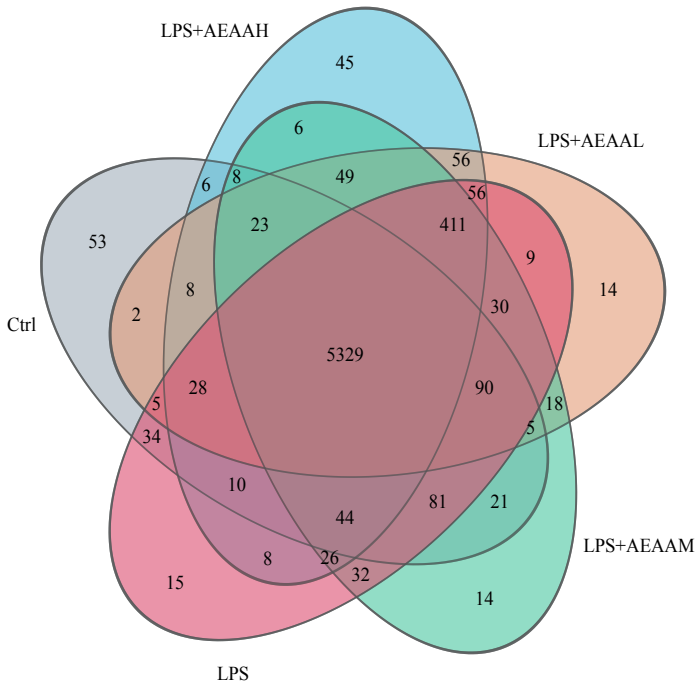

Supplement: Supplementary file 1 [file DataSheet1.zip › Data/Figure/fig4/C.pdf]

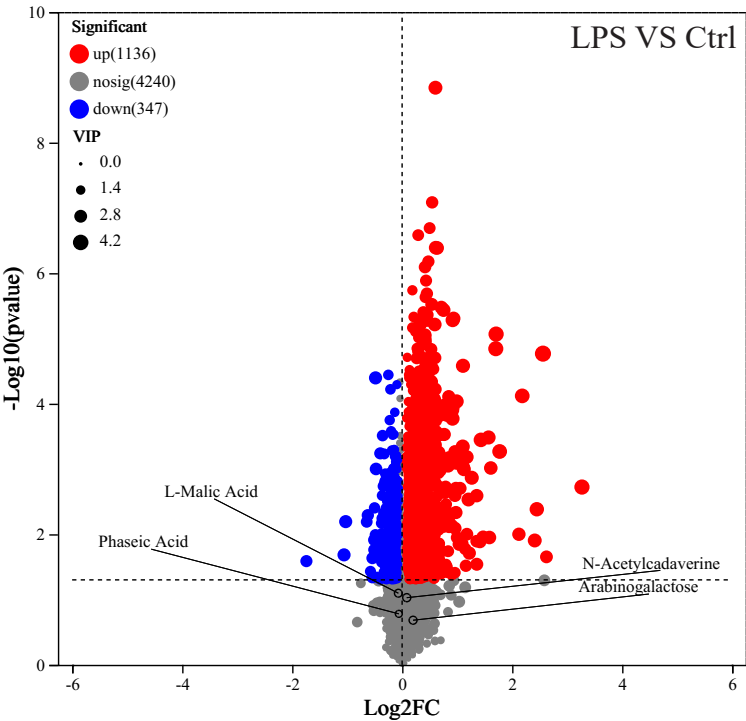

Supplement: Supplementary file 1 [file DataSheet1.zip › Data/Figure/fig4/D.pdf]

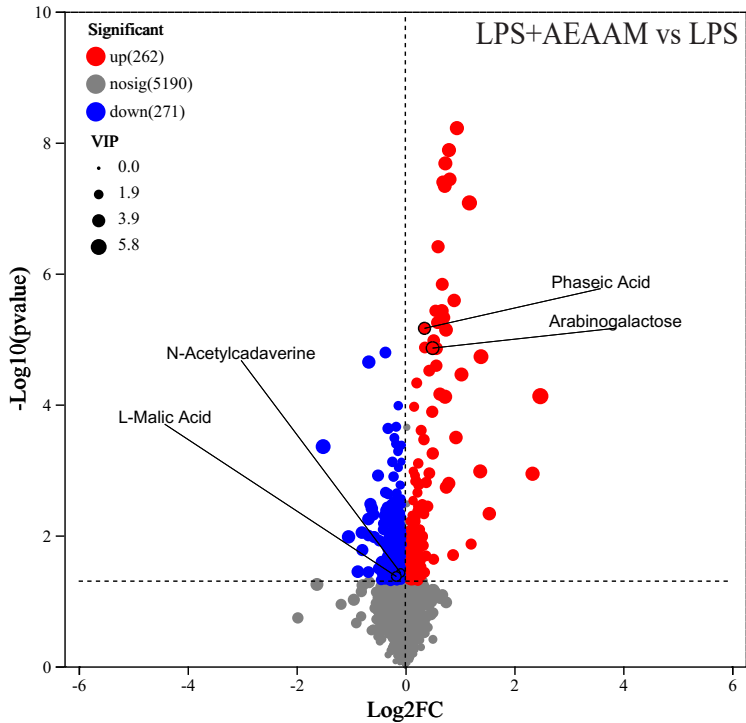

Supplement: Supplementary file 1 [file DataSheet1.zip › Data/Figure/fig4/E.pdf]

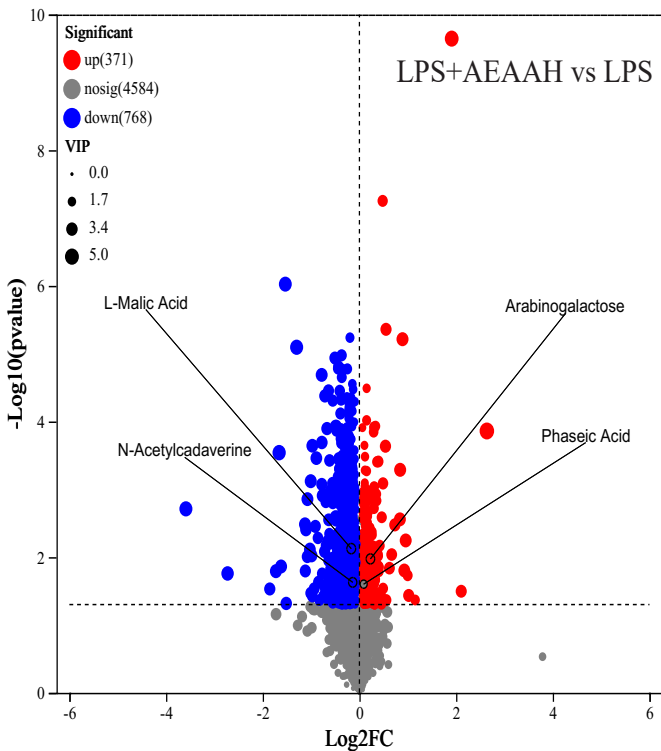

Supplement: Supplementary file 1 [file DataSheet1.zip › Data/Figure/fig4/F.pdf]

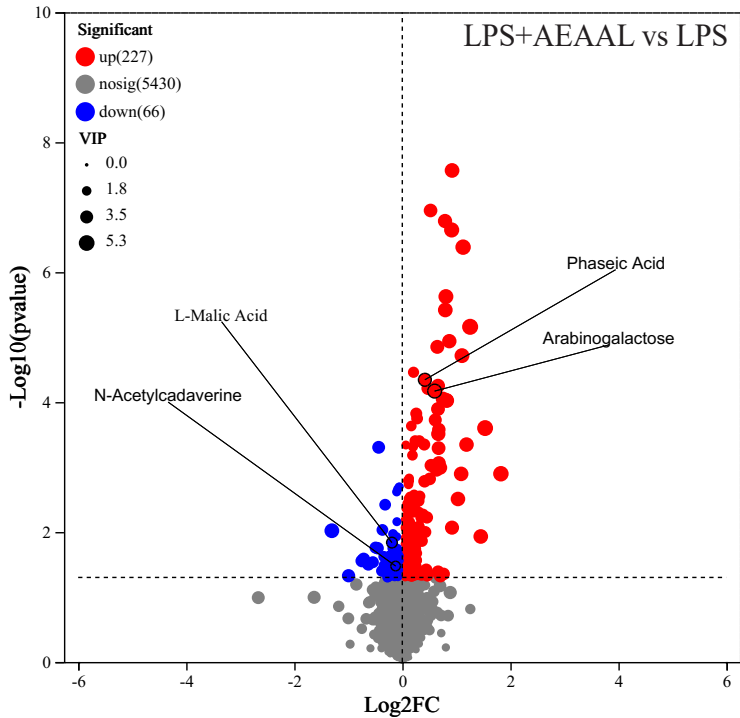

Supplement: Supplementary file 1 [file DataSheet1.zip › Data/Figure/fig4/G.pdf]

# Scores(PCA)

R=0.5652, P=0.001

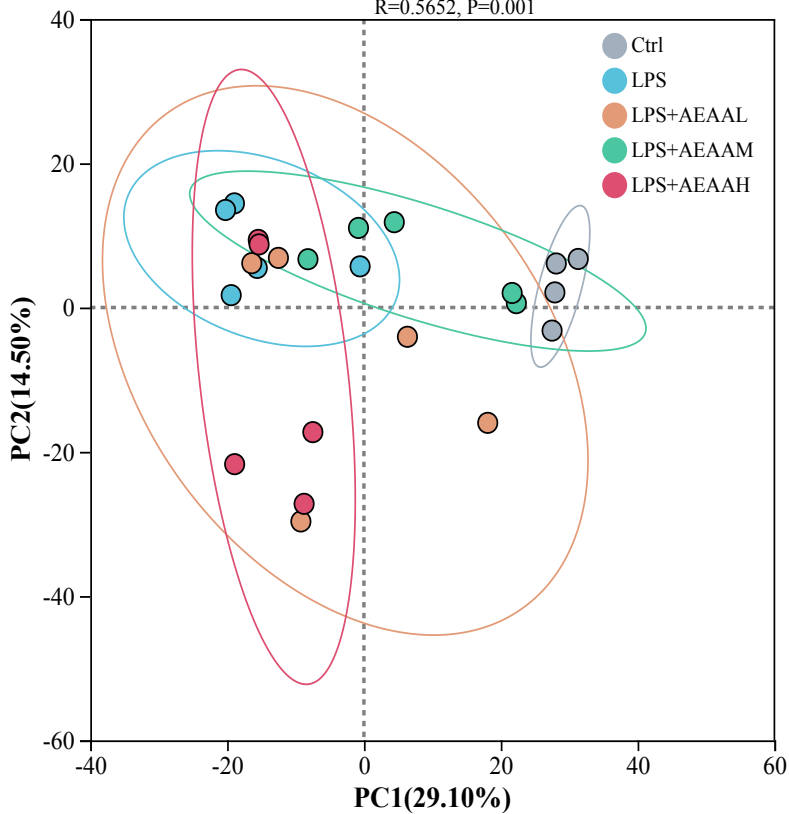

Supplement: Supplementary file 1 [file DataSheet1.zip › Data/Figure/fig5/A.pdf]

# Score(PLS-DA) plot

R=0.4825, P=0.001

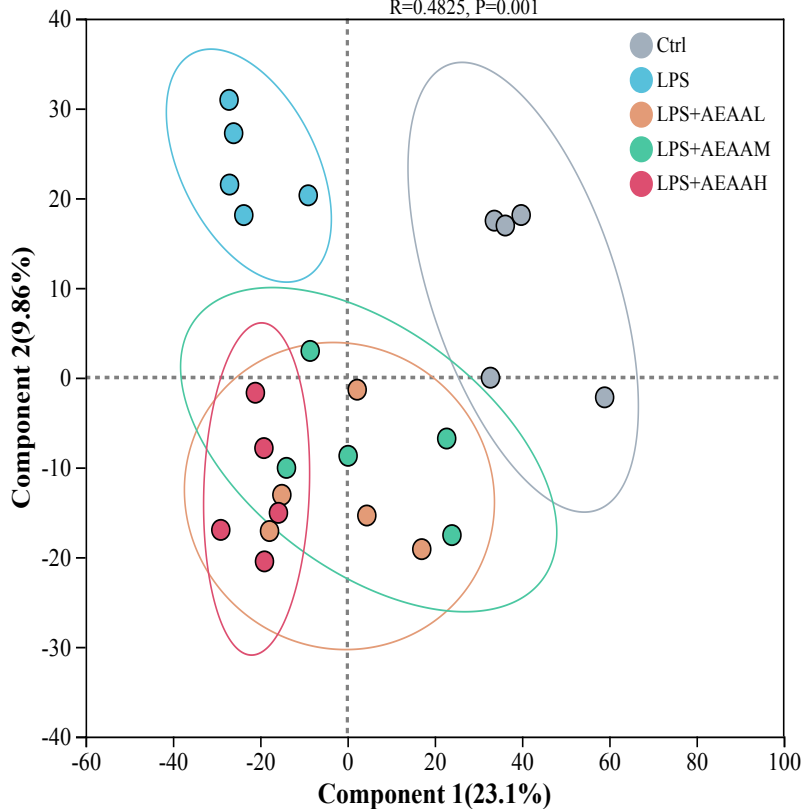

Supplement: Supplementary file 1 [file DataSheet1.zip › Data/Figure/fig5/B.pdf]

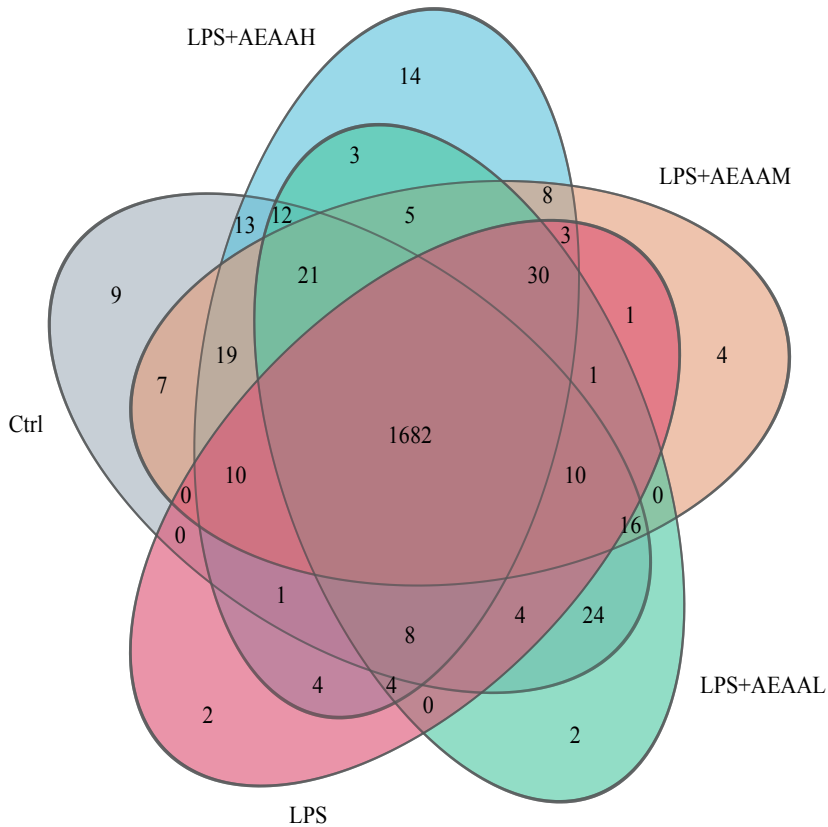

Supplement: Supplementary file 1 [file DataSheet1.zip › Data/Figure/fig5/C.pdf]

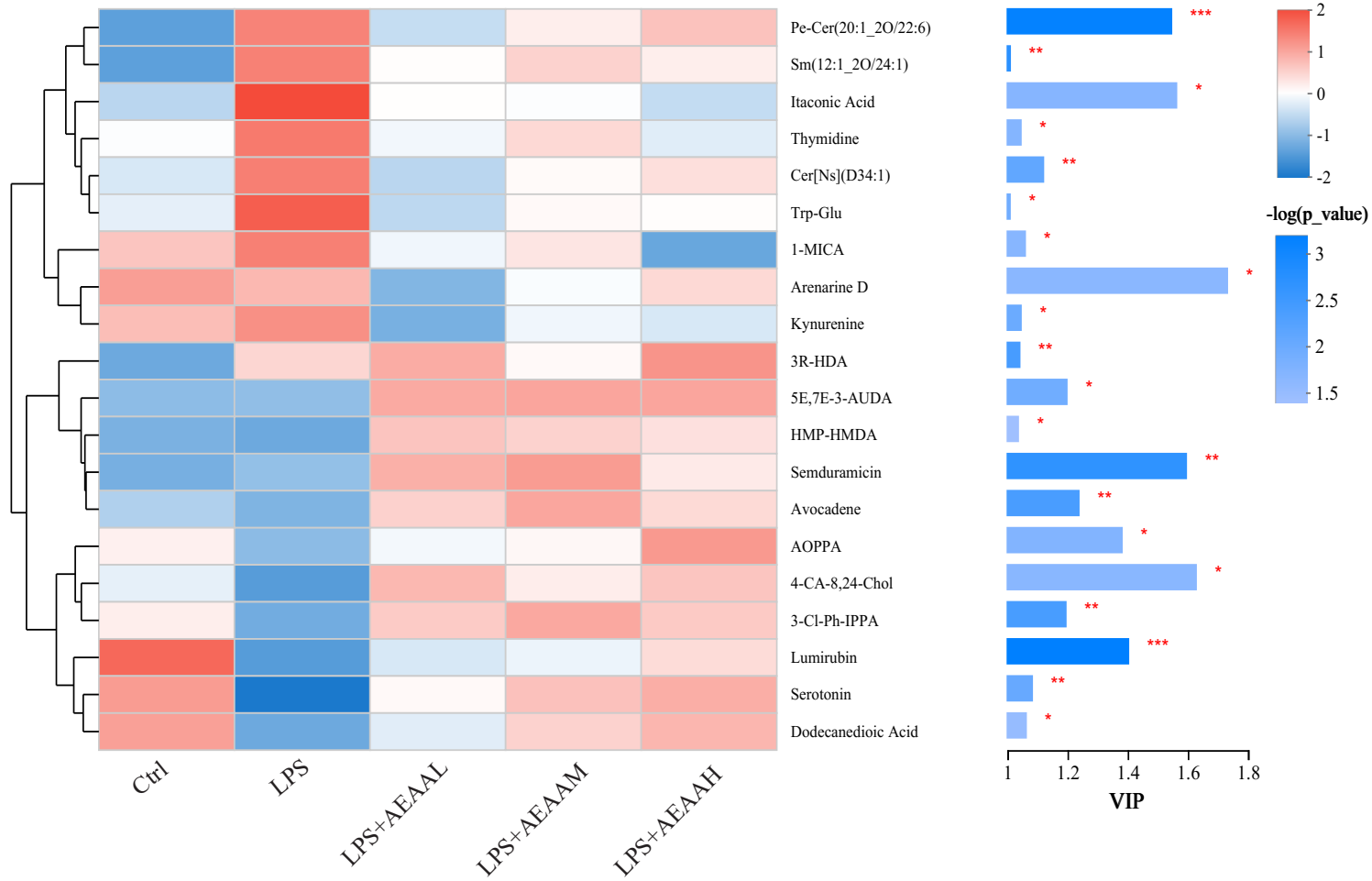

Supplement: Supplementary file 1 [file DataSheet1.zip › Data/Figure/fig5/D.pdf]

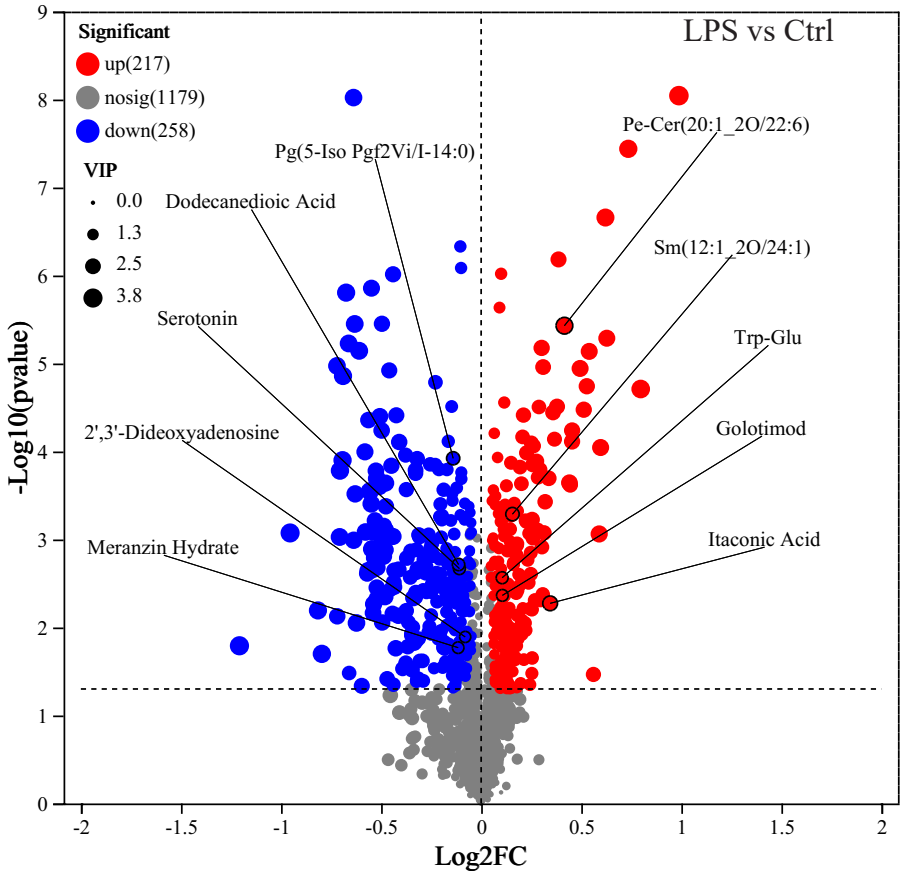

Supplement: Supplementary file 1 [file DataSheet1.zip › Data/Figure/fig5/E.pdf]

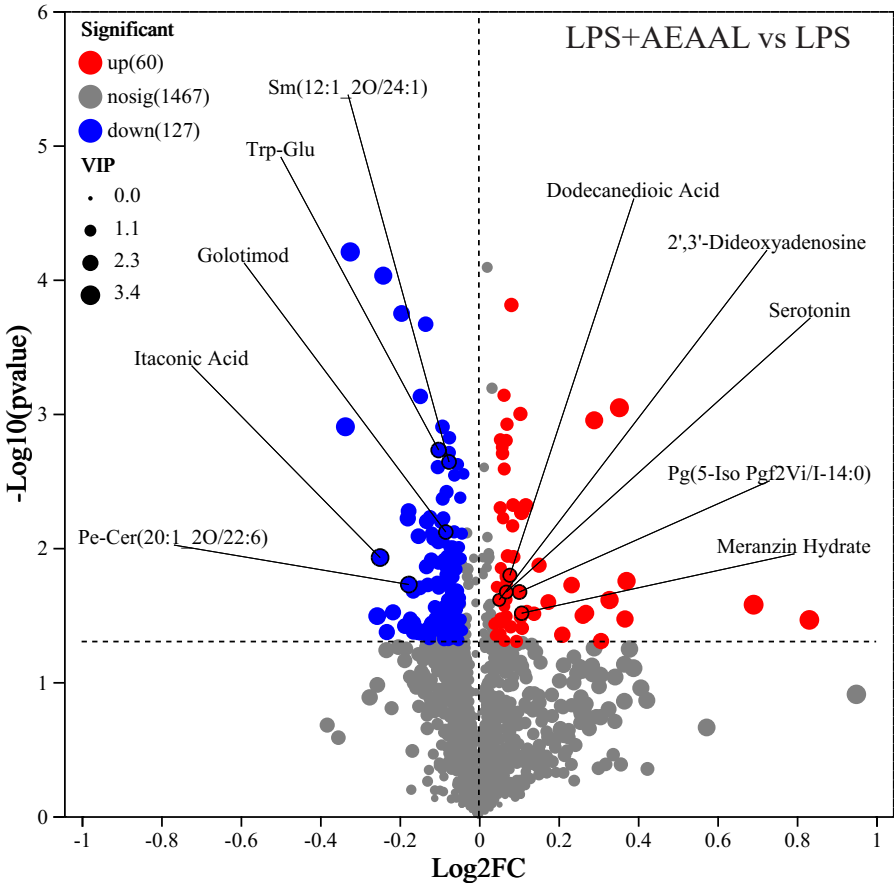

Supplement: Supplementary file 1 [file DataSheet1.zip › Data/Figure/fig5/F.pdf]

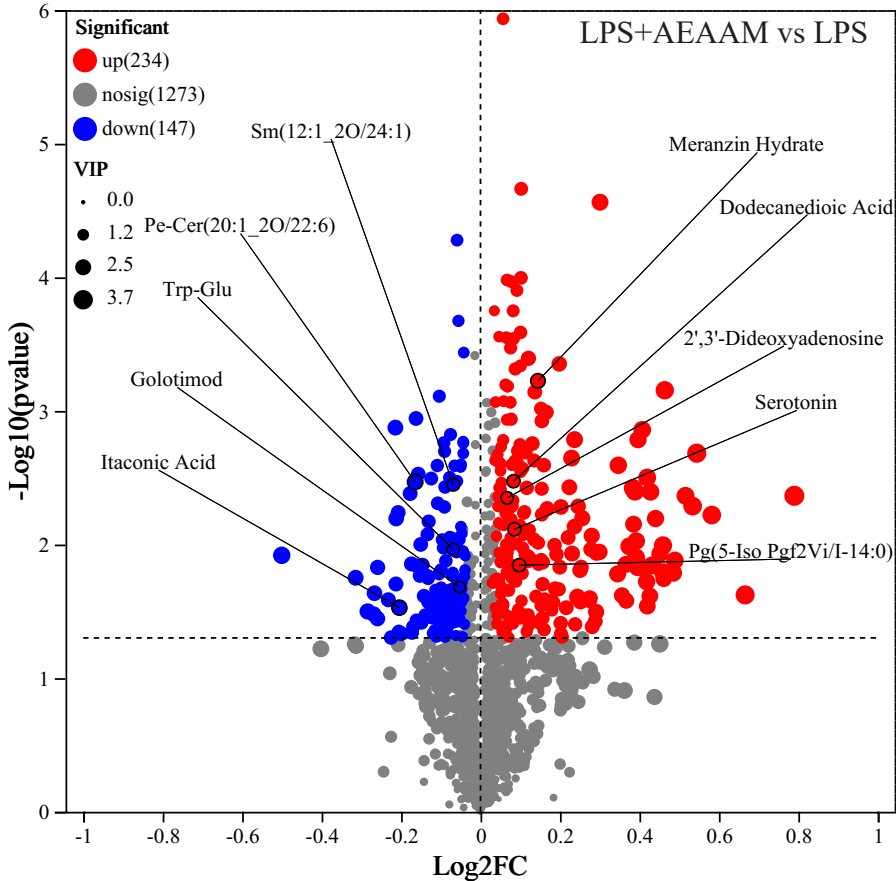

Supplement: Supplementary file 1 [file DataSheet1.zip › Data/Figure/fig5/G.pdf]

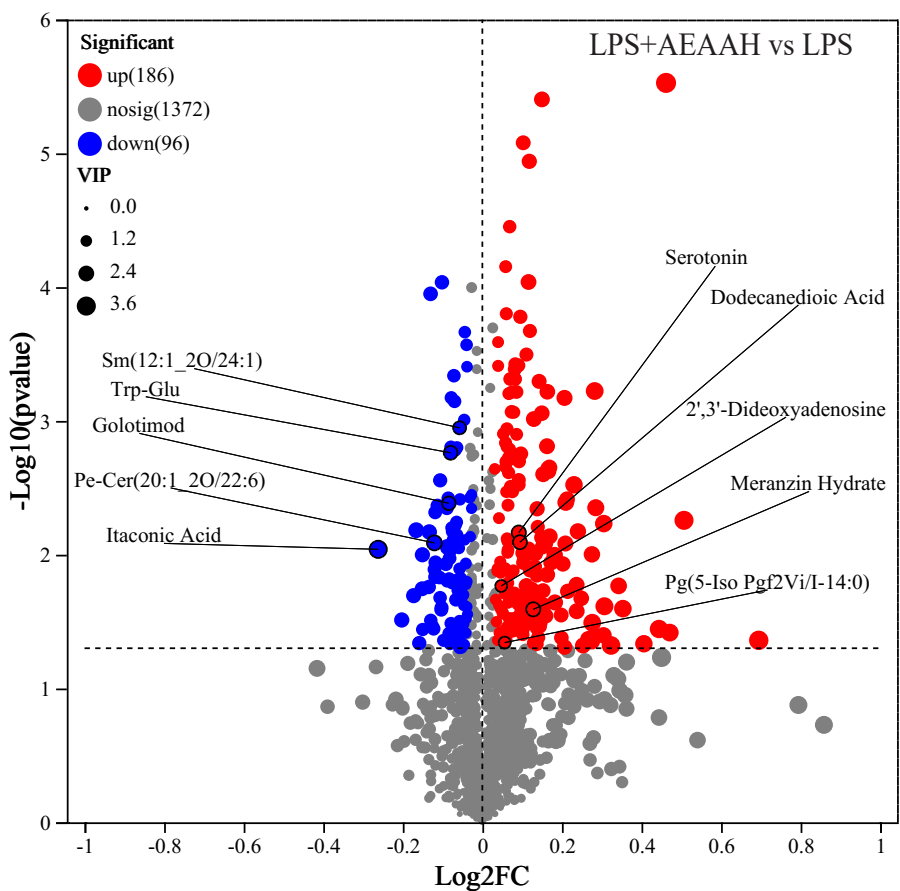

Supplement: Supplementary file 1 [file DataSheet1.zip › Data/Figure/fig5/H.pdf]

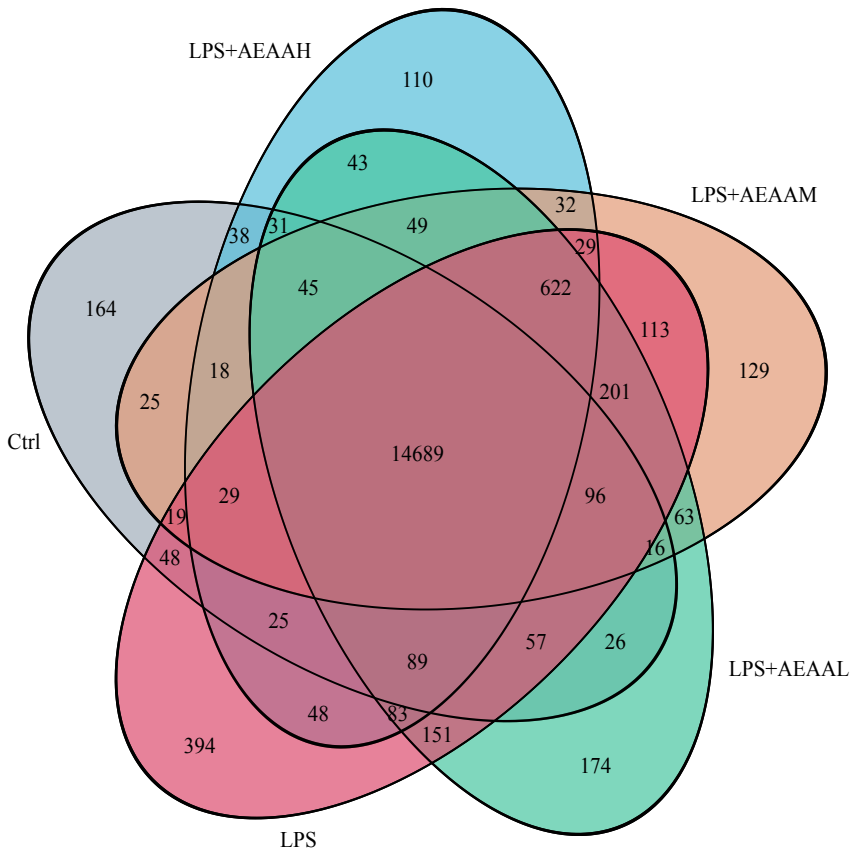

Supplement: Supplementary file 1 [file DataSheet1.zip › Data/Figure/fig6/A.pdf]

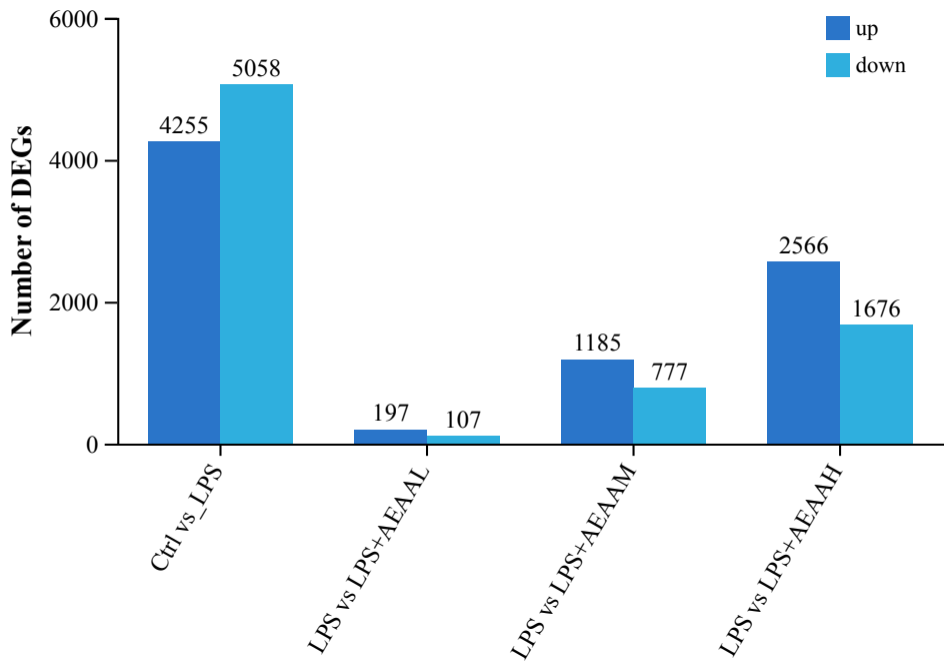

Supplement: Supplementary file 1 [file DataSheet1.zip › Data/Figure/fig6/B.pdf]

# PCA analysis

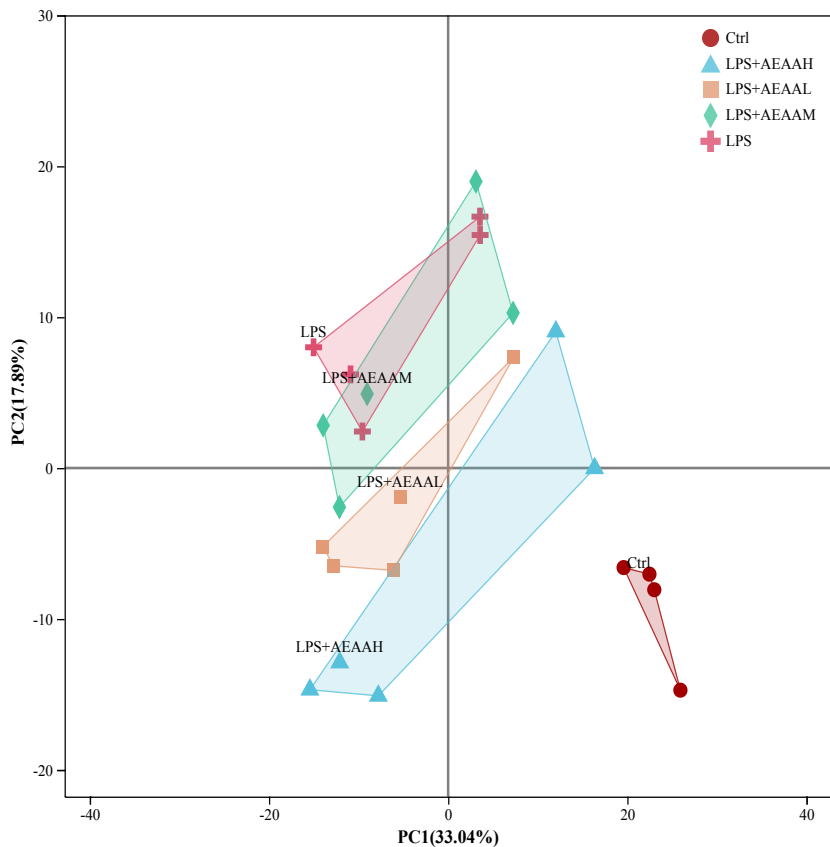

Supplement: Supplementary file 1 [file DataSheet1.zip › Data/Figure/fig6/C.pdf]

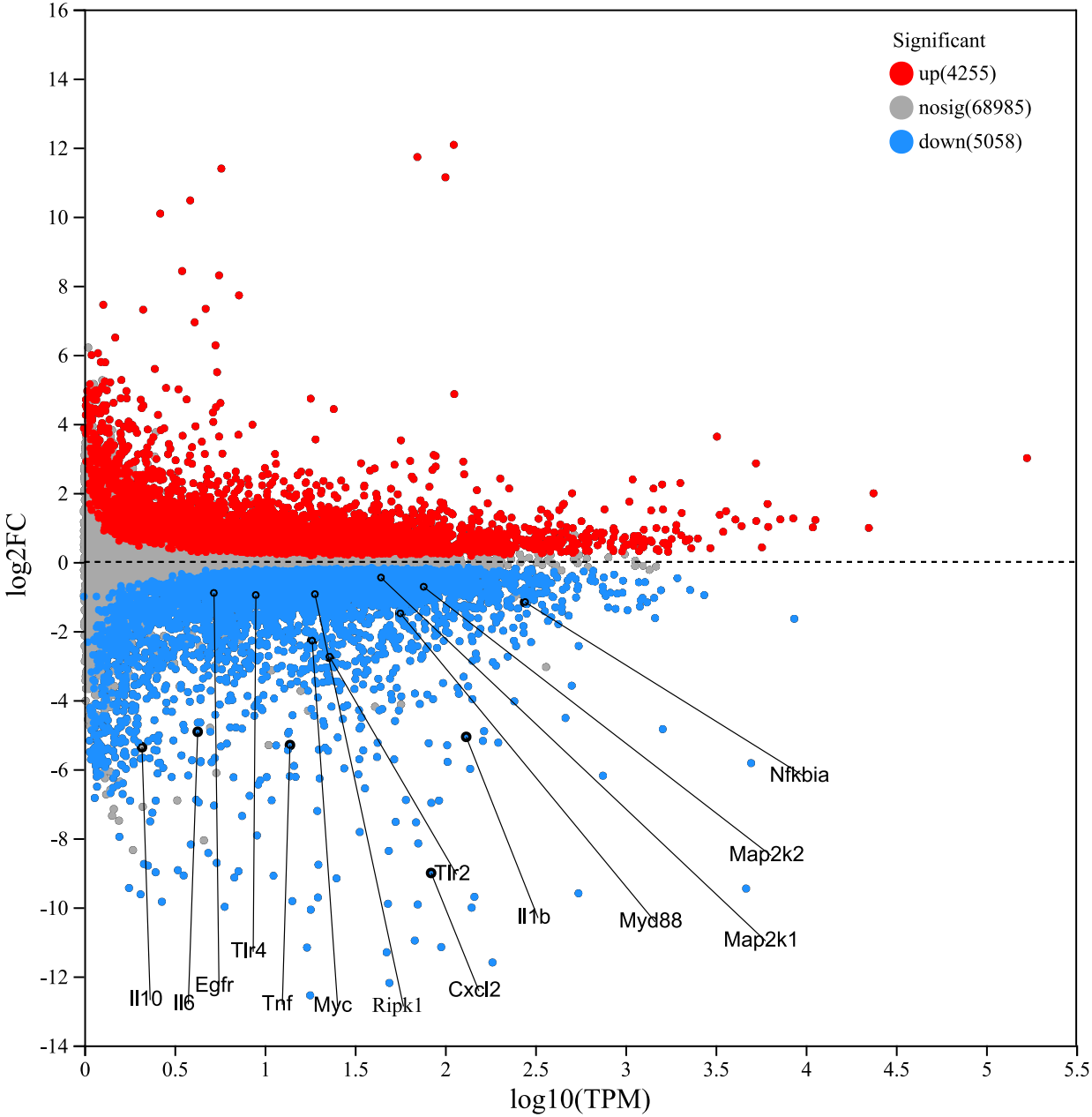

Supplement: Supplementary file 1 [file DataSheet1.zip › Data/Figure/fig6/D.pdf]

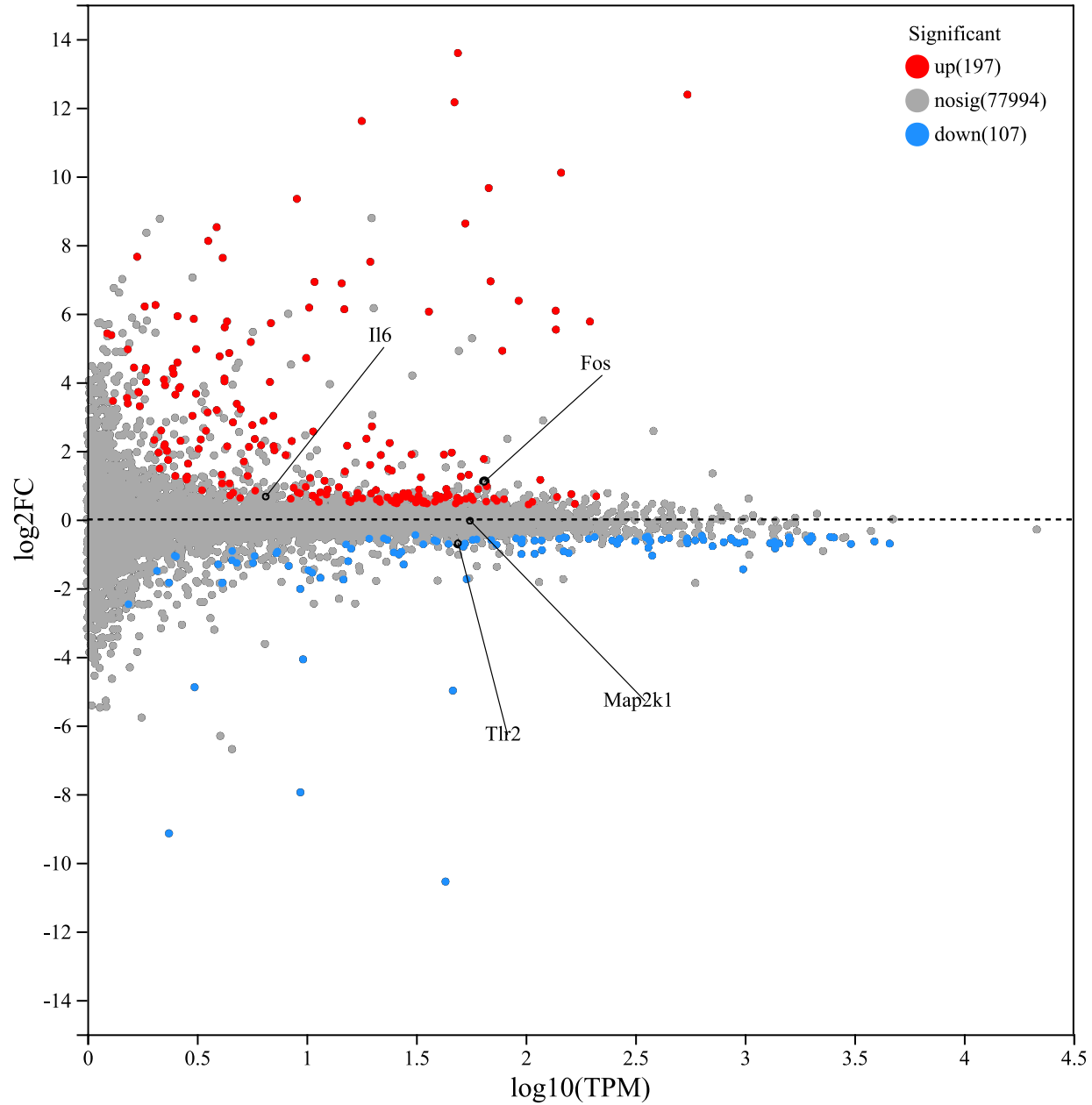

Supplement: Supplementary file 1 [file DataSheet1.zip › Data/Figure/fig6/E.pdf]

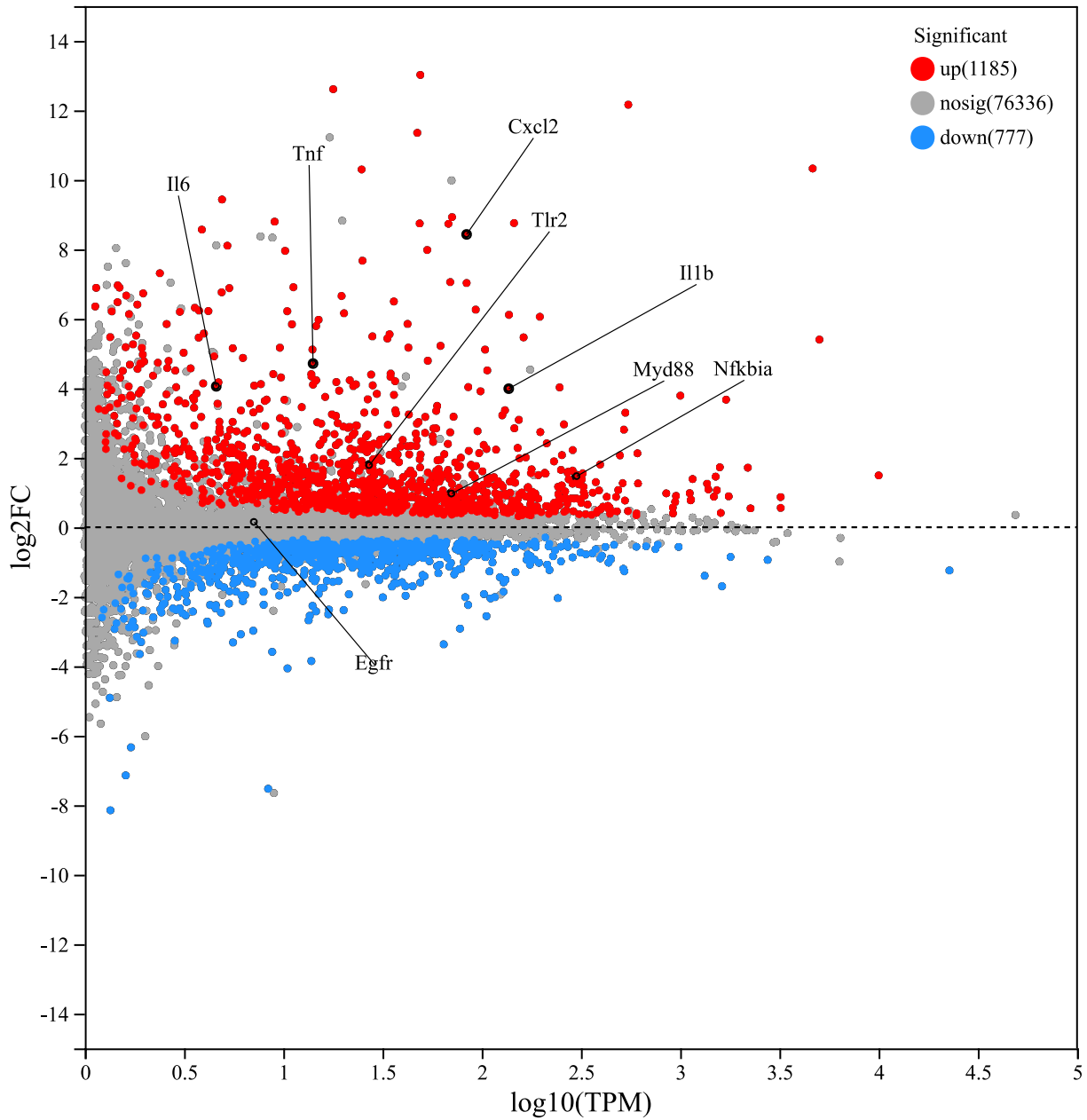

Supplement: Supplementary file 1 [file DataSheet1.zip › Data/Figure/fig6/F.pdf]

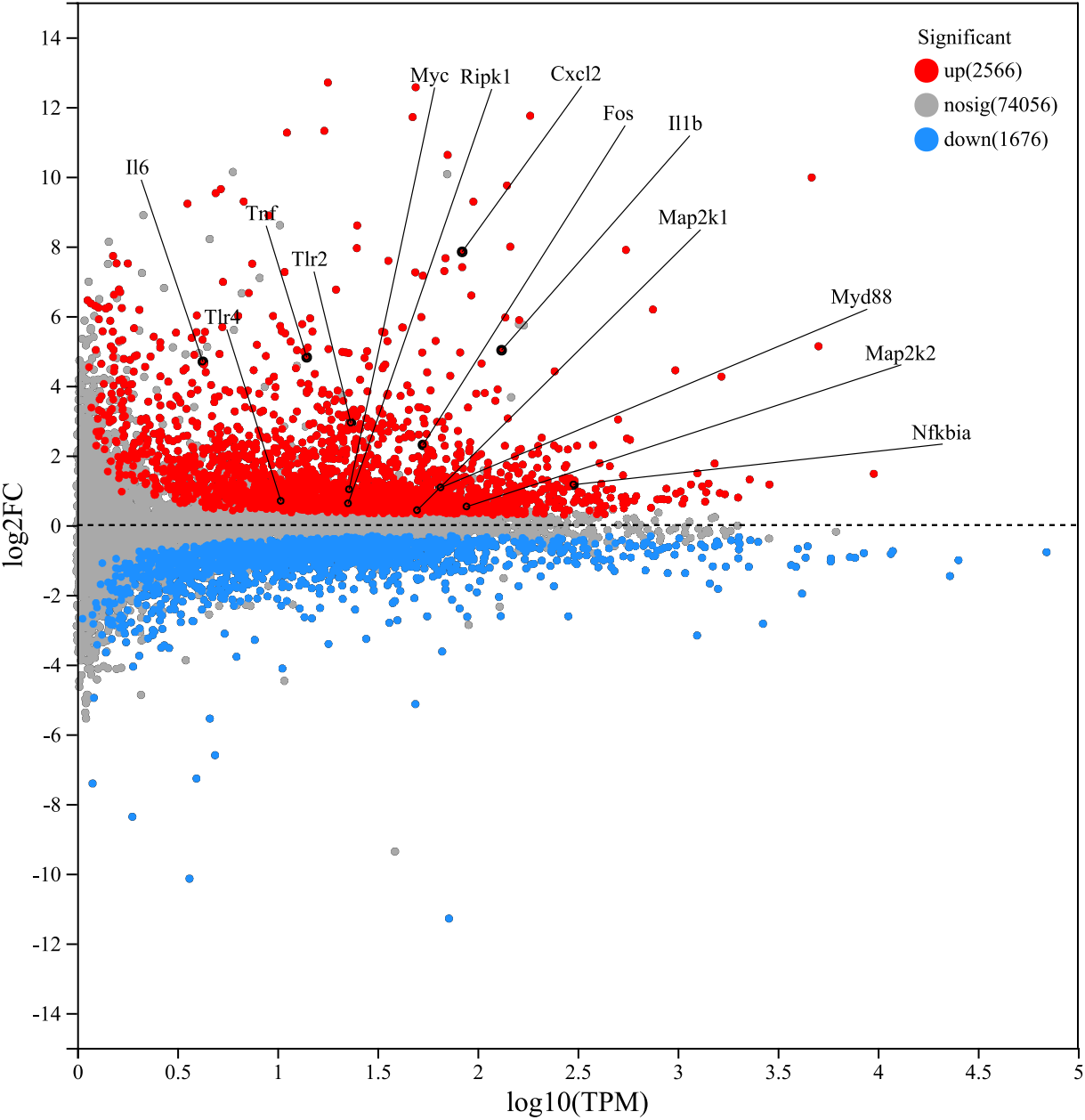

Supplement: Supplementary file 1 [file DataSheet1.zip › Data/Figure/fig6/G.pdf]

# Ctrl vs LPS

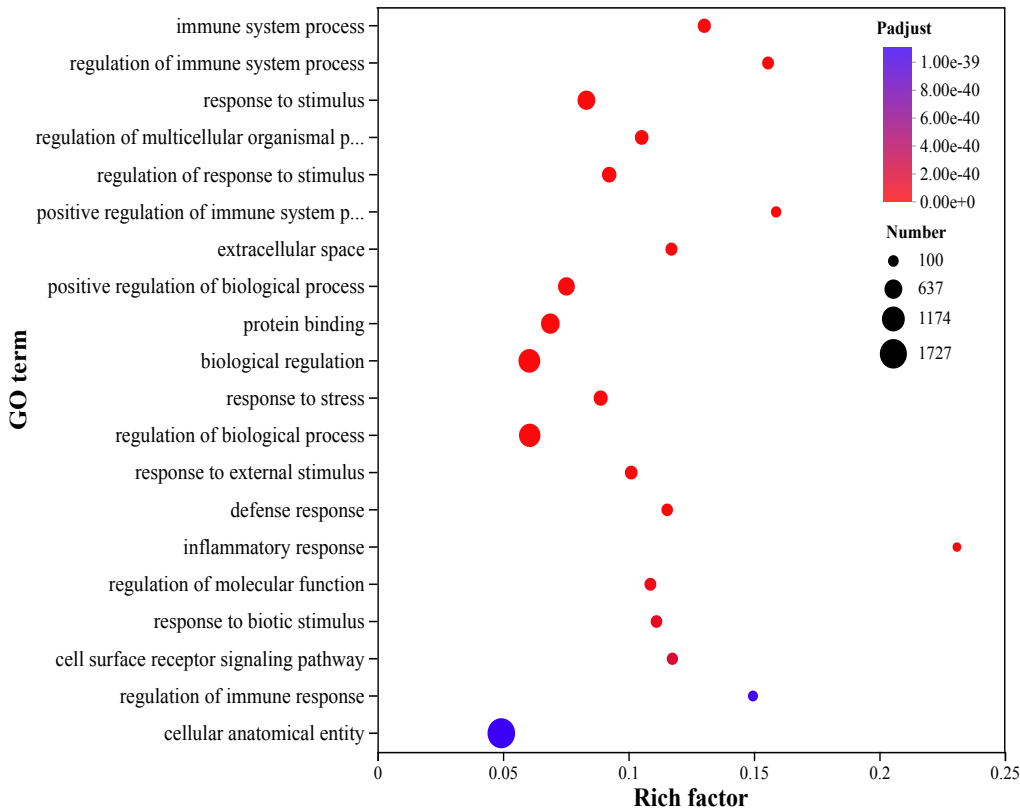

Supplement: Supplementary file 1 [file DataSheet1.zip › Data/Figure/fig6/H.pdf]

# LPS vs LPS+AEAAH

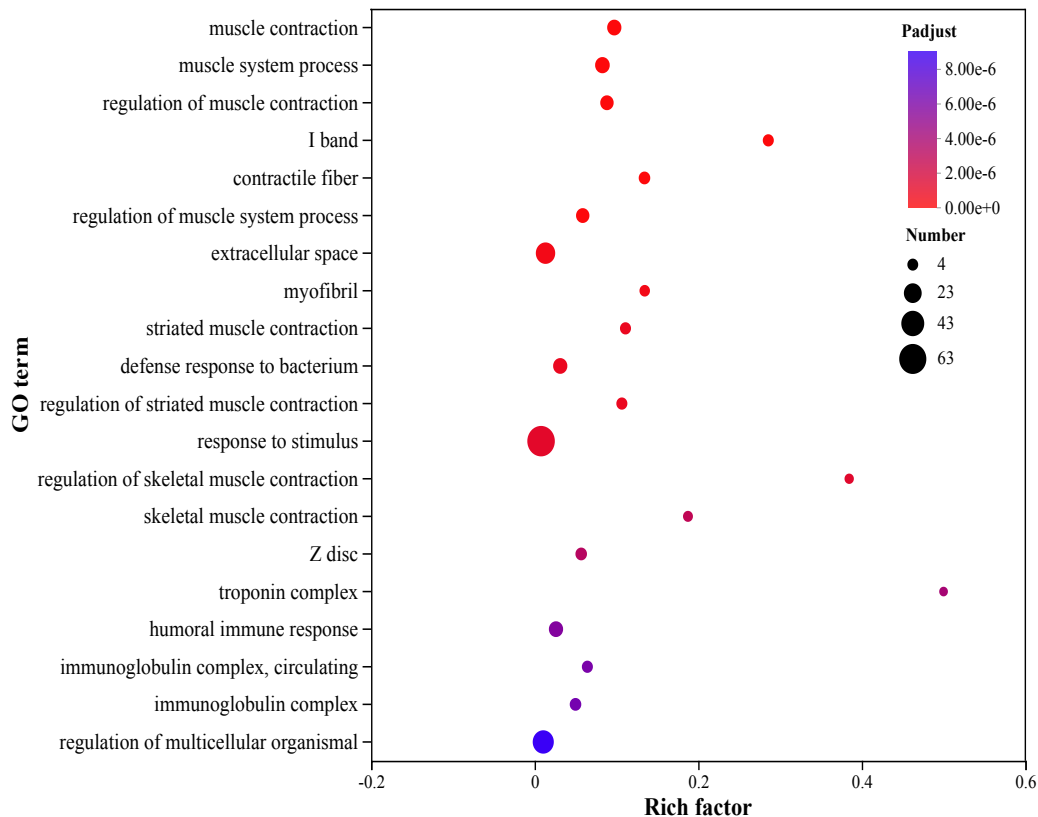

Supplement: Supplementary file 1 [file DataSheet1.zip › Data/Figure/fig6/I.pdf]

Ctrl vs LPS

Number of genes

KEGG pathway

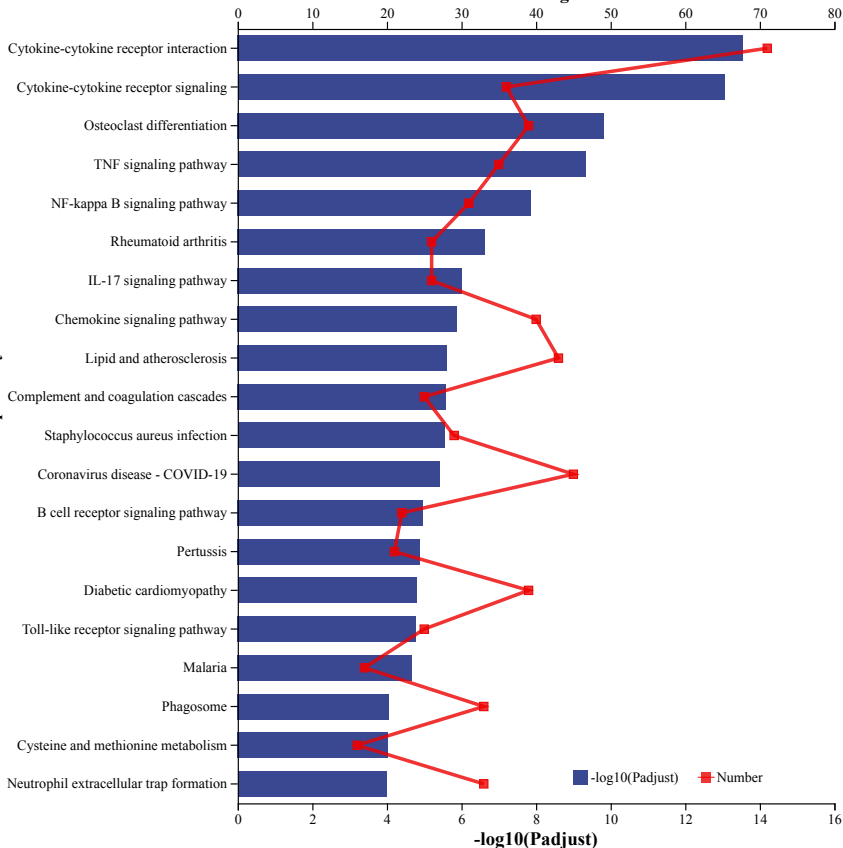

Supplement: Supplementary file 1 [file DataSheet1.zip › Data/Figure/fig6/J.pdf]

# LPS vs LPS+AEA AH

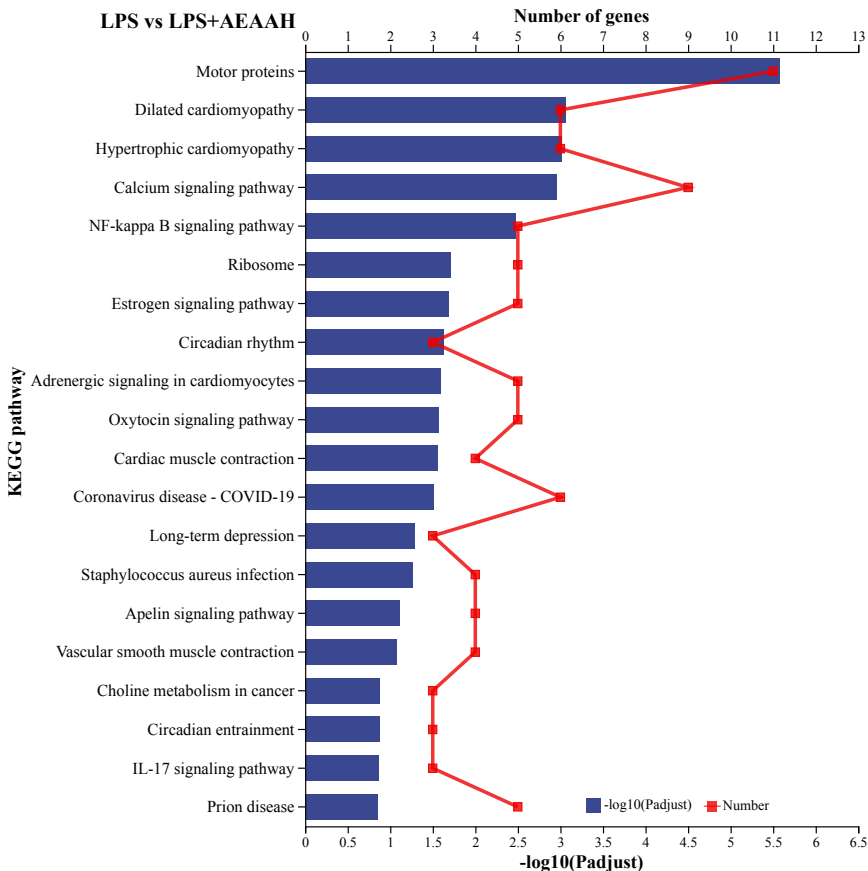

Supplement: Supplementary file 1 [file DataSheet1.zip › Data/Figure/fig6/K.pdf]

# Target gene

GO term

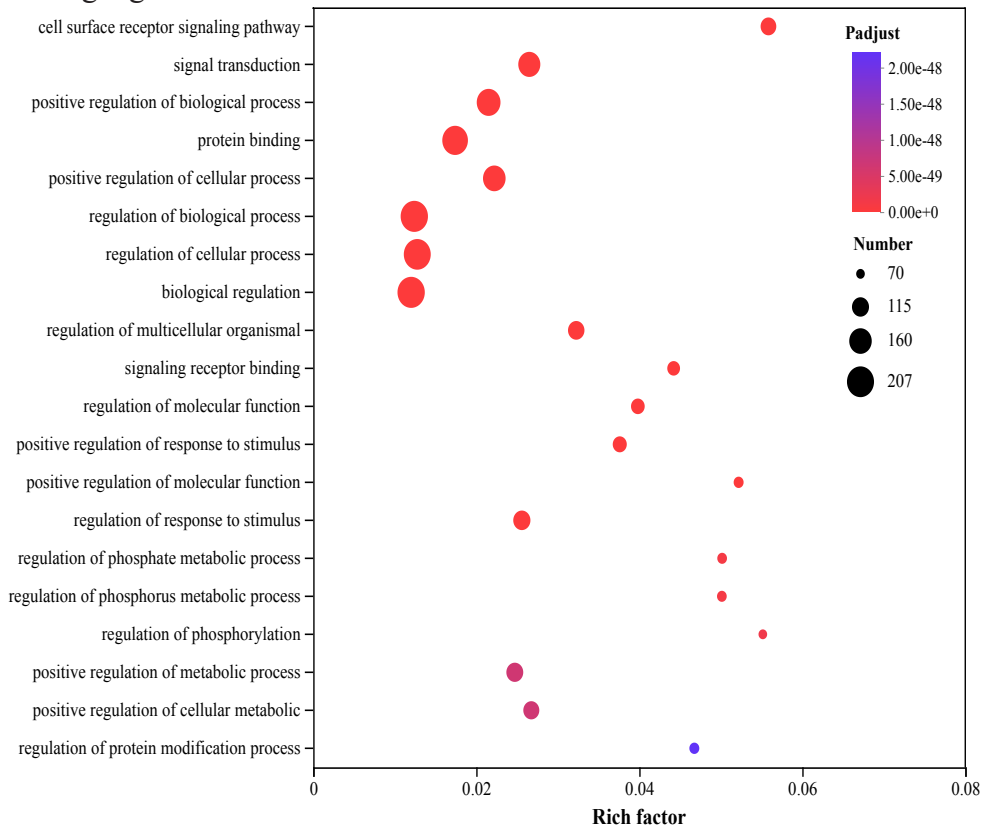

Supplement: Supplementary file 1 [file DataSheet1.zip › Data/Figure/fig6/L.pdf]

# Target gene

Number of genes

KEGG pathway

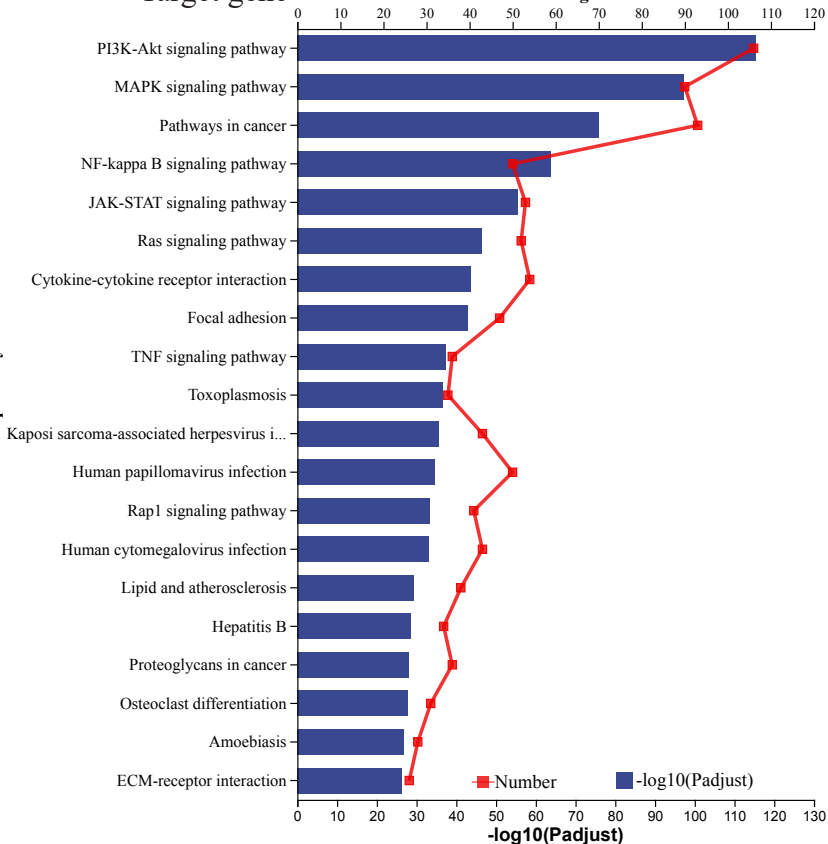

Supplement: Supplementary file 1 [file DataSheet1.zip › Data/Figure/fig6/M.pdf]

# Expression of NF $\kappa$ B pathway genes in mice lungs

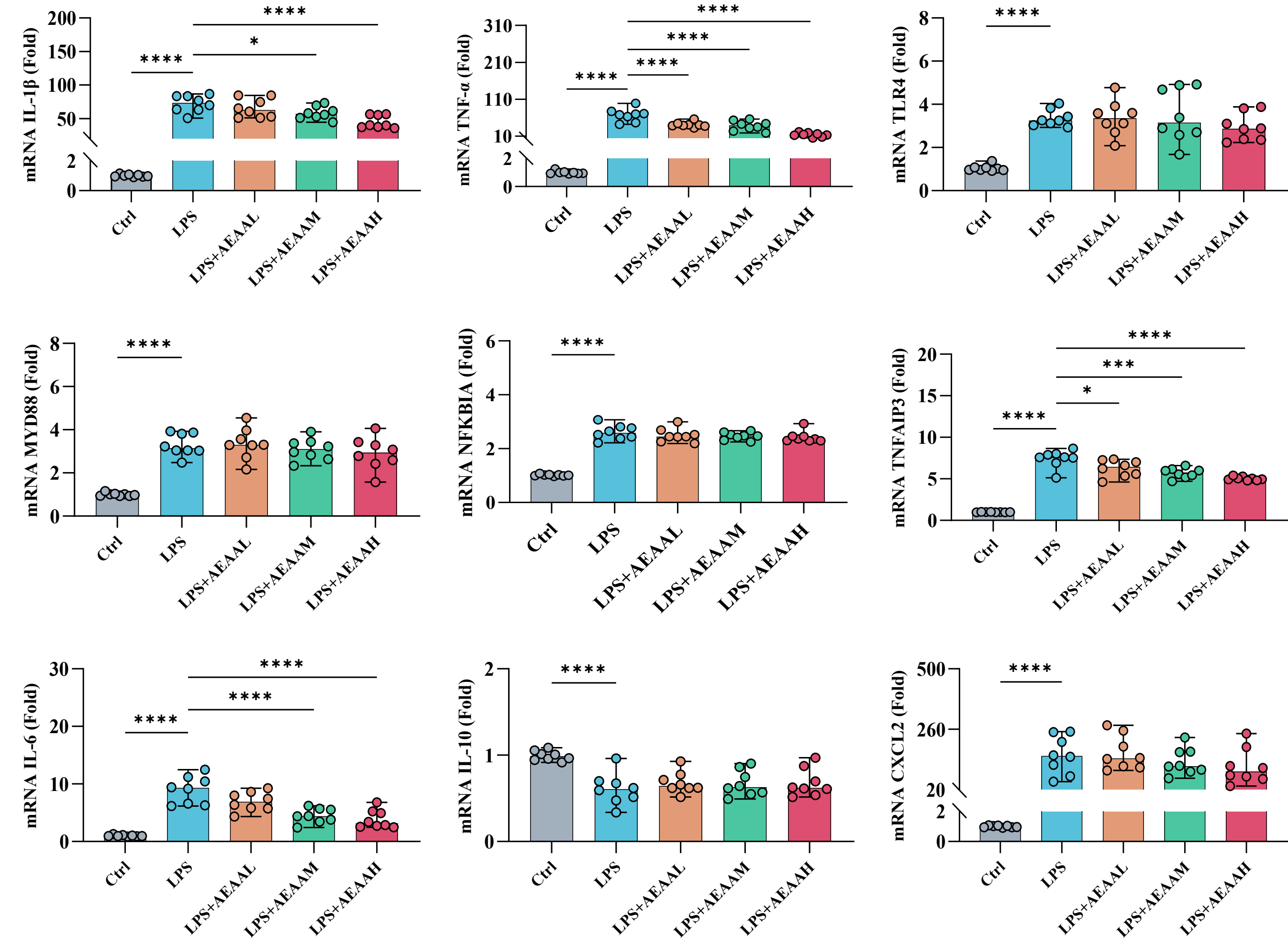

Supplement: Supplementary file 1 [file DataSheet1.zip › Data/Figure/fig6/N.pdf]

# Expression of MAPK pathway genes in mice lungs

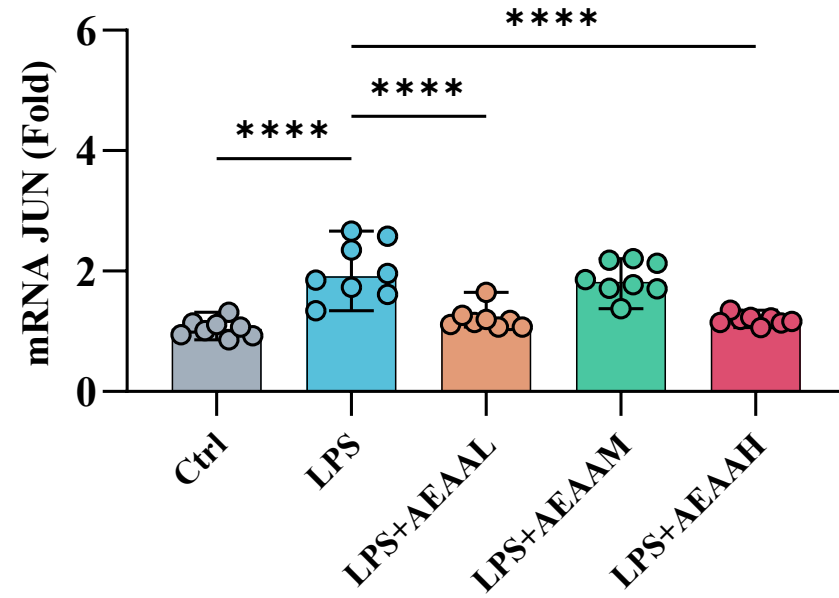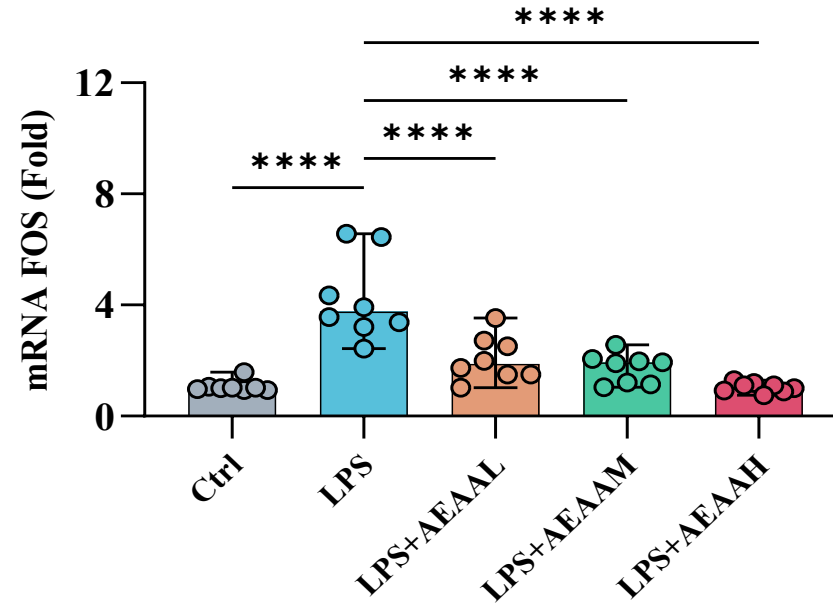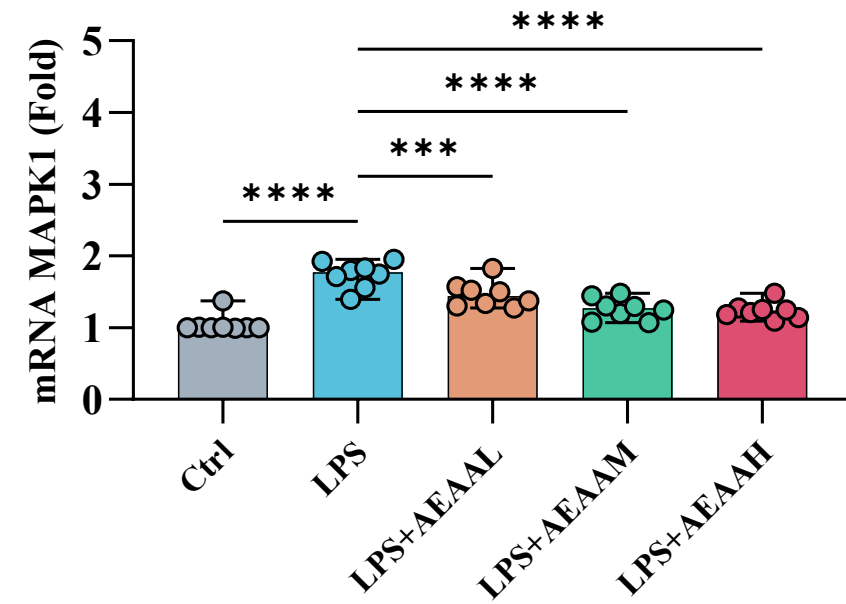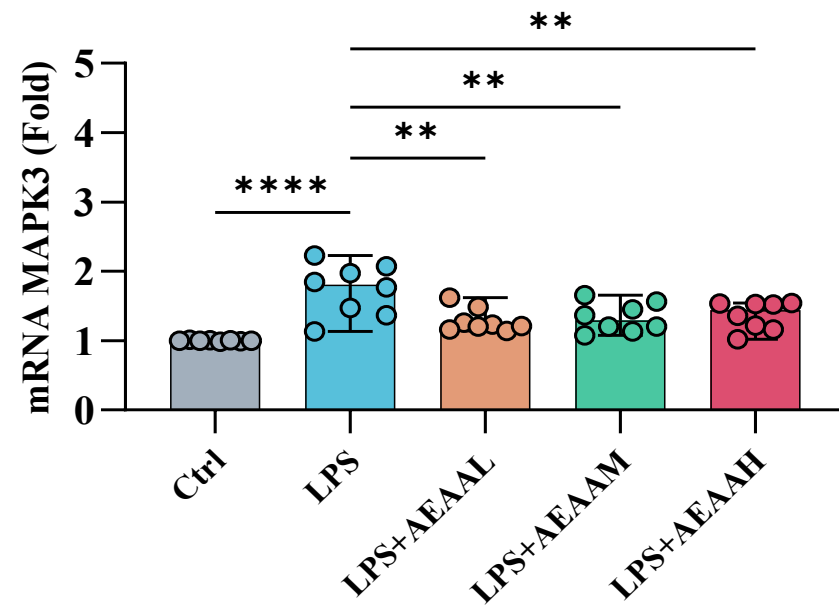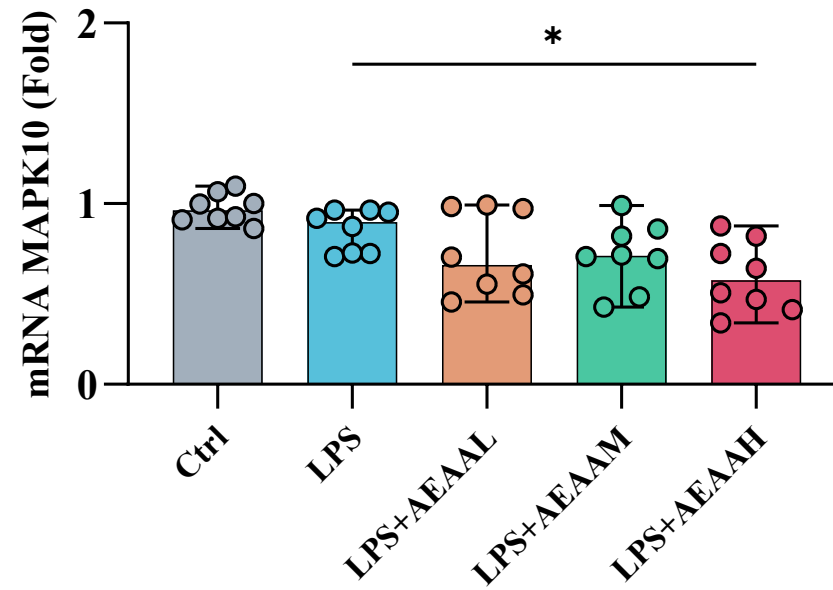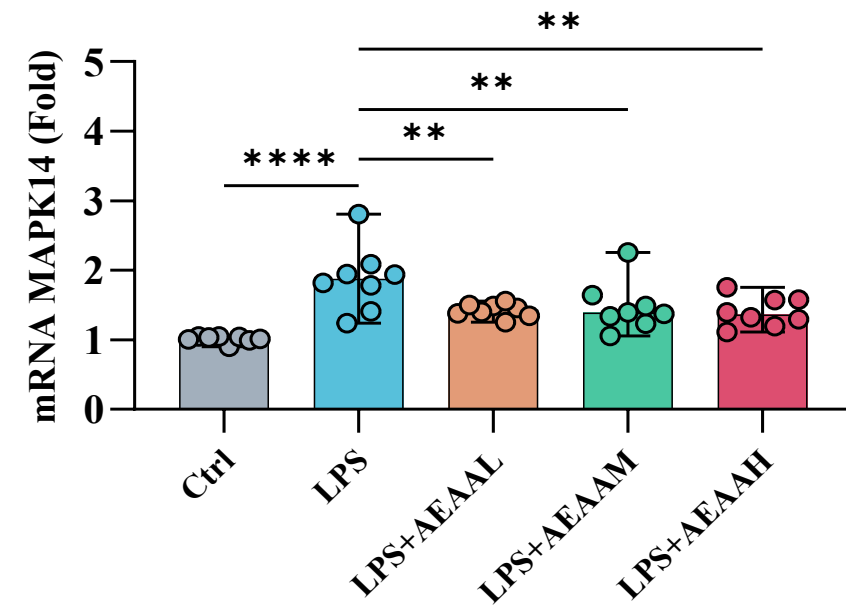

Supplement: Supplementary file 1 [file DataSheet1.zip › Data/Figure/fig6/O.pdf]

# Expression of PI3K-AKT pathway genes in mice lungs

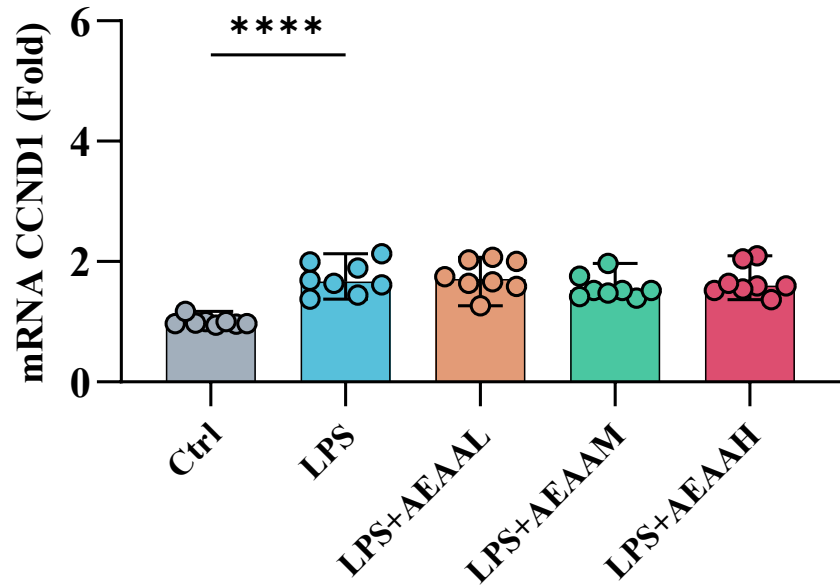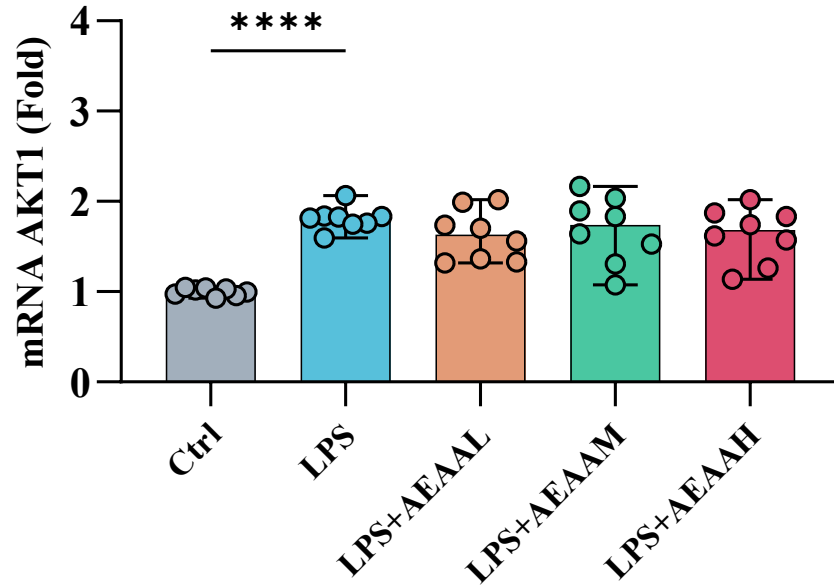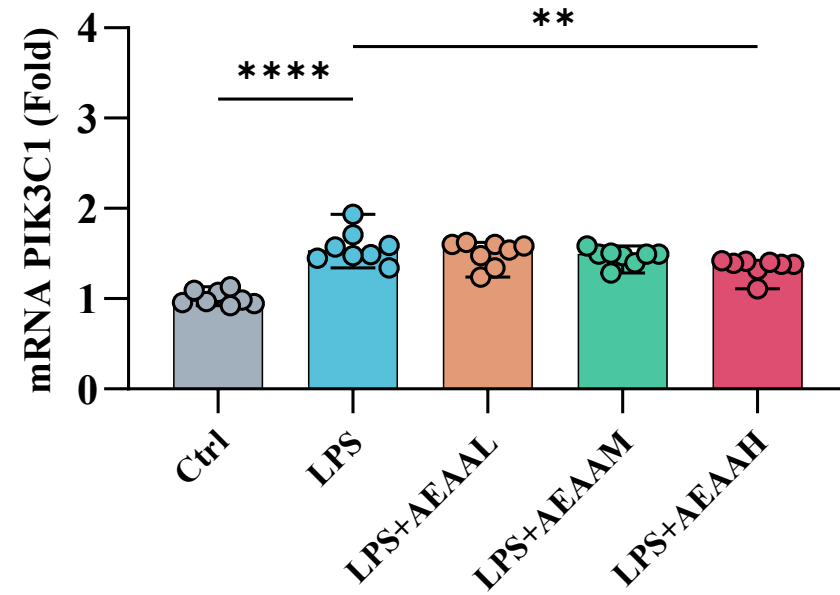

Supplement: Supplementary file 1 [file DataSheet1.zip › Data/Figure/fig6/P.pdf]

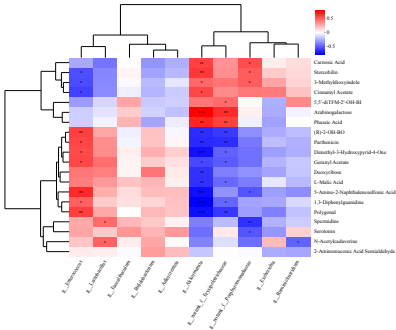

Supplement: Supplementary file 1 [file DataSheet1.zip › Data/Figure/fig7/A.pdf]

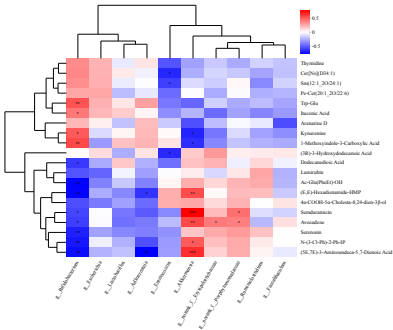

Supplement: Supplementary file 1 [file DataSheet1.zip › Data/Figure/fig7/B.pdf]

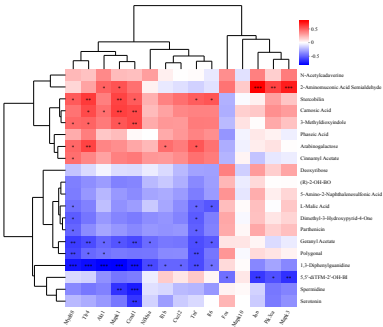

Supplement: Supplementary file 1 [file DataSheet1.zip › Data/Figure/fig7/C.pdf]

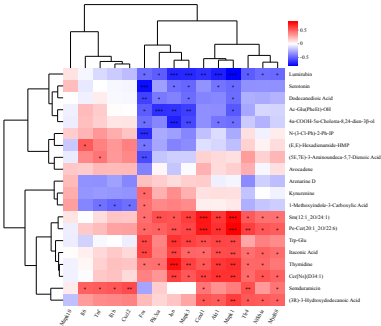

Supplement: Supplementary file 1 [file DataSheet1.zip › Data/Figure/fig7/D.pdf]

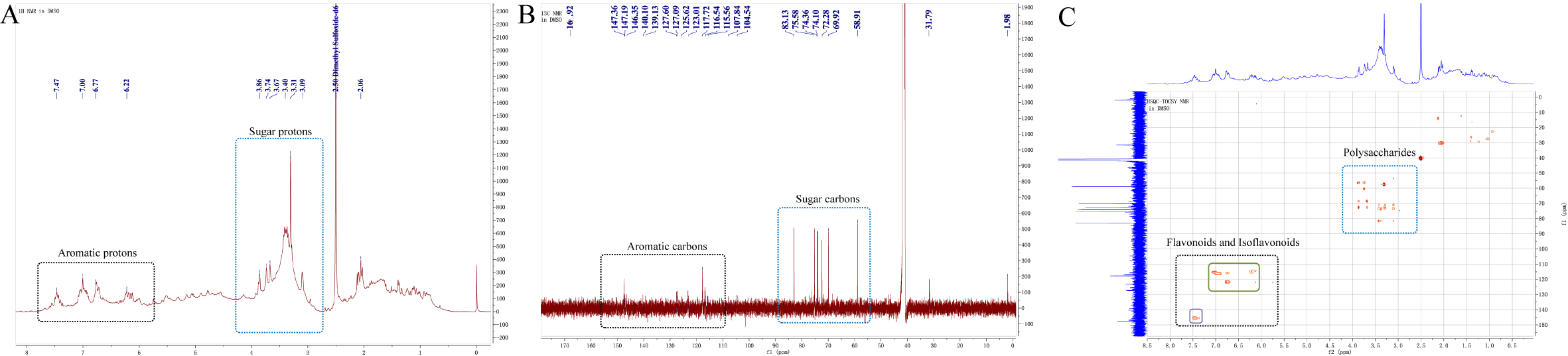

Supplement: Supplementary file 1 [file DataSheet1.zip › Data/Figure/Figure1.tif]

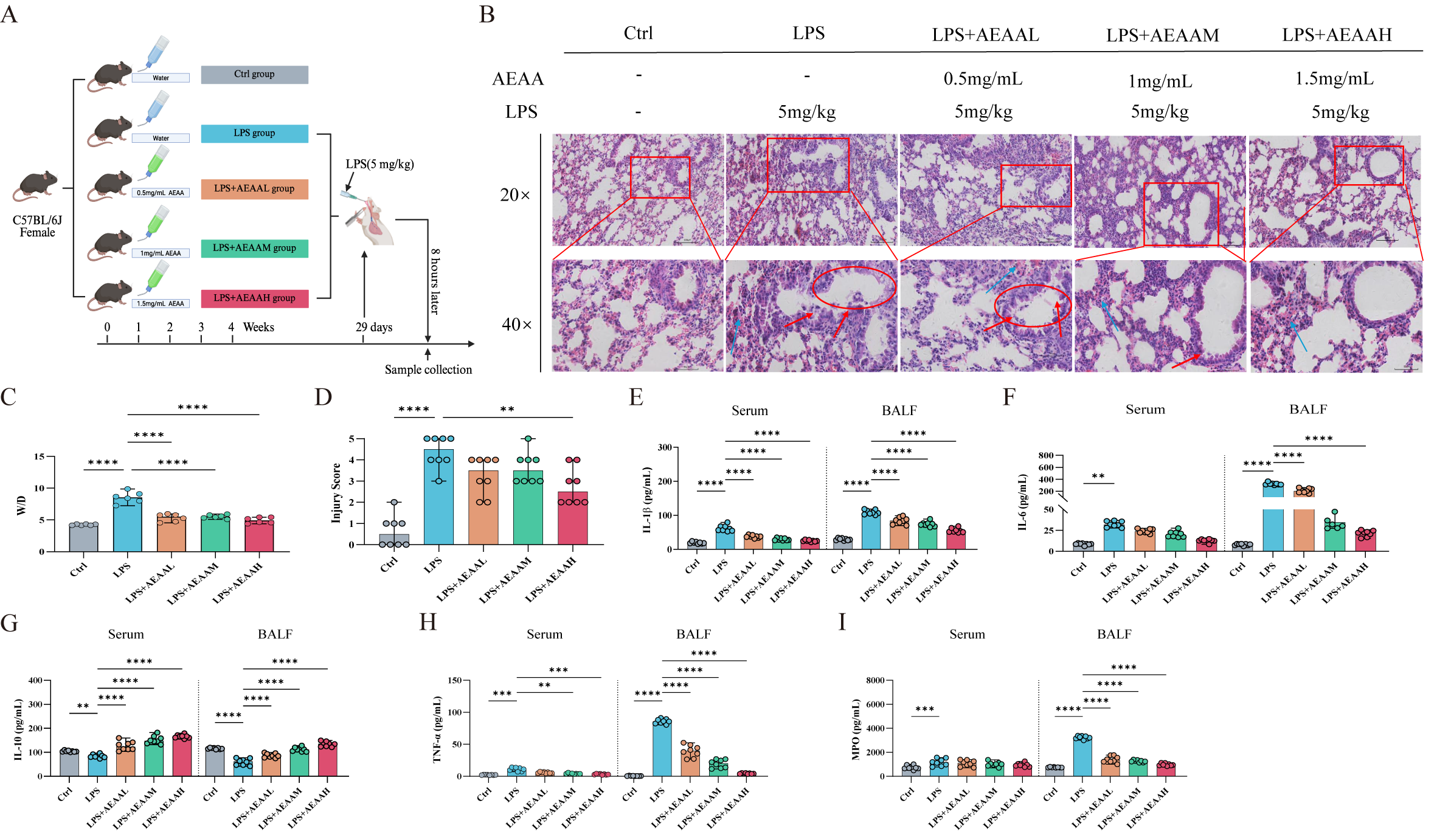

Supplement: Supplementary file 1 [file DataSheet1.zip › Data/Figure/Figure2.tif]

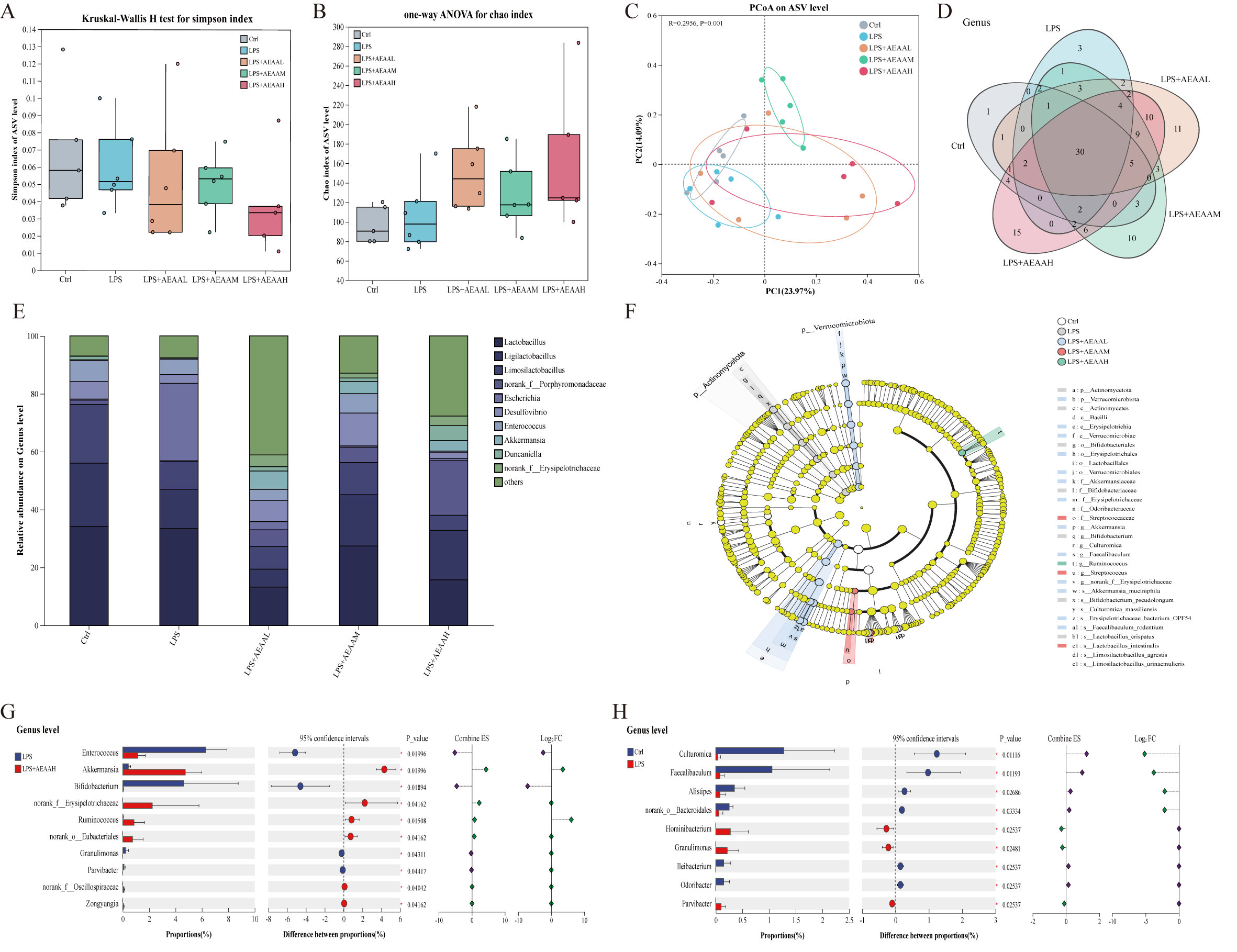

Supplement: Supplementary file 1 [file DataSheet1.zip › Data/Figure/Figure3.tif]

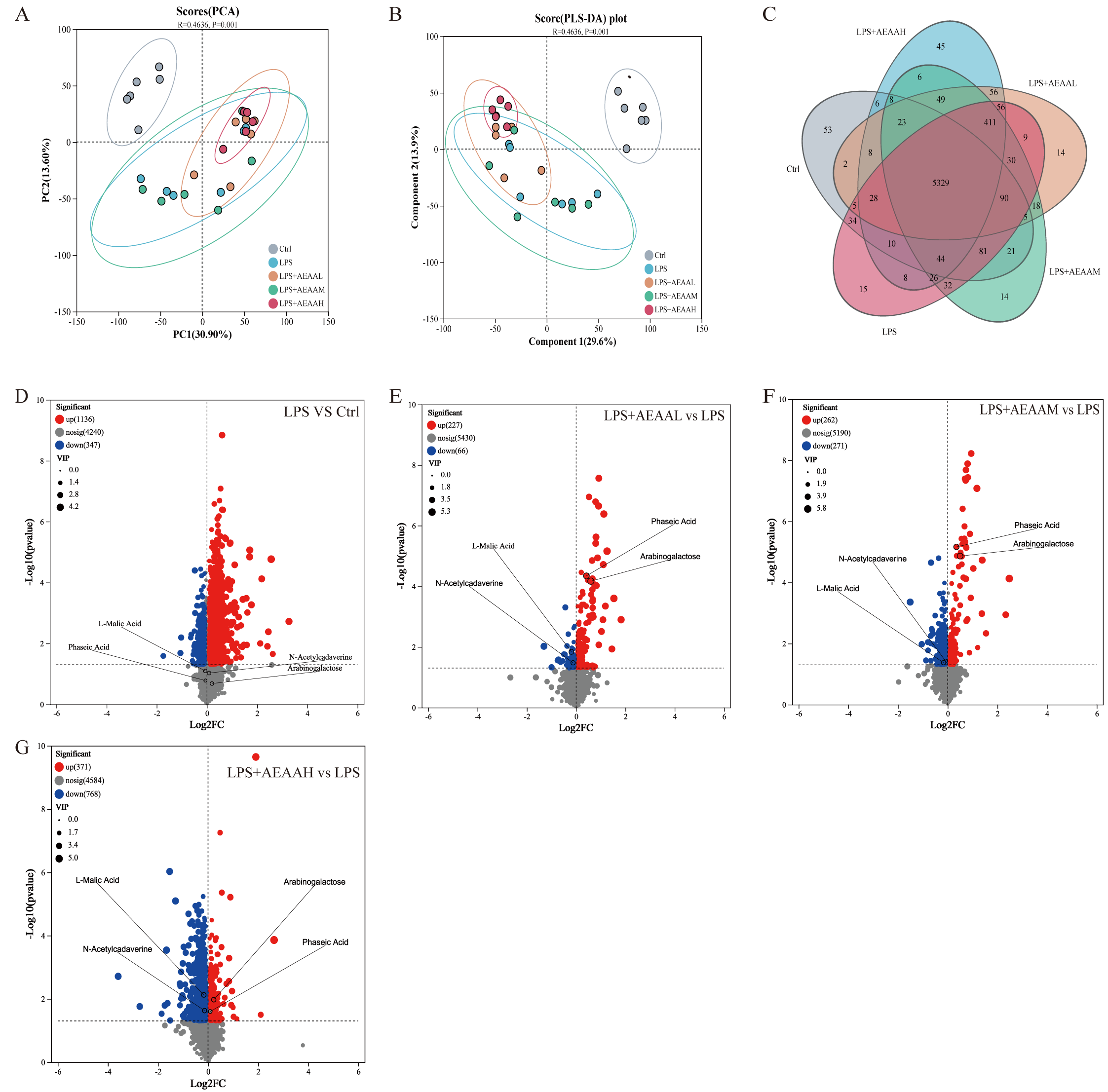

Supplement: Supplementary file 1 [file DataSheet1.zip › Data/Figure/Figure4.tif]

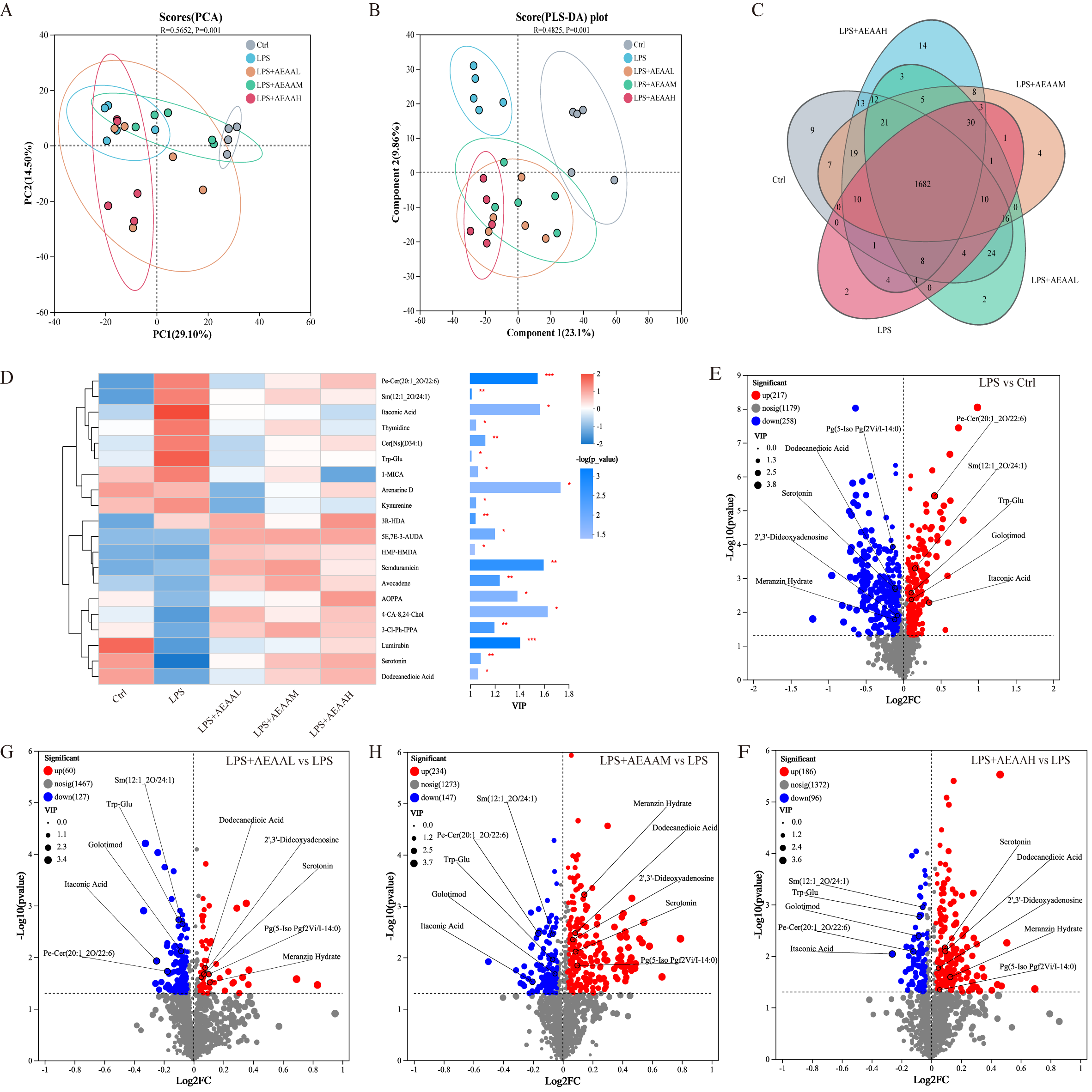

Supplement: Supplementary file 1 [file DataSheet1.zip › Data/Figure/Figure5.tif]

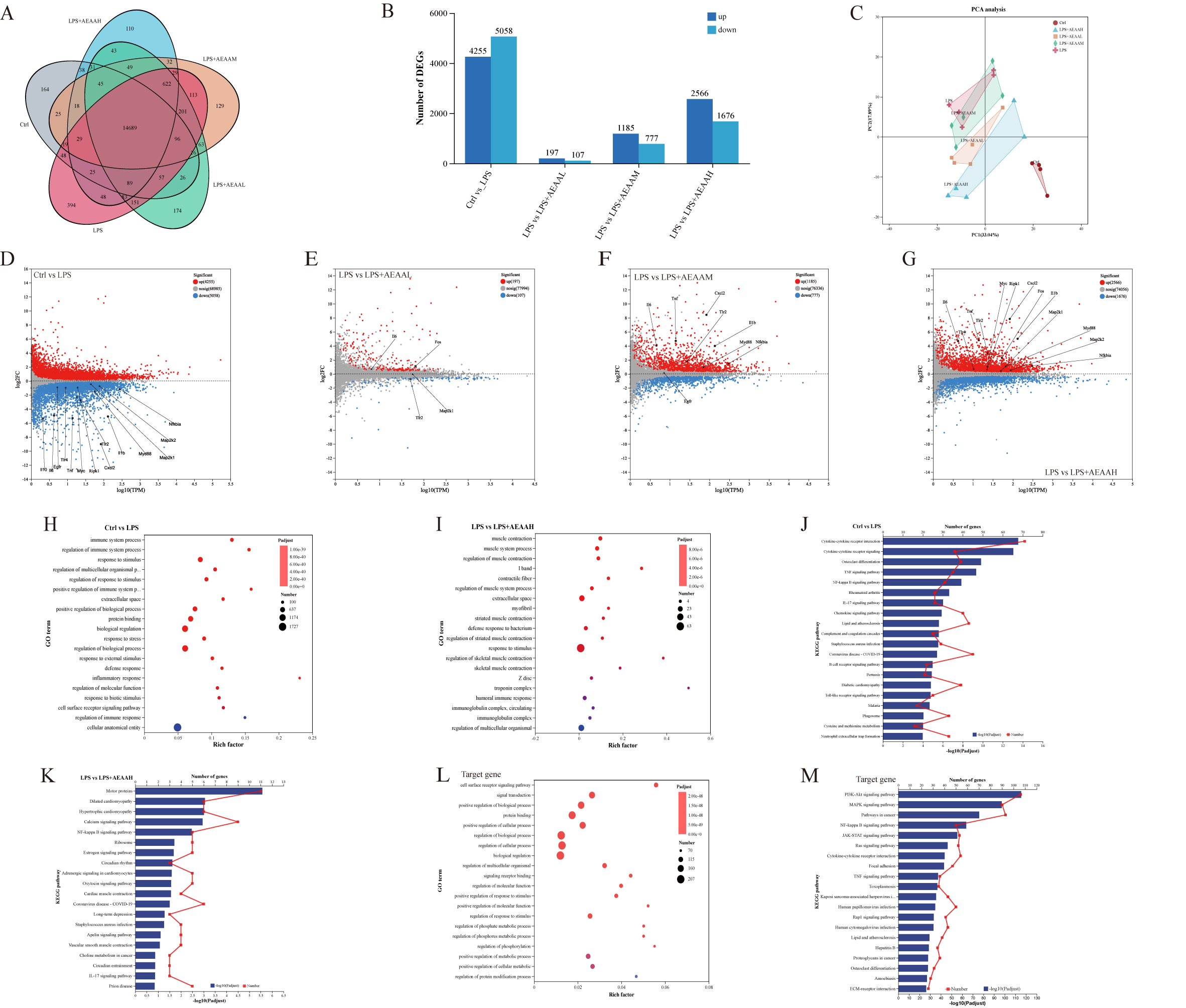

Supplement: Supplementary file 1 [file DataSheet1.zip › Data/Figure/Figure6-1.tif]

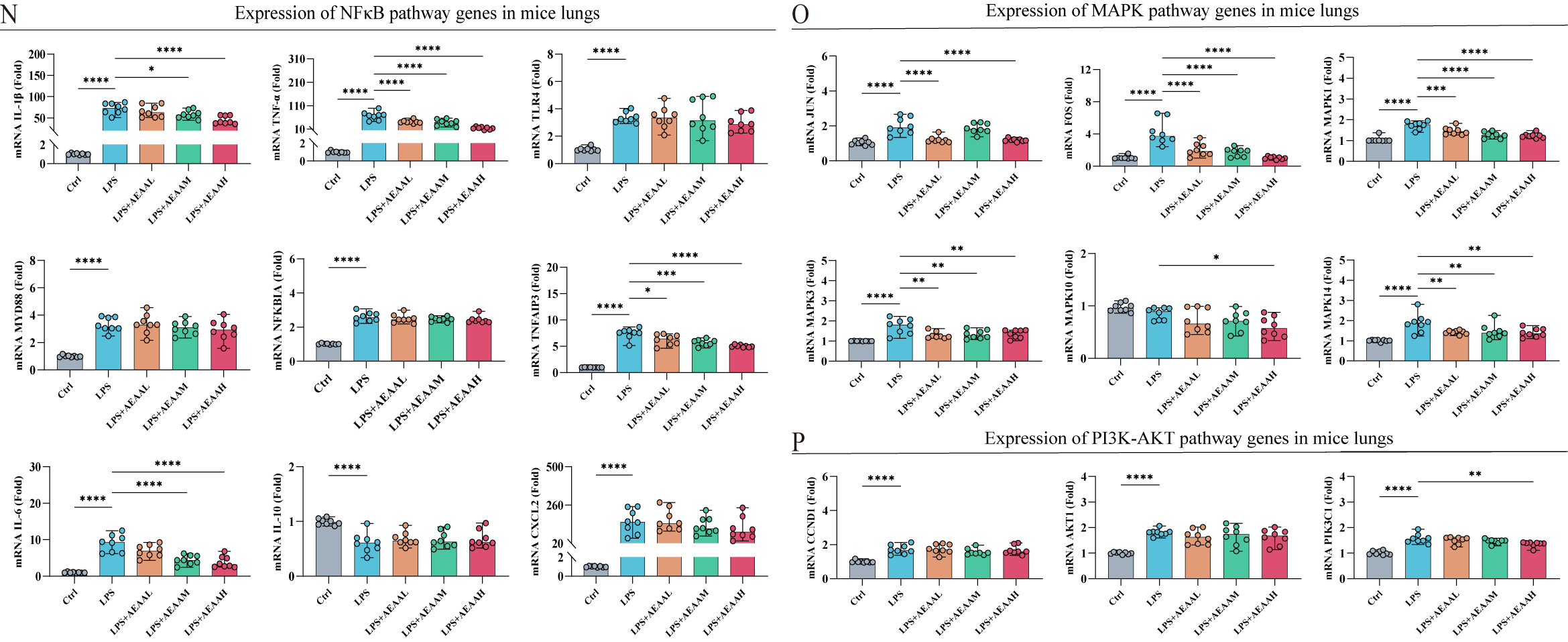

Supplement: Supplementary file 1 [file DataSheet1.zip › Data/Figure/Figure6-2.tif]

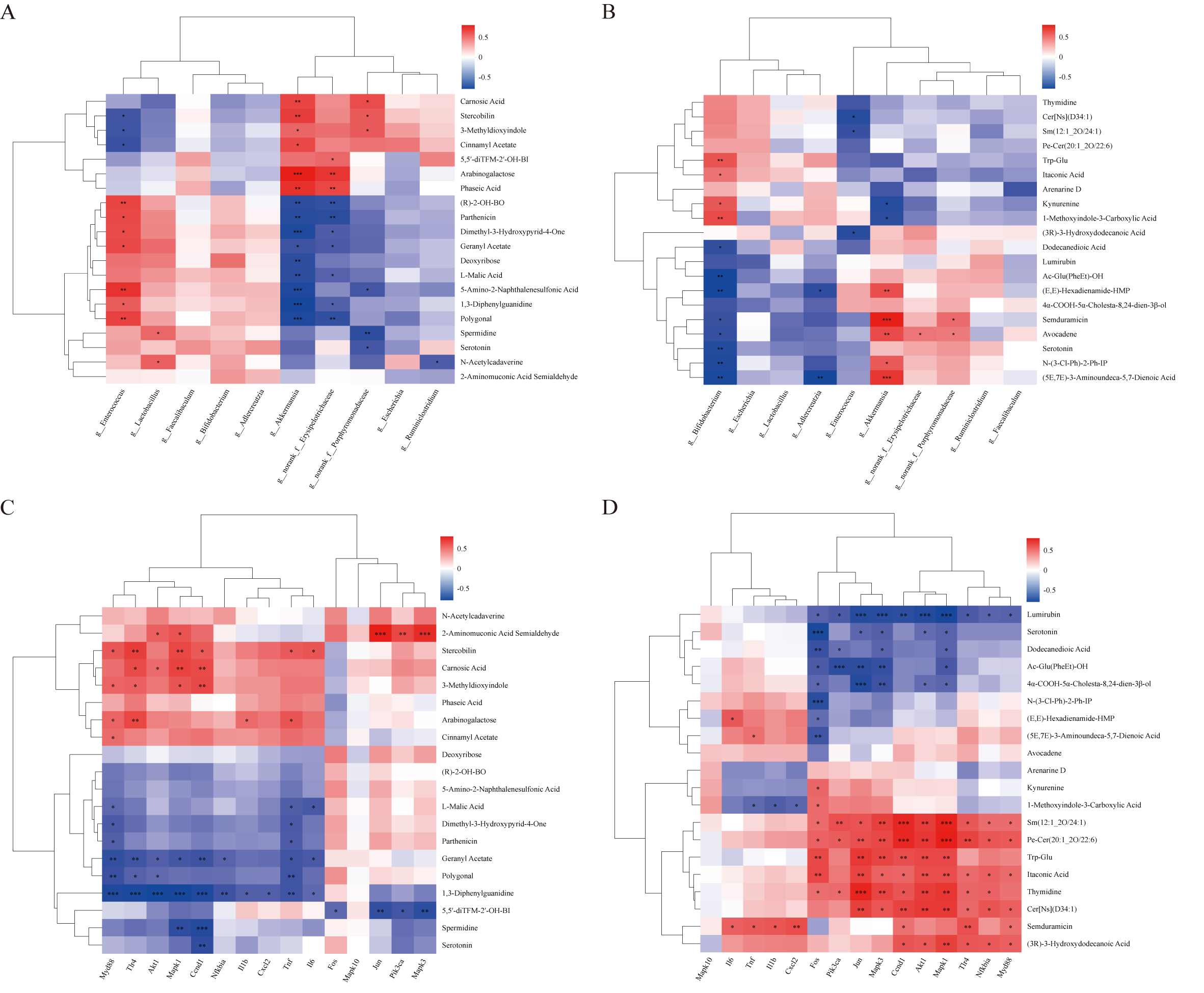

Supplement: Supplementary file 1 [file DataSheet1.zip › Data/Figure/Figure7.tif]
