# Supplementary material for: Aqueous Artemisia argyi extract mitigates acute lung injury in association with coordinated alterations in gut microbiota, metabolic homeostasis, and pulmonary inflammatory gene expression
Source: Front Immunol. 2026 Apr 2;17:1770675. doi: 10.3389/fimmu.2026.1770675 (PMC13082973; doi:10.3389/fimmu.2026.1770675)
Supplement: Supplementary file 1 [file MPO.pdf › Data/Figure/fig2/MPO.pdf]

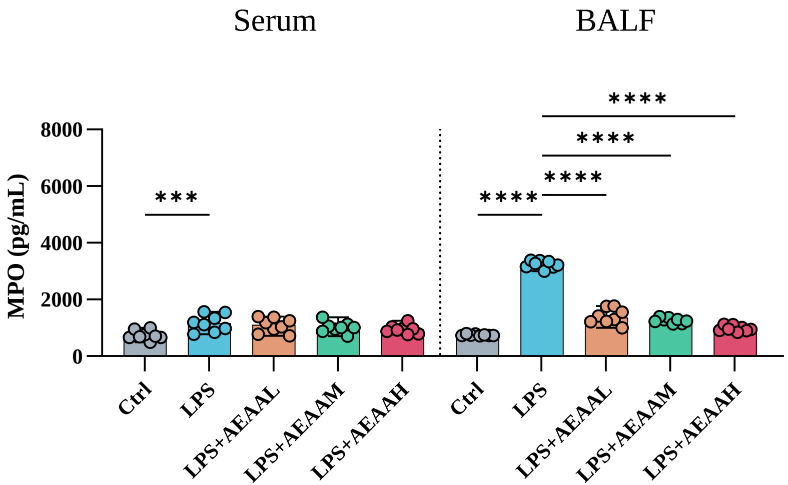

Serum
BALF
MPO (pg/mL)
8000
6000
4000
2000
0
***
****
****
****
****
Ctrl
LPS
LPS+AEAAL
LPS+AEAAM
LPS+AEAAH
Ctrl
LPS
LPS+AEAAL
LPS+AEAAM
LPS+AEAAH
